# Quantitative anatomy and biophysical modeling of ascending neuromodulatory systems in the developing rat neocortex

**DOI:** 10.1371/journal.pcbi.1014460

**Published:** 2026-06-26

**Authors:** Cristina Colangelo, Alberto Muñoz, Alberto Antonietti, Vishal Sood, Alejandro Antón-Fernández, Joni Herttuainen, Armando Romani, Javier DeFelipe, Srikanth Ramaswamy

**Affiliations:** 1 Blue Brain Project, École polytechnique fédérale de Lausanne (EPFL), Campus Biotech, Geneva, Switzerland; 2 Laboratorio Cajal de Circuitos Corticales, CTBUniversidad Politécnica de Madrid, Spain; 3 Department of Cell Biology, Complutense University, Madrid, Spain; 4 Instituto Cajal, CSIC, Madrid, Spain; 5 Department of Electronics, Information and Bioengineering, Politecnico di MilanoMilano, Italy; 6 CIBERNED, Centro de Investigación Biomédica en Red de Enfermedades Neurodegenerativas, Spain; 7 Neural Circuits Laboratory, Biosciences Institute, Newcastle University, United Kingdom; University of Surrey, UNITED KINGDOM OF GREAT BRITAIN AND NORTHERN IRELAND

## Abstract

The hindlimb representation in the somatosensory cortex of two-week old Wistar rats has been a valuable model system for dissecting the microcircuitry of neurons and their synaptic connections. In this study, we present a comprehensive experimental dataset quantifying the fiber length per cortical volume and the density of varicosities for cholinergic, catecholaminergic, and serotonergic neuromodulatory systems within the cortical neuropil using immunocytochemical staining and stereological techniques, along with a methodological framework for generating biophysically detailed computational models from these data. Acquired data were integrated into a biophysically detailed computational model of the somatosensory cortex to explore the anatomical organization and functional implications of neuromodulatory innervation. We found that neuromodulatory innervation, although sparse, substantially impacts network activity. Network simulations support the hypothesis that acetylcholine suppresses slow oscillations and promotes the desynchronization of cortical networks, consistent with the extensive findings in existing literature. Additionally, the temporal properties of acetylcholine modulation are consistent with synaptic rather than volume release. Furthermore, we found that the release of dopamine and serotonin in sensory cortices induces network desynchronization by inhibiting delta oscillations and that serotonin also initiates the emergence of theta oscillations, pointing to previously unexplored aspects of their function in governing cortical network activity. The experimental data and the biophysical computational model are available as an open-access community resource.

## Author summary

Brain activity is strongly influenced by neuromodulators, chemical compounds such as acetylcholine, dopamine, and serotonin, that regulate how neurons communicate and how brain states change. Although these systems are known to play key roles in attention, sleep, and arousal, it has been difficult to understand how their anatomical organization translates into effects on large-scale brain activity. In this study, we combined experimental measurements and computational modeling to investigate how neuromodulatory fibers are organized in the developing rat somatosensory cortex and how they influence network dynamics. We first quantified the density and distribution of cholinergic, dopaminergic, and serotonergic fibers and their release sites across cortical layers. We then integrated these data into a detailed computational model of cortical microcircuits, allowing us to simulate how neuromodulator release affects thousands of neurons simultaneously. Our simulations show that even sparse neuromodulatory inputs can strongly alter cortical activity by suppressing slow oscillations and promoting desynchronized or faster rhythmic states. We also reveal important differences between synaptic and volume modes of neuromodulator transmission. Together, our results provide a framework for linking the anatomy of neuromodulatory systems to their functional impact on brain activity and generate testable predictions for future experiments.

## Introduction

Neuromodulatory (NM) systems, particularly those involving acetylcholine (ACh), have been extensively characterized in the neocortex. Along with other neuromodulators, ACh plays a crucial role in augmenting cognition and facilitating transitions between brain states, which is fundamental to cortical function. ACh is well-known for its involvement in desynchronizing circuit activity and supporting transitions between brain states such as sleep to wakefulness or non-REM to REM sleep. It also enhances circuit excitability and responsiveness to stimuli [1]. Despite extensive research, the mechanisms underlying ACh’s desynchronizing effects remain unclear.

The complete dynamics of NM release have not been elucidated yet, likely because they can be influenced by a variety of factors. ACh can be released tonically or phasically, either via volume or synaptic transmission, and is subjected to fast clearing from the extracellular space due to the activity of catalytic enzymes such as cholinesterases [2,3]. Neuromodulators have traditionally been thought to act with low spatial precision throughout the cortex [4], but recent evidence suggests that they also exhibit fast modes of signaling [5–8] Furthermore, axons establish specialized synaptic contacts [9,10,11]. In the mouse hippocampus spatially homogeneous ACh signaling was observed spatially across volumes spanning hundreds of microns [12]

In the rodent neocortex, reports on the percentage of cholinergic varicosities that establish synaptic contacts are conflicting, with values ranging from 14% to 66% [11]. Some authors argue that cholinergic synapses might be difficult to identify with traditional methods, and propose novel techniques for their identification [9,10,11]. For instance, Takács and colleagues observed that cholinergic contact sites in the rodent neocortex were strongly labeled with neuroligin-2 and did not resemble typical synapses, suggesting that cholinergic fibers may establish more synaptic connections than previously recognized [13,14,15,16]. Overall, anatomical evidence suggests that cholinergic synapses exist, with apposition of pre- and postsynaptic sites, although they might not account for all ACh release sites [11,17].

Evidence in favor of cholinergic volume transmission (VT), such as the presence of extrasynaptic receptors and slowly decaying current kinetics, has also been reported [13,16,18]. On the other hand, Kalmbach et al. [5] showed that ACh enhances glutamatergic synaptic transmission in cortex via presynaptic nicotinic receptors in a synapse-specific, point-to-point manner rather than through global neuromodulation. ACh can function very precisely, selectively enhancing transmission at specific cortical synapses rather than acting as a diffuse neuromodulatory signal. According to Hay et al. [18] and others, both synaptic and diffuse cholinergic transmission occur in the neocortex depending on the regime of basal forebrain neuronal activity. Thus, even though fast point-to-point modulation of synaptic transmission has been repeatedly demonstrated in the rodent neocortex [5], the possibility that NM systems exert their action via volume transmission as well cannot be excluded.

Incidentally, while *en passant* axonal varicosities of cholinergic neurons from subcortical origins can mediate VT broadly in the cortex, acetylcholinesterase (AChE) restricts the diffusion of ACh by enzymatic hydrolysis after its release [14]. Hence, the initiation of diffuse transmission is likely subject to spatial and temporal constraints imposed by the catalytic activity of AChE, surpassing the influence of cholinergic receptor localization and density. Consequently, the regulatory processes on the receiving end ultimately refine the transmission, resulting in effects that are both spatially and temporally defined. Therefore, one intriguing facet of cortical neuromodulation concerns the long-standing debate about volume versus synaptic transmission, with evidence supporting both hypotheses. Ascending cholinergic, catecholaminergic, and serotonergic regulatory systems, comprising intricate networks of varicose fibers, modulate cortical microcircuits through both synaptic contacts (“classical” point-to-point chemical synapses) and non-synaptic diffuse or volume transmission processes, thereby influencing cortical states, functions, and development [19–21]. Nevertheless, resolving the debate regarding the proportion of synaptic versus non-synaptic connections established by these ascending modulatory systems requires moving beyond traditional 2D ultrastructural analysis. Conclusive evidence will depend on future large-scale 3D Electron Microscopy (EM) reconstructions to systematically map release sites across different brain regions.

To address this gap in the literature, we constructed a model representing both transmission types, aiming to elucidate the impact of their manipulation on our sensory circuit. While the multiscale effects of basal-forebrain cholinergic projections to the sensory cortex are extensively characterized, less is known about the activation of the dopamine (DA) and serotonin or 5-hydroxytryptamine (5-HT) modulatory systems and their multiscale effects on sensory microcircuits. Most of the data is recorded in prefrontal regions or in subcortical modulatory regions such as the striatum and the dorsal raphe (DR) [22,23,24]. However, the presence of both DA and 5-HT receptors has been reported in the rodent sensory cortex [25,26,27], and responses of cortical neurons to DA and 5-HT have been reported in slice preparations [28,29].

We reasoned that our modeling efforts could yield insights and extend the assessment of the influence of these NM systems in somatosensory regions. In this study, we estimated the densities and laminar distribution patterns of these three modulatory systems using immunostaining and stereological techniques. These data were then utilized to develop an advanced model of NM influences in the neocortex. This model builds on the previously reconstructed neocortical microcircuit framework by adding a simplified representation of neuromodulatory fibers in the sensory cortex, allowing for the activation of these fibers and assessment of their impact on neurons within the somatosensory network [30,31,32,33].

The simulations of cholinergic modulation were constrained by prior intracellular recording studies characterizing the cellular and synaptic effects of acetylcholine in cortical neurons. Whole-cell recordings in cortical slices have shown that ACh produces nicotinic depolarization and muscarinic modulation of intrinsic membrane conductances, including suppression of voltage- and Ca² ⁺ -dependent K⁺ currents and modulation of leak potassium conductances [34]. In addition, muscarinic receptor activation has been shown to produce presynaptic inhibition of both excitatory and inhibitory transmission [35,36,37,38]. These experimentally established effects on intrinsic excitability and synaptic efficacy provide the foundation for the more simplified conductance-based implementation of cholinergic modulation in the present model.

In this study, neuromodulatory influences are modeled at the level of receptor-mediated conductances rather than through direct modification of the intrinsic membrane channels of cortical neurons. Specifically, acetylcholine, dopamine, and serotonin release activates additional neuromodulator-sensitive conductances that are added to the postsynaptic neurons and parameterized to reproduce experimentally observed depolarizing or hyperpolarizing effects. This phenomenological approach captures the net functional impact on membrane potential and firing probability, while being implemented on top of the biophysically detailed neuron models of the underlying neocortical microcircuit [30], without re-optimizing or directly modifying their intrinsic membrane conductances. Importantly, this framework does not attempt to explicitly model the voltage or calcium-sensitive properties of membrane conductances or receptor subtype-specific intracellular signaling cascades, which remain insufficiently constrained across cell types and developmental stages. Instead, experimentally reported cellular responses are translated into conductance-level effects with defined kinetics and magnitude, allowing us to systematically study how anatomically grounded neuromodulatory input shapes network-level dynamics.

Through our modeling efforts, we extended the characterization of the neuropil via *in silico* analysis of the distribution and organization of NM fiber systems. This allowed us to predict missing biological information, such as the relative proportions of targeted neurons and the number of contacts established by each NM fiber. Our results show that ACh, 5-HT, and DA exhibit distinct patterns of innervation across cortical layers and neuron types, with DA having the broadest influence, innervating both excitatory and inhibitory neurons in all layers. We thus significantly refined our understanding of NM innervation, not only across cortical layers but also at the level of individual cell types. This approach also opened new avenues for investigating the non-synaptic effects of neuromodulatory signaling, allowing us to better quantify how volume transmission influences the proportions of affected cells.

We subsequently simulated the activation of ACh, DA, and 5-HT projections to model the synaptic and volume release of neuromodulators in the neocortical sensory microcircuit. This allowed us to evaluate the impact of NM inputs on somatosensory areas. Our findings confirm the desynchronizing effect of ACh and comparison with experimental data generates the additional suggestion that the dynamics are better matched by ACh synaptic transmission or weak volume transmission. Additionally, our simulations provide novel insights into the effects of DA and 5-HT in the sensory cortex. We find that both DA and 5-HT exert desynchronizing effects, with 5-HT specifically inducing faster oscillatory activity.

**Fig 1:**
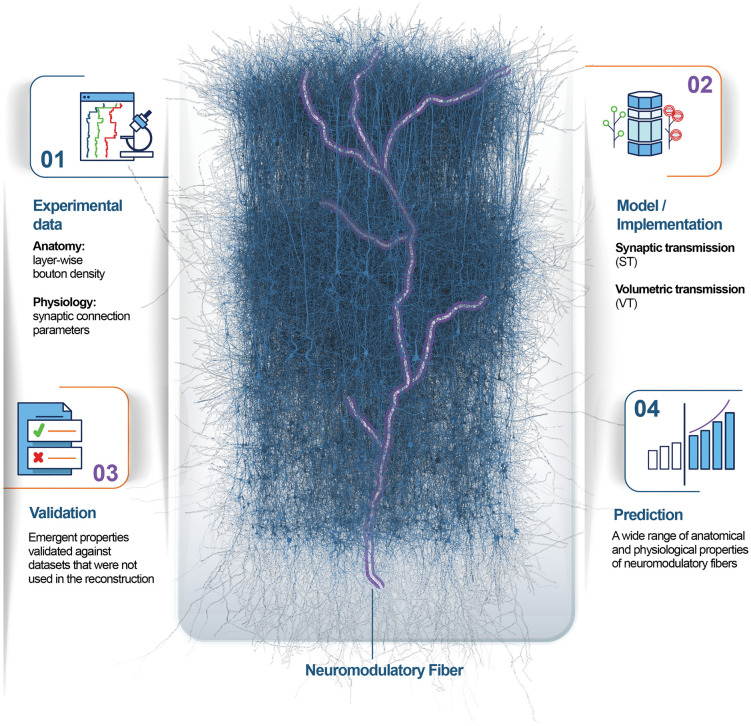
Neuromodulation overview. Reconstruction and simulation of NM innervation in a model of cortical microcircuitry. The workflow comprises the following steps. 1. Experimental data: we begin by gathering anatomical data on the layer-wise distribution of neuromodulatory fibers and density of varicosities, and physiological data on the impact of neuromodulatory release on cellular excitability and synaptic transmission. 2. Model implementation: we integrate the acquired data into a model of neuromodulatory projections with two distinct transmission types. 3. Validation: we validate the emergent properties of the innervated circuit against datasets that were not used in the reconstruction. 4. Prediction: thanks to the model, we can predict a wide range of anatomical and physiological properties of neuromodulatory systems that have been so far hard to measure.

Overall, this work presents a modeling framework that integrates available anatomical and physiological data on synaptic and non-synaptic transmission of neuromodulators in the neocortex (**Fig 1**). The framework is intended as a tool to systematically explore how different neuromodulatory systems may influence cortical dynamics under various conditions. By providing a structured approach to incorporate and test existing knowledge, the model can help generate hypotheses, identify inconsistencies in the literature, and guide future experimental investigations.

## Materials and methods

### Ethics statement

All experiments were approved by the local ethics committee of the Spanish National Research Council (CSIC) and performed in accordance with the guidelines established by the European Union regarding the use and care of laboratory animals (Directive 2010/63/EU).

### Animals

The experimental methods described here focus on characterizing the anatomical properties of neuromodulatory fibers in the rodent brain. Wistar rats (n = 11, aged 14 days) were sacrificed by administering a lethal intraperitoneal injection of sodium pentobarbital (40 mg/kg), and they were then perfused intracardially with saline solution followed by 4% paraformaldehyde in 0.1 M phosphate buffer (PB), pH 7.4. Brains were removed and post-fixed by immersion in the same fixative for 7 h at 4ºC. For the quantification of the cholinergic, catecholaminergic, and serotonergic fibers, after post-fixation, six brains (Rabb6-Rabb11) were cryoprotected in 30% sucrose solution in PB until they sank, frozen in dry ice, and cut in the coronal plane with a sliding freezing microtome. In all cases, 50 µm-thick sections extending in the antero-posterior axis from the anterior commissure to the rostral limit of the hippocampal formation and including the hindlimb representation area of the primary somatosensory cortex [39] were processed for immunocytochemistry.

### Immunocytochemistry

The sections were rinsed in PB, and to block non-specific antibody binding, they were preincubated for 1 h at room temperature in a stock solution containing 3% normal serum of the species in which the secondary antibodies were raised (Vector Laboratories, Burlingame, CA) in PB with Triton X-100 (0.25%). After preincubation, sections were incubated for 48 h at 4ºC in the same stock solution containing rabbit anti-serotonin (1:1000, Diasorin, Italy), mouse-anti-Tyrosine hydroxylase (TH, 1:1000, Diasorin), or goat-anti choline acetyltransferase (ChAT, 1:1000, Santa Cruz, CA, USA). Sections were then rinsed in PB and incubated in anti-rabbit, anti-mouse, or anti-goat biotinylated secondary antibodies (1:200; Vector Laboratories, Burlingame, CA). After rinsing in PB, a first set of sections of each type of immunostaining was processed for immunofluorescence being incubated for 2 h at room temperature in Alexa 488-coupled Streptavidin (1:200; Molecular Probes, Eugene, OR, USA). Sections were rinsed and stained with 4′,6-diamidino-2-phenylindole (DAPI) to reveal borders between layers and cytoarchitectonic areas. The sections were then washed in PB, mounted in antifade mounting medium (Invitrogen/Molecular Probes, Eugene, OR) and studied by conventional fluorescence and confocal microscopy (Zeiss, 710).

For confocal microscopy, stripes through the layers of the hindlimb primary somatosensory cortex were scanned from every animal. Z sections were recorded at 0.45 µm intervals through separate channels using a 40x oil-immersion lens (numerical aperture of 1.3). Subsequently, ZEN software (Zeiss) was used to construct composite images from each optical series by combining the images recorded through the different channels. Adobe Photoshop CS4 software was used to generate the figures (Adobe Systems Inc., San Jose, CA). For DAB immunostaining, a second set of sections was processed using the Vectastain ABC immunoperoxidase kit (Vector). Antibody labeling was visualized with 0.05% 3,39-diaminobenzidine tetrahydrochloride (Sigma, St Louis, MO) and 0.01% hydrogen peroxide. The sections were rinsed in PB and mounted on siliconized glass slides. After attachment, sections were lightly Nissl-stained with thionin, dehydrated, cleared with xylene, and the cover slipped. Controls were included in all the immunocytochemical procedures, either by replacing the primary antibodies with preimmune goat serum in some sections, by omitting the secondary antibodies, or by replacing the secondary antibody with an inappropriate secondary antibody. No significant immunolabeling was detected under these control conditions.

### Estimation of fiber length density

The fiber length per unit volume and the density of varicosities of the cholinergic, catecholaminergic, and serotonergic systems, DAB-immunostained through the depth of the tissue, were stereologically estimated in every cortical layer using respectively the space ball probe (dissector height of 11–18 µm) and the optical fractionator tool (dissector height of 11 µm in all cases) of Stereo Investigator software (StereoInvestigator 7.0, MicroBrightField Inc. Vermont, USA) following previous studies [40]. An oil immersion x100 objective (numerical aperture of 1.35) on a BX51 Olympus microscope equipped with a Prior motorized stage and a JVC video camera was used. The light Nissl staining of every immunostained section helped to distinguish areal and layer limits and to trace the contour lines corresponding to the individual cortical layers within the hindlimb cortex with the aid of an x20 objective. For each cortical layer, type of immunostaining and animal, the number of sampling sites performed and the number of sections used was determined by the constraints of maintaining the coefficient of error below 0.09 [41]. Space ball probe sampling prevents the values obtained for fiber length density from being biased by possible differences in the orientation of fibers relative to the tissue slicing plane. The fiber length density and the density of varicosities were corrected for shrinkage as brain tissue shrinks during processing. To estimate the shrinkage in our samples, we measured the surface area and thickness of the sections using Adobe Photoshop and Stereo Investigator software, respectively before and after tissue processing either for immunoperoxidase or immunofluorescence. The surface area after processing was divided by the value before processing to obtain an area shrinkage factor (p2) of 0.89. In addition, the obtained linear shrinkage factor in the z-axis (pZ) was 0.28. Therefore, the volume shrinkage factor (p3 = p2 ⋅ pZ) was 0.25.

### Synaptic model of NM release

To build a model of NM release involving synaptic transmission (ST), we used the Projectionizer tool. The Projectionizer had already been adopted to model the thalamo-cortical and cortico-thalamic projections described in related studies [30,33,42] and was specifically developed to model projections to the neocortical microcircuit in a way that satisfies experimental constraints (see **Table 1**). Data about the density of NM varicosities are used to constrain the addition of new synaptic release sites in the model; we instantiated a layer-wise varicosity density profile in the model that matches the experimentally measured density. The generation of NM projections is a multi-step workflow as described below:

**Table 1:** List of software used.

Software name	Source	Identifier
BluePySnap	BBP/EPFL software package	https://github.com/BlueBrain/snap
circuit-build	BBP/EPFL software package	https://github.com/BlueBrain/circuit-build
Core-Neuron	BBP/EPFL software package	https://github.com/BlueBrain/CoreNeuron
Elephant	Elephant authors	https://doi.org/10.5281/zenodo.1186602
Neurodamus	BBP/EPFL software package	https://github.com/BlueBrain/neurodamus
NeuroM	BBP/EPFL software package	https://github.com/BlueBrain/NeuroM
Projectionizer	BBP/EPFL software package	https://github.com/BlueBrain/projectionizer

1. **Sampling**: To place synaptic release sites (sRS), the Projectionizer tool samples postsynaptic dendritic segments from neurons already present in the reconstructed neocortical microcircuit, rather than fully reconstructed presynaptic axonal arbors. Specifically, all morphological dendritic segments contained within each cortical layer are pooled, and segments are repeatedly sampled with replacement to place new sRS at their centers. The probability of sampling a given segment is proportional to its length, such that longer dendritic segments are selected more frequently. This procedure ensures that the spatial distribution of sRS reflects both the laminar organization of the circuit and the available postsynaptic dendritic surface area.
2. **Assignment to fiber**: In the model, neuromodulatory projections are represented by simplified, straight virtual fibers that originate in layer 6 and extend vertically through all cortical layers. These fibers represent presynaptic source trajectories and are not intended to reproduce detailed axonal morphologies. After synaptic release sites are placed on postsynaptic dendritic segments, each sRS is assigned to one of these virtual fibers based on spatial proximity using a Gaussian distance distribution. Thus, while the fibers define the presynaptic organization and activation patterns of neuromodulatory inputs, the targets of neuromodulation are exclusively the dendritic compartments of postsynaptic neurons in the reconstructed microcircuit. Neuromodulatory fibers are modeled as straight trajectories for simplicity. Because release sites are assigned to fibers probabilistically based on spatial proximity, moderate deviations from straight geometry would not substantially affect connectivity statistics or network-level results.

Neuromodulatory connectivity is therefore inferred solely from experimentally measured varicosity densities and the spatial distribution of dendritic segments in the reconstructed circuit, without incorporating EM-based contact probabilities between neuron types.

1. **Parameter selection**: In this step, we select the parameters that are better suited to mimic NM connections. The underlying neocortical microcircuit uses conductance-based synaptic models described in Markram et al., [30], and in the Neocortical Microcircuit Collaboration Portal [31]. In the present work, we used this framework as a base and added simplified neuromodulatory conductances whose parameters were constrained from literature-reported data. We reasoned that since the reported number of cholinergic neurons in the rat nucleus basalis of Meynert (NBM) is 7,312 [43] and the NBM projects mainly to the primary somatosensory region (S1) [44,45] then there would be 7,312/ 26 = 281 fibers projecting to our reconstructed microcircuit, because the entire S1 area model is 26 times bigger (see **Methods** section - Microcircuit). Similarly, we assigned 2,651/ 26 = 102 fibers to the dopaminergic system, based on cell-counts obtained in the rat ventral tegmental area (VTA) [46] and estimations of the number of VTA neurons that project to S1 [47]. Lastly, we computed the number of serotonergic fibers; 11,500 serotonergic cell bodies have been counted in the dorsal raphe according to Descarries and others, and only ~12% project to the S1 region [48,49]. That is 1,380/ 26 = 53 5-HT fibers for the simulated circuit. The values obtained seem to fall within reasonable ranges, considering the estimated density of each NM system, but nevertheless they rest on assumptions that have not been proven. We refer the reader to the **Discussion** section where we list the most important assumptions.

**Fig 2:**
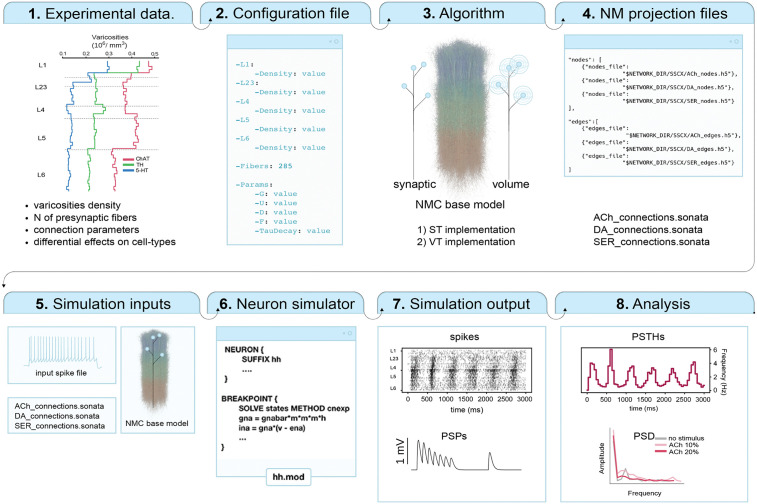
Modeling workflow. ST synaptic transmission; VT volume transmission; ACh acetylcholine; DA dopamine, 5-HT serotonin; NMC neocortical microcircuit model; PSP postsynaptic potential; PSTH peristimulus time histogram; PSD power spectral density. 1. We gather experimental data about the anatomy and physiology of neuromodulatory fibers. 2. We transform the data into parameters that will be summarized into a configuration file. 3. The configuration file is fed to the algorithm that reconstructs projections to the cortical circuit. Note that synaptic placement is constrained by postsynaptic dendritic geometry and experimentally measured varicosity densities, rather than by explicit presynaptic axonal reconstructions. 4. The algorithm outputs are the models of the projections: SONATA files that can be read by the simulator. 5. The projection files and the spike series files are used as inputs to the simulation pipeline together with the reconstructed circuit. 6. The inputs are read by the NEURON software to simulate the activation of neuromodulatory projections and their effect at the network level. 7. The simulator outputs neural activity such as spike times and post-synaptic potentials. 8. The outputs of the simulation pipeline are processed and analyzed to study circuit activity.

For the new NM connections, we used the synaptic type framework (s-types) defined in Markram et al., [30] as a structuring approximation for short-term dynamics, while neuromodulator-specific decay time constants (DTCs) were constrained from the literature (see **Table 2**). The complete modeling workflow is reported in **Fig 2**.

**Table 2:** Neuromodulatory projections parameters.

ACh	L5 and L6PCs	L23 and L4 PCs	L1 INTs, L23 BPs, BTCs, DBCs, SBCs, MCs, NGCs	L23 CHCs, LBCs, NBCs
**Synaptic DTC**	241.2 ± 15.5 ms	241.2 ± 15.5 ms	241.2 ± 15.5 ms	241.2 ± 15.5 ms
**Volume DTC**	608.6 ± 109.7 ms	608.6 ± 109.7 ms	608.6 ± 109.7 ms	608.6 ± 109.7 ms
**E**_**rev**_	0 mV	-80 mV	0 mV	-80 mV
**G**_**max**_	0.31 ± 0.11 nS	0.66 ± 0.15 nS	0.3 ± 0.11 nS	1.4 ± 1.6 nS
**U**_**SE**_	0.5 ± 0.011	0.3 ± 0.043	0.86 ± 0.048	0.23 ± 0.093
**D**	670 ± 9.3 ms	1300 ± 280 ms	670 ± 9.1 ms	600 ± 350 ms
**F**	17 ± 2.8 ms	2.2 ± 1.9 ms	17 ± 2.7 ms	33 ± 20 ms
**s-type**	E2	I2	E2	I3
**DA**	**L5 and L6PCs**	**L23 and L4 PCs**	**L1 INTs**	**L23-L6** **CHCs, LBCs, NBCs, SBCs**
**Synaptic DTC**	220.2 ± 41 ms	220.2 ± 41 ms	220.2 ± 41 ms	220.2 ± 41 ms
**Volume DTC**	400 ± 100 ms	400 ± 100 ms	400 ± 100 ms	400 ± 100 ms
**E**_**rev**_	0 mV	-80 mV	0 mV	0 mV
**G**_**max**_	0.31 ± 0.11 nS	0.66 ± 0.15 nS	0.3 ± 0.11 nS	0.3 ± 0.11 nS
**U**_**SE**_	0.5 ± 0.011	0.3 ± 0.043	0.86 ± 0.048	0.22 ± 0.24
**D**	670 ± 9.3 ms	1300 ± 280 ms	670 ± 9.1 ms	390 ± 240 ms
**F**	17 ± 2.8 ms	2.2 ± 1.9 ms	17 ± 2.7 ms	300 ± 240 ms
**s-type**	E2	I2	E2	E1
**5-HT**	**L5 and L6PCs**	**L1 INTs**	**L23 CHCs, LBCs, NBCs**	**L23 BPs, BTCs, DBCs, SBCs, MCs, NGCs**
**DTC**	440 ± 30 ms	440 ± 30 ms	440 ± 30 ms	440 ± 30 ms
**E**_**rev**_	-80 mV	0 mV	-80 mV	0 mV
**G**_**max**_	0.66 ± 0.15 nS	0.3 ± 0.11 nS	1.4 ± 1.6 nS	0.3 ± 0.11 nS
**U**_**SE**_	0.3 ± 0.043	0.86 ± 0.048	0.23 ± 0.093	0.09 ± 0.062
**D**	1200 ± 280 ms	670 ± 9.1 ms	600 ± 350 ms	140 ± 110 ms
**F**	2.2 ± 1.9 ms	17 ± 2.7 ms	33 ± 20 ms	670 ± 430 ms
**s-type**	I2	E2	I3	E1

DTC: decay time constant of the PSC (values were obtained from [50, 22, 18, 9]); E_rev_: reversal potential; G_max_: maximal conductance; U_SE_: utilization of synaptic efficacy; D: time constant of depression; F: time constant of facilitation. The synaptic class (s-type) designation follows the framework of Markram et al. [30], whereas parameter values were constrained by neuromodulator-specific experimental studies where available. Examples of s-types in intra-cortical synapses: E1, E2, I2, I3.

### Volume model of NM release

Our model of volume NM transmission (VT) relies upon the implementation of the synaptic model explained above. To model VT, we first took the experimentally recorded varicosity density profile, and we instantiated new release sites (RS) in order to match this density. Then, every RS was designated as the center of a sphere of radius R_max_, which is the sphere of influence of volume transmission whose values were taken from literature assuming that the influence of the signal for R > R_max_ is negligible (thus the RS becomes a volume RS, or vRS). To select an appropriate volume of influence for the NM volume signal, we searched for studies reporting the spatial extent of NM transients; often these are quite recent studies that leverage recently developed genetically encoded fluorescent sensors. To instantiate the ACh VT model, we assigned a value of 5 µm to the R_max_ parameter, which was kept fixed for all simulations of volume cholinergic release, as reported by Borden et al. [25]. We used the value computed by Borden and colleagues, because it was recorded in the rodent neocortical brain areas, as opposed to other values obtained in the medial entorhinal cortex or the retina, which nevertheless are in a similar range [25,51,52].

Dopaminergic transients have also been measured by means of a synthetic catecholamine nanosensor which revealed DA hotspots with a median size of 2 µm [53] so we instantiated R_max_ = 2 µm for DA connections, even though this data was recorded in the striatal region. 5-HT’s volume influence was estimated by [54] who recorded serotonergic extrasynaptic transmission via carbon fiber microelectrodes in the rat DR; thus, we selected R_max_ = 3 µm for 5-HT signals. Subsequently all morphological segments within the sphere are sampled and a new conductance is instantiated in a random position along each segment. Here we assume that extrasynaptic cholinergic receptors are equally distributed across neuronal neurites. In this case the vRS is the source of the NM signal, and the segment where the new conductance is placed is the target. Still, these two do not coincide like in the synaptic implementation of NM release.

Thus, we developed a way to mimic the one-source-to-many-targets characteristic of VT. The new volume connections are parametrized in the same way as the synaptic ones (see above), but the conductance values in this case are scaled according to the distance from the vRS. Here, we assume that the NM signal spreads from the vRS in a wave-like manner, evenly in all directions. At a distance *r* from the vRS, the NM signal reaches its peak concentration after a delay that depends on *r*. In our model, we ignore the time-course of the NM wave and focus solely on its peak concentration. We make the peak concentration inversely proportional to *r*, ranging from 1 at the vRS to 0.1 at R_max_. We also disregard any delay between the source and the target; activation occurs simultaneously for all target cells and follows the same time course. Specifically, we determine a scaling factor whose value ranges from 0.1 to 1 and is inversely proportional to the distance from the vRS. Here, we assume that the strength of the volume signal decreases linearly with the distance from the release site to have a simple model of ACh diffusion and catalysis. The literature reported values for the offset decay kinetics of ACh currents range across several orders of magnitude [9,18,55]. The DTC values (**Table 2**) are important to determine the transmission timeline in both synaptic and volume release. While NM ST is known to act on rapid timescales (i.e., the millisecond (ms) range), VT works with significantly longer transmission delays (hundreds of milliseconds) [55,56]. Nevertheless, synaptically mediated cholinergic currents with a slower decay have been observed as well [18]. We refer the reader again to **Table 2** for a more specific description of the DTC values used to parametrize the kinetics of VT.

### Microcircuit

Our *in silico* neuromodulation model was implemented in a digital microcircuit extracted from a large-scale model covering the entirety of the non-barrel primary somatosensory cortex of the juvenile rat [33,57]. The microcircuit is 26 times smaller than the whole S1 region and comprises 163,528 cells. The simulated network consists of seven cortical microcircuits arranged side by side in the horizontal plane to form a tiled cortical sheet. Each microcircuit is represented as a hexagonal prism whose dimensions are determined by layer thicknesses and column diameter.

### Network simulations

Simulations were conducted using the open-source software Neurodamus (see **Table 1**) based on the NEURON simulation environment. Data were output in the form of binary files containing spike times sampled every 0.1 ms for each neuron in the network. Extracellular calcium and potassium concentrations were modeled considering their phenomenological effects on neurotransmitter release probability and somatic depolarization, respectively. In particular, we used a Hill function to nonlinearly scale the release probability of the synapses with the extracellular calcium concentration. To model the effect of extracellular potassium ion concentration we injected a constant current into the somatic compartment of each neuron (~95% relative to each neuron’s rheobase current). These values were adjusted to mimic an *in vivo*-like network state, corresponding (empirically) to extracellular calcium and potassium concentrations of 1.7 mM, and 5.0 mM, respectively.

Simulations were conducted near the transition between a synchronous and an asynchronous network regime. In this study, the synchronous state refers to a condition characterized by coherent slow oscillations (~2 Hz), approximating an inactivated or sleep-like cortical state. As previously done in Markram et al., [30], this state was obtained by increasing network excitability through a constant somatic depolarizing current applied to all neurons, bringing cells close to, but below, their firing threshold. This manipulation is a phenomenological approximation of the effect of elevated extracellular potassium used in the underlying microcircuit framework; extracellular potassium dynamics themselves were not explicitly modeled. The synchronized state was not defined by a single membrane potential value, but by a range of depolarizing drive values over which the network produced stable slow oscillations. Changes in this depolarizing drive can alter the stability and frequency of the oscillatory regime and eventually shift the network toward asynchronous activity. All neuromodulatory stimulations were applied starting from this baseline synchronous condition. Every simulation was repeated 30 times with different seeds (tied to random number generators that we used in our simulation pipelines) to reproduce trial variability, and a power density analysis was performed to evaluate the frequency contents of the oscillatory activity of our simulated network of neurons. The stimulus to the NM projections was delivered as a train stimulation of frequencies of approximately ~20 Hz. To recruit different proportions of ascending inputs we selected varying percentages (from 10 to 100%) of the afferent fibers. The stimulus was delivered at t = 2000 ms and was applied for 2000 ms.

### Supercomputing

A 2-rack Intel supercomputer using dual socket, 2.3 GHz, 18 core Xeon SkyLake 6140 CPUs, with a total of 120 nodes, 348 GB of memory, and 46 TB of DRAM was used to run the simulations and perform analysis.

### Simulation outputs analysis

All code for analysis was written in Python 3.6. To estimate the power of the signal at different frequencies, we performed a power spectral density (PSD) analysis by calculating firing rate frequencies and subsequently applying the Welch transformation. Peri-stimulus time histograms (PSTHs) were computed from all neurons in the extracted microcircuit and were normalized by neuron number and time bin to express the average instantaneous firing rate (bin size = 10 ms). The estimation was performed on the first 1000 ms time interval after the stimulus delivery. To quantify the delta power, we integrated the spectral power across the 1.5-3 Hz frequency band. No additional time-domain band-pass filtering was applied.

### Statistical analysis

To estimate possible differences in the length and density values obtained in the different cortical layers, a non-parametric test was performed and correction for multiple comparisons was applied. First, we used Friedman’s test, and we found that length and varicosity densities differed across layers; post-hoc analysis (Conover’s test) was later performed to compare all layers for the three NM systems. Bonferroni’s correction was applied to account for multiple comparisons. To assess the statistical significance of the PSD analysis results, we used a paired T-student test.

## Results

### Cholinergic fibers

**Fig 3:**
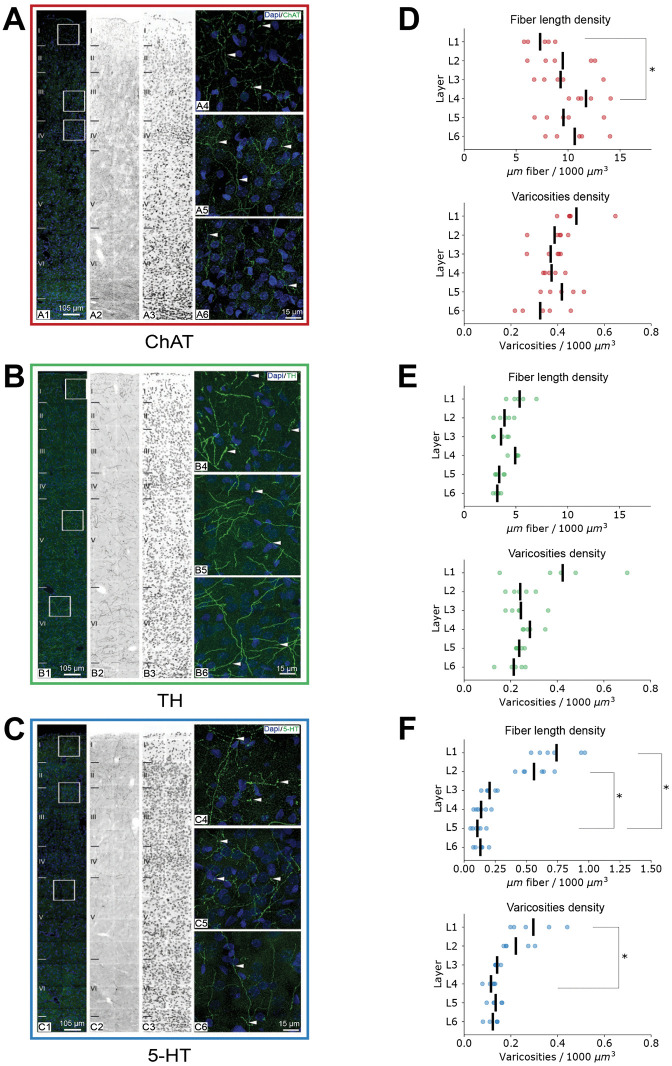
ChAT, 5-HT and TH immunoreactivity. **A)** confocal stack projection image, corresponding to a cortical thickness of 14 µm, showing the distribution of ChAT-immunoreactive fibers (green) in the different layers, as revealed by DAPI staining (blue), of the P14 rat hindlimb somatosensory cortex. A2) and A3) show respectively, in monochrome images, ChAT immunostaining and DAPI staining. Squared zones in A are shown at higher magnification in A4-A5-A6. Arrowheads point to fiber varicosities. **B)** same as in A) but for tyrosine-hydroxylase (TH)-immunoreactive fibers. **C)** same as in A) but for 5-HT-immunoreactive fibers. **D)** Graph showing the mean values obtained in the HLS1 of the five P14 rats analyzed for the density of fibers (above) and the density of varicosities (below) for the cholinergic system. **E)** same as in D but for the catecholaminergic system. **F)** same as in D but for the serotonergic system.

In addition to sparsely distributed ChAT-immunoreactive (ir) neuronal cell bodies distributed from layers 2–6 (**S1 Fig**), ChAT immunostaining revealed the presence of an intricate network of varicose fibers across all cortical layers of the rat P14 HLS1 (**Fig 3A** and **Table 3**). Unfortunately, at a distance from the cell body, ChAT-ir positive processes emanating from the cell body of cortical cholinergic neurons could not be distinguished from the surrounding positive elements, most of which are known to originate in the basal forebrain [58]. Fibers throughout cortical layers were oriented in all directions; however, in layers 1 and 6, fibers with an orientation parallel to the pial surface were frequently found. The density of cholinergic fibers was relatively homogeneous across the cortical thickness, with statistically significant differences only observed between layers 1 and 4 (P-value = 0.048) (**Fig 3D**). Fiber varicosities were also distributed homogeneously, with no significant differences between the different cortical layers (**Fig 3D**).

**Table 3:** Fiber length per cortical volume and density of varicosities.

Layer	5-HT-ir fibers		TH-ir fibers		ChAT-ir fibers	
	Length	Varicosities	Length	Varicosities	Length	Varicosities
**Mean ± SD**	µm/ 1000 µmᶟ	Var/ 1000 µmᶟ	µm/ 1000 µmᶟ	Var/ 1000 µmᶟ	µm/ 1000 µmᶟ	Var/ 1000 µmᶟ
**1**	0.74 ± 0.17	0.29 ± 0.10	5.36 ± 1.08	0.42 ± 0.19	7.30 ± 1.29	0.47 ± 0.09
**2**	0.56 ± 0.12	0.22 ± 0.06	3.89 ± 0.76	0.24 ± 0.04	9.48 ± 2.82	0.38 ± 0.06
**3**	0.20 ± 0.04	0.14 ± 0.009	3.57 ± 0.7	0.24 ± 0.06	9.27 ± 2.55	0.36 ± 0.06
**4**	0.13 ± 0.05	0.11 ± 0.02	4.92 ± 0.47	0.28 ± 0.03	11.72 ± 1.52	0.37 ± 0.03
**5**	0.10 ± 0.04	0.13 ± 0.039	3.38 ± 0.56	0.23 ± 0.02	9.55 ± 2.72	0.41 ± 0.11
**6**	0.13 ± 0.04	0.12 ± 0.02	3.20 ± 0.29	0.21 ± 0.05	10.63 ± 2.40	0.32 ± 0.09

Fiber length per cortical volume and density of varicosities of serotonergic, catecholaminergic, and cholinergic regulatory systems in HLS1 at P14. Measured data were corrected for shrinkage and refer to the total volume of cortical layers.

### Catecholaminergic fibers

Immunocytochemistry for tyrosine hydroxylase (TH), the rate-limiting catecholamine synthesizing enzyme, revealed the presence of groups of aspiny non-pyramidal neurons. According to previous studies [59], these cells are distributed across all cortical layers, with the highest abundance in layers 2–3 (**S1 Fig**). The processes arising from this neuronal population might therefore contribute to the TH-ir fibers quantified in the present study (**Fig 3B** and **Table 3**). In addition, TH immunoreactivity primarily labels dopaminergic fibers in the cerebral cortex [60,61,62]. However, the possibility that our results include noradrenergic fibers and varicosities, which are mainly labeled with dopamine-β-hydroxylase [63], cannot be excluded. In the rat HLS1 neocortex, layers 1 and 4 exhibited the highest density of TH-ir fibers, followed by layers 2 and 3, with the lowest density observed in layers 5 and 6 (**Fig 3E**). However, no statistical differences were found between layers in the density of TH-ir fibers. A similar laminar distribution pattern was observed forTH-ir varicosities, with no statistical differences between layers (**Fig 3E**).

### Serotonergic fibers

Immunocytochemistry for 5-HT revealed the presence of numerous 5-HT-ir fibers through all cortical layers (Fig 3C and Table 3). No immunoreactive cell bodies were found, as expected. A higher density of serotonergic fibers was found in supragranular layers, particularly in layers 1 and 2, compared to layer 5 (p-values 0.007 and 0.035, respectively) (Fig 3F). Fibers in layers 1 and 6 showed a preferential horizontal orientation parallel to the pial surface, while fibers in other cortical layers were oriented in all spatial orientations, with a notable presence of radial orientations. Fibers with varicosities were also found in all cortical layers, and their density was generally higher in superficial layers compared to the infragranular layers (Fig 3F). However, a statistically significant difference was found only between layers 1 and 4 (p-value: 0.048).

### Comparison of the three ascending NM systems

The present observations indicate that, in terms of fiber length per unit volume, the cholinergic system constitutes the densest NM system of the three systems studied, followed by the catecholaminergic fiber system, with the serotonergic system showing the lowest density of fibers. These differences were significant when averaging data from all cortical layers and also in each cortical layer separately (Fig 3). Regarding fiber varicosities, that represent the presumed sites of transmitter release, the density of cholinergic varicosities corresponded to 1.4 times higher than that of catecholaminergic varicosities and 2.3 times higher than that of serotonin axon varicosities. Finally, the density of catecholaminergic varicosities was 1.6 higher than that of serotonergic varicosities.

### *In silico* predictions about the organization of NM input

**Fig 4:**
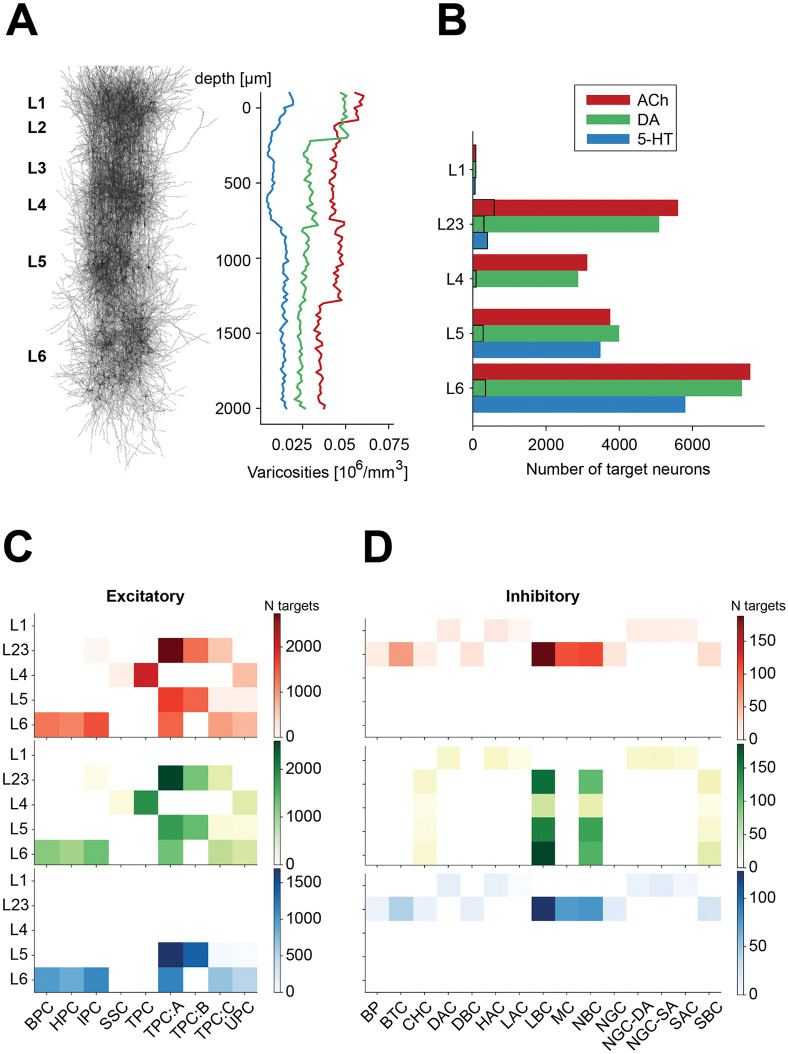
*In silico* predictions of ChAT, 5-HT and TH. All data is representative of a single column in our *in silico* neocortical microcircuit. **A)** From left to right: *in silico* Golgi stain microphotograph of a cortical column; predicted densities of NM varicosities *in silico*. **B)** Histograms showing the distribution of the number of postsynaptic targets contacted by each NM fiber, for the three NM systems. **C)** Heatmap of the proportion of excitatory morphological types contacted by each projection system. Excitatory cell m-types: BPC: Bipolar Pyramidal Cells (PCs); HPC: Horizontal PC; IPC: Inverted PC; SSC: Spiny Stellate Cell. TPC:A: Tufted PC, late bifurcation; TPC:B: Tufted PC, early bifurcation; TPC:C: Tufted PC, small tuft; UPC: Untufted PC. **D)** Heatmap of the proportion of inhibitory morphological types contacted by each projection system. Inhibitory m-types: BP: Bipolar Cell; BTC: Bitufted Cell; CHC: Chandelier Cell; DAC: Descending Axon Cell; DBC: Double Bouquet Cell; HAC: Horizontal Axon Cell; LAC: Large Axon Cell; LBC: Large Basket Cell; MC: Martinotti Cell; NBC: Nest Basket Cell; NGC: Neurogliaform Cell; NGC-DA: Neurogliaform Cell with dense axon; NGC-SA: Neurogliaform Cell with sparse axon; SAC: Small Axon Cell; SBC: Small Basket Cell.

To better characterize the neocortical neuropil, including NM inputs, we performed an *in silico* analysis of the distribution and organization of NM fibers. This enabled us to predict missing biological details, such as the relative proportions of targeted neurons and the number of contacts each NM fiber establishes, thereby filling gaps in the literature. We implemented two complete sets of NM projections, each tailored to work exclusively with synaptic transmission (ST) and volume transmission (VT) effects, respectively, for each of the three mentioned neuromodulators, resulting in a total of six sets of NM projections. To extend our assessment of the influence of NM systems we used the varicosity density profiles across the six neocortical layers to digitally reconstruct cholinergic, dopaminergic and serotonergic inputs to the hindlimb region of the rat somatosensory cortex and to obtain quantitative anatomical predictions about the columnar targets of the three NM innervation systems. Specifically, we calculate the total number of neurons innervated by each projection system, the number of postsynaptic cells contacted by each fiber and the most contacted cell types (Fig 4). To facilitate comparison across neuromodulatory systems and transmission modes, key in silico predictions of fiber targeting and connectivity are summarized in Table 4. These estimates are derived from experimentally measured layer-wise varicosity densities combined with the spatial distribution and dendritic geometry of reconstructed neuronal morphologies, rather than from EM-based cell-type-specific targeting probabilities. The number of neuromodulatory contacts received by a neuron is therefore determined by its dendritic geometry (and overall dendritic length) and laminar position. Consequently, neurons with larger dendritic arbors spanning layers with higher varicosity density receive proportionally more synaptic contacts. TPC and LBC have the largest total dendritic lengths in the circuit and are therefore the most heavily contacted by all three neuromodulatory systems. For a more refined breakdown of the proportions of cell-types innervated by the three systems see panel C in Fig 4. Next, we tested whether our virtual NM projection systems can be activated to induce network effects reported in the literature. We reasoned that albeit sparse, if properly connected and parameterized, NM fibers should elicit a modulation of network activity.

**Table 4:** In silico predictions of neuromodulatory fiber targeting and connectivity.

NM	Mode	Neurons targeted per fiber	Synaptic RS per fiber	Fibers received per neuron	Synaptic RS per neuron	Most contacted EXC	Most contacted INH
**ACh**	**ST**	**301 ± 494**	**337 ± 567**	**4.2 ± 2.7**	**4.7 ± 3.3**	**L23 TPC:A**	**L23 LBC**
**ACh**	**VT**	**7,105 ± 4,581**	**—**	**—**	**—**	**L23 TPC:A**	**L23 LBC**
**DA**	**ST**	**521 ± 852**	**631 ± 1,063**	**2.7 ± 1.6**	**3.3 ± 2.3**	**L23 TPC:A**	**L6 LBC**
**DA**	**VT**	**4,378 ± 3,983**	**—**	**—**	**—**	**L23 TPC:A**	**L6 LBC**
**5-HT**	**ST**	**366 ± 567**	**458 ± 740**	**2.0 ± 1.1**	**2.5 ± 1.7**	**L5 TPC:A**	**L23 LBC**
**5-HT**	**VT**	**3,463 ± 2,605**	**—**	**—**	**—**	**L5 TPC:A**	**L23 LBC**

Values are reported as mean ± SD. VT targets represent the number of neurons affected within the sphere of influence of a single fiber; synaptic RS are not defined for VT.

### Simulating cholinergic network effects

**Fig 5:**
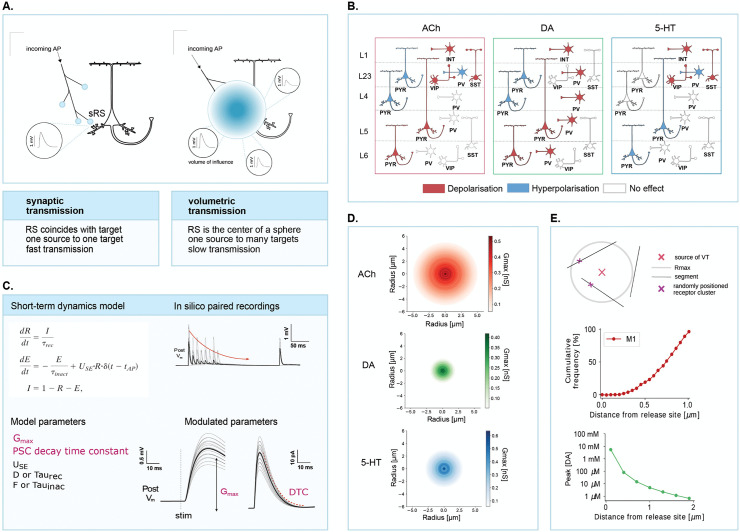
VT vs ST implementation. **A)** Schematics illustrating the differential implementation of volume vs synaptic transmission. Abbreviations: AP, action potential; sRS, synaptic release site; vRS, volume release site. **B)** Schematics illustrating the data gathered in Tables 5–7 on the effects of neuromodulator release on a given target that was used to parametrize the NM synaptic inputs to neocortical cells. The schema represents the main cell types in the neocortex. PYR, pyramidal cell; INT, interneuron; SST, somatostatin-positive cells; PV, parvalbumin-positive cells; VIP, vasoactive intestinal polypeptide cells. Note that SST, PV and VIP are classes of interneurons that group together multiple m-types, namely PV = CHC, LBC, and NBC; VIP = BP, NGC, and SBC; SST = MC; VIP and SST = BTC and DBC. **C)** Implementation of the synaptic model. Left: equations describing the Tsodyks-Markram model of synaptic short-term dynamics (above) and list of model parameters (below). Right: example of an *in silico* paired-recording experiment (excitatory, depressing connection) (above), and schematics illustrating the parameters that are modulated in order to mimic NM effects. G_max:_ maximal conductance; DTC: decay time constant of the postsynaptic current; U_SE_, utilization of synaptic efficacy; D or τ_rec_, time constant of depression (or recovery); F or τ_inact_, time constant of facilitation (or inactivation); NRRP, number of readily-releasable pool vesicles. **D)** Implementation of the volume transmission model for the three NM systems. Graphs illustrating the distance-dependent change in G_max_. **E)** First, from above: schematics illustrating the spherical sampling portion of the algorithm developed to model volume transmission (VT). Second, cumulative frequency plot showing the distance from the center of the nearest cholinergic terminals or varicosities to M1 muscarinic receptor, replotted from [16]. The curve represents the cumulative distribution (i.e., integral of the probability density distribution) of receptor-associated signal as a function of distance from release sites. Third, supporting evidence of the VT model for DA release (data replotted from [72]).

**Fig 6:**
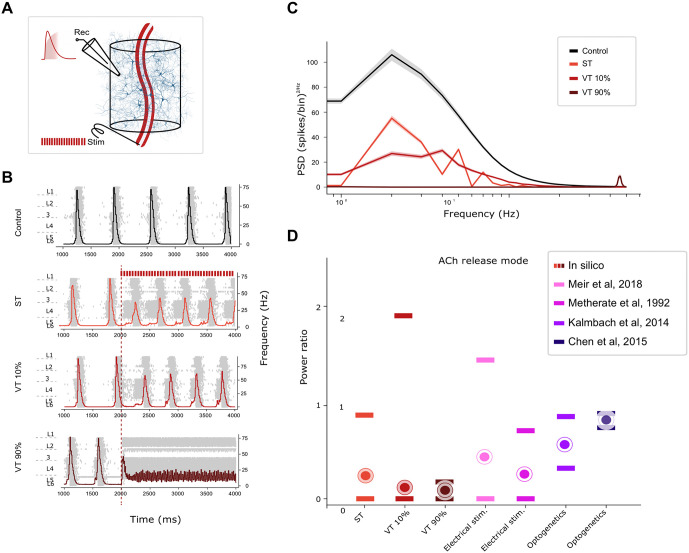
Network effects of volume or synaptic ACh release. Simulated network effects during the progressive activation of the virtual cholinergic projection systems. Timing of simulated optogenetic ACh release is shown as colored vertical bars on top of the first plot. Simulation time is 4000 ms, and projection activation occurs at t = 2000 ms and stops at t = 4000 ms. **A)** Schematics illustrating the simulation protocol **B)** Cholinergic effects; raster plots and superimposed frequency histograms. ST: synaptic transmission; VT: volume transmission. **C)** Power-frequency plots of the different simulated conditions. **D)** Delta-band (1.5–3 Hz) power for simulated conditions, expressed as the ratio relative to baseline. Baseline corresponds to the control condition shown in the upper portion of B. Dots indicate the mean and lines denote the SEM.

Optogenetic activation of ChAT-positive neurons in the basal forebrain (BF) is known to produce a desynchronizing effect on the activity of neurons in sensory cortices [64,65]. We gathered and integrated data about the effects of optogenetic cholinergic BF neurons stimulation on neocortical cell types residing in sensory cortices, to simulate the activation of cholinergic projections in our detailed model of a neocortical column. As reported in **Table 5** and **Fig 5**, in our implementation, cholinergic inputs depolarize L1 interneurons, L23 distal-targeting interneurons (BPs, BTCs, DBCs, SBCs, MCs, and NGCs) and L5 - L6 pyramidal cells (PCs), and instead have a hyperpolarizing effect on L23 - L4 PCs and L23 proximal-targeting interneurons (CHCs, LBCs, and NBCs). The remaining cell types are not targeted by our ACh connections because cholinergic stimuli in a physiologically relevant range (achieved via optogenetic tools or relatively low concentrations of ACh agonists, i.e., not greater than 100 µm), fail to elicit a response in inhibitory interneurons located in deeper layers [6,55]. For a more detailed explanation of the parameters used for simulations, we redirect the reader to **Table 2**. We simulated the cholinergic modulation of network activity as if it were: 1) completely synaptic and 2) wholly mediated by volume transmission. All simulations implementing the VT model were assigned a DTC of 608.6 ± 109.7 ms [18] while for the simulations implementing the ST model, we used DTC = 241.2 ± 15.5 ms [9]. First, we simulated the all-synaptic activation of the whole cholinergic projection system by approximating the firing frequencies used in optogenetics experiments (~ 20 Hz) which in turn imitated the synchronous spiking of cholinergic neurons in the BF [69]. In our simulations cholinergic ST inputs led to a prolonged desynchronization of network activity and brought about a significant decrease of the delta band (1.5-3 Hz) power (i.e., we observed a 68% reduction with respect to control; p-value = 6.07 ⋅ 10^-36^) (**Fig 6**). We then proceeded to simulate the progressive volume release of ACh by recruiting ascending fibers at incrementally higher levels, spanning from a minimal engagement (low levels of VT) to a full-scale involvement (high levels of VT), using a R_max_ = 5 µm. We observed that the desynchronization is even more powerful than in the ST case and report a reduction of the delta power of 78% (p-value = 2.46 ⋅ 10^-38^) when 10% of the ascending fibers are recruited and a drastic cessation of oscillatory activity at 90% fibers recruited (99% reduction; p-value = 1.85 ⋅ 10^-42^) (**Fig 6**). Network desynchronization depends on the number of neuromodulatory fibers recruited during stimulation. Recruiting larger fractions of the presynaptic population distributes neuromodulatory release across many independent spike trains, reducing temporal correlations and increasing spatial coverage of the circuit. Within the biologically plausible ranges modeled here, increasing fiber recruitment progressively strengthens desynchronization, providing a mechanism for graded neuromodulatory control of cortical states (**S2 Fig**).

**Table 5:** Summarized literature reported cell-type specific effects of cholinergic release. Inclusion criteria prioritized studies performed using optogenetic stimulation of subcortical nuclei to evoke endogenous neuromodulator release. When such data were not available, we considered studies using electrical stimulation of subcortical nuclei to evoke release, or exogenous bath application of neuromodulators.

ChAT	Pyramidal cells	Interneurons	Reference
**Layer 1**		Depolarization (and spiking activity)	[55] (p20-40 mice SSC) optogenetics
		Net depolarizing current	[18] (p25 mice); [66] (adult mice); optogenetics
**Layer 2/3**	Net hyperpolarizing current	Depolarization of BPCs	[55] (p20-40 mice SSC) optogenetics
		SOM increase FF; PV reduced FF	[67] (mouse v1) optogenetics
**Layer 4**	Hyperpolarization		[68] (adult mice SSC) optogenetics
**Layer 5**	Hyperpolarization; Depolarization		[69] (mouse V1) optogenetics
	Hyperpolarization; Depolarization		[70] (p24 mouse A1) optogenetics
**Layer 6**	Net depolarizing current		[18] (p25 mice); [71] (mouse PFC) optogenetics

**Table 6:** Summarized literature reported cell-type specific effects of dopaminergic release Inclusion criteria prioritized studies performed using optogenetic stimulation of subcortical nuclei to evoke endogenous neuromodulator release. When such data were not available, we considered studies using electrical stimulation of subcortical nuclei to evoke release, or exogenous bath application of neuromodulators.

TH	Pyramidal cells	Interneurons	Reference
**Layer 1**		Depolarization	[73] (adult rat PFC) Bath application of DA (30–100 μM)
**Layer 2/3**	Hyperpolarization; Depolarization	Increase excitability of PV-FS	[74] (3w rats PFC) in low Ca; [73] (adult rat PFC) Bath application of DA (30–100 μM)
		Increase excitability of FS and depolarization	[75] (rat PFC) 40 μM DA
**Layer 4**	Hyperpolarization; Depolarization	Increase excitability of FS and depolarization	[75] (rat PFC) 40 μM DA; [73] (adult rat PFC)
**Layer 5**	increase excitability via D1r (or sometimes decrease via D2r)	Increase excitability of PV-FS	[76] (rodents PFC); [74] (3w rats PFC); [77] (ferret PFC)
		Increase excitability of FS and depolarization;	[75] (40 μM DA, rat PFC); [73] (adult rat PFC)
**Layer 6**	Increase excitability		[76] (rodents PFC); [73] (adult rat PFC)

**Table 7:** Summarized literature reported cell-type specific effects of serotonergic release Inclusion criteria prioritized studies performed using optogenetic stimulation of subcortical nuclei to evoke endogenous neuromodulator release. When such data were not available, we considered studies using electrical stimulation of subcortical nuclei to evoke release, or exogenous bath application of neuromodulators.

5-HT	Pyramidal cells	Interneurons	Reference
**Layer 1**		5-HT2A/Cr-mediated excitatory responses	[78] (immature rat SSC)
		Slow-spiking interneurons are excited by 5-HT3Ar	[79] (juvenile SSC)
**Layer 2/3**		SOM neurons are excited via 5-HT2Ar	[80] (C57BL6 mice (6–10 weeks), olfactory cortex)
		Predominance of 5-HT1Ar-mediated inhibitions in FS neurons	[81] DR electrical stimulation
		PV neurons are excited by 5-HT	[82, 24]
		5-HT2A/Cr-mediated excitatory responses	[78] (immature rat SSC)
**Layer 5**	5-HT1A-mediated inhibitory responses in 2/3 of pyramidal neurons		[83] (rat mPFC, young adult)
	Inhibited by 5-HT application		[84] (3-weeks to 8-months-old C57BL6 mice PFC)

### Simulating dopaminergic network effects

**Fig 7:**
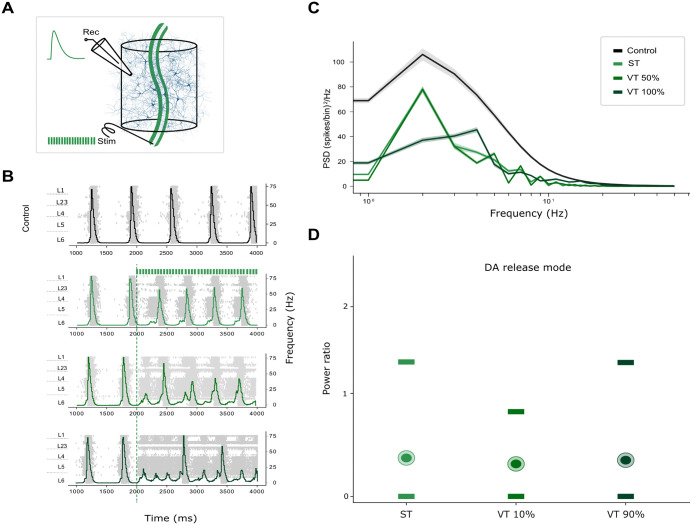
DA network effects. Simulated network effects during the progressive activation of the virtual dopaminergic projection systems. Timing of simulated optogenetic DA release is shown as colored vertical bars on top of the first plot. Simulation time is 4000 ms, and projection activation occurs at t = 2000 ms and stops at t = 4000 ms. **A)** Schematics illustrating the simulation protocol **B)** Dopaminergic effects; raster plots and superimposed frequency histograms. ST, synaptic transmission; VT, volume transmission. **C)** Power-frequency plots of the different simulated conditions. **D)** Delta-band (1.5–3 Hz) power for simulated conditions, expressed as the ratio relative to baseline. Baseline corresponds to the control condition shown in the upper portion of **B.** Dots indicate the mean and lines denote the SEM.

DA is known to modulate arousal and promote wakefulness in living animals [85], and to have effects on neocortical cell types [23,75,86]. However, ventral tegmental area inputs to sensory cortices have not been extensively described (as opposed to inputs to the prefrontal cortex), nor has their impact on network activity (unlike behavioral effects). For lack of better options, we used data about the effects on neocortical cell types elicited by bath application of dopaminergic agonists, mostly obtained in the prefrontal cortex, to parametrize the newly added dopaminergic synapses. As reported in **Table 6** and **Fig 5**, in our model, dopaminergic inputs depolarize L1 interneurons, L23 to L6 CHCs, LBCs, NBCs, and SBCs, and pyramidal cells in L5 and L6. Pyramidal cells in L23 and L4 are instead inhibited by DA. We chose not to target the remaining cell types because of lack of literature-reported effects. We implemented both the all-synaptic (ST) and the all-volume (VT) models of dopaminergic release, for which we used a R_max_ = 2 µm. The DTCs values were constrained through literature-reported values ST: 220 ± 41 ms [50]; VT: 0.4 ± 0.1 s [72]. First, we simulated the all-synaptic activation of the dopaminergic projection system to model increasingly higher levels of dopamine release. As shown in **Fig 7** dopaminergic ST inputs significantly reduce the delta component of the power spectrum of network activity (~49% reduction, p-value = 3.37 ⋅ 10^-32^). For all simulations of dopaminergic inputs, we chose a firing frequency of 20 Hz, again, to be aligned with optogenetic stimulation experiments [85,87]. Then we simulated an all-volume activation of dopaminergic projections and we found that VT transmission has a higher impact on network synchrony than ST: the delta power is reduced by 50% when 10% of the DA fibers are stimulated (p-value = 1.1 ⋅ 10^-32^) and we observe a 68% reduction when 90% of the DA fibers are stimulated (p-value = 9.1 ⋅ 10^-38^).

### Simulating serotonergic network effects

**Fig 8:**
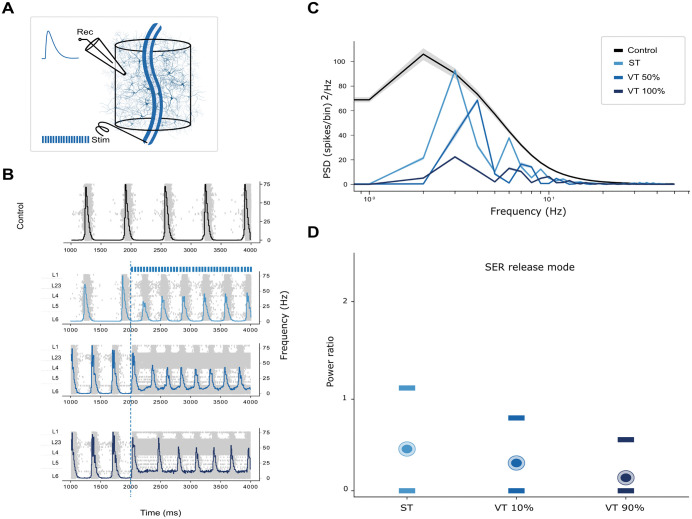
5-HT network effects. Simulated network effects during the progressive activation of the virtual serotonergic projection systems. Timing of simulated optogenetic 5-HT release is shown as colored vertical bars on top of the first plot. Simulation time is 4000 ms, and projection activation occurs at t = 2000 ms and stops at t = 4000 ms. **A)** Schematics illustrating the simulation protocol **B)** Serotonergic effects; raster plots and superimposed frequency histograms. ST: synaptic transmission; VT: volume transmission. **C)** Power-frequency plots of the different simulated conditions. **D)** Delta-band (1.5–3 Hz) power for simulated conditions, expressed as the ratio relative to baseline. Baseline corresponds to the control condition shown in the upper portion of **B.** Dots indicate the mean and lines denote the SEM.

A substantial body of evidence suggests a causal relationship between 5-HT levels and cortical activity [24,64,88,89]; optogenetic stimulation of the DR reduces low frequency power in the cortex thus promoting desynchronization. However, these results are mostly obtained in prefrontal areas, and the role of 5-HT projections to the SSCx is not clear to date. To test whether this holds true for the somatosensory cortex as well, we parameterized the serotonergic connections as reported in experiments where 5-HT was bath-applied (see **Table 7**). As reported in **Fig 5**, serotonergic inputs hyperpolarize lower layers (L4 and L5) pyramidal cells and L23 PV interneurons but have excitatory effects on L23 VIP and SST interneurons, and (as for most neuromodulators) they depolarize all L1 interneurons. We chose not to target the remaining cell-types because of lack of literature-reported effects. For a more detailed explanation of the parameters used for simulations, we redirect the reader to the **Methods** section of this paper and to **Table 2**. We implemented both the all-synaptic (ST) and the all-volume (VT) model of 5-HT release, for which we used a R_max_ = 3 µm. The DTCs values were constrained through literature-reported values (we assigned ST and VT the same value for lack of better data: 0.44 ± 0.03 s [22]). For all simulations of 5-HT release we used a train of pulses at 20 Hz (to fall in line with optogenetics experiments such as the study performed by [90]). First, we simulated the all-synaptic activation of 5-HT fibers to check the effect on slow oscillations. We found that ST 5-HT inputs reduce the delta component of network oscillations (~87% reduction, p-value = 7.5 ⋅ 10^-41^) and shift the power spectrum towards higher-frequency components (5–8 Hz or the theta range) as shown in the raster plots in **Fig 8**. Simulating the all-volume 5-HT release also impacted delta oscillations, with the effect’s magnitude correlating with the number of stimulated fibers (at 10% fibers recruited: 99%, p-value = 2.02 ⋅ 10^-42^ and 90% fibers recruited: delta power reduction of 97%, p-value = 4.3 ⋅ 10^-42^). Furthermore, volume 5-HT release also induces oscillations in the lower theta range, as shown in the raster plots in **Fig 8**.

## Discussion

### Anatomy of NM systems

In the present study we have estimated densities and laminar distribution patterns of NM systems in the rat P14 HLS1 using immunostaining and stereological techniques. These methods enable the identification and quantification of virtually all cortical synapses within a long series of images that represent a 3D sample of cortical neuropil [91,92]. Previous studies in different cortical regions and species have examined the developmental maturation of the rat cortical innervation by the networks of cholinergic, catecholaminergic and serotonergic fibers [13,19,63,93–97]. These studies suggested that the pronounced effects of these neuromodulatory systems on the morphology and physiology of maturing cortical neurons are mediated through both synaptic and non-synaptic connections. They reported regional specificity throughout the neocortex and progressive changes over postnatal development in fiber length, branching patterns, number of varicosities and percentage of varicosities forming synaptic contacts. To gain deeper insight into the organization of the cortical neuropil across different layers of the HLS1 in two-week old rats, we have also estimated the fiber length per cortical volume and the density of varicosities of catecholaminergic, serotonergic and cholinergic systems, using immunocytochemical and stereological techniques.

After the cholinergic system, the most prevalent type of NM innervation in the cortex is the TH-positive fiber system. TH-positive, putative dopaminergic fibers, are generally thought to be predominantly located in the frontal areas [98] and are thought to sparsely innervate the sensory cortices [47]. Our findings confirm previous observations of TH staining in the developing somatosensory cortex [27]. Anatomical studies in various rodent species have identified substantial serotonergic projections from the raphe nuclei to early sensory areas, including the somatosensory cortex [98]. 5-HT fiber density transiently increases in the neonatal rodent neocortex [26] and becomes more uniformly distributed after the third postnatal week. In our study, we confirm that serotonergic fibers innervate the developing rodent neocortex and are more abundantly present in superficial rather than deep layers. This preferential organization of serotonergic fibers and varicosities may be related to the concentration of a large, developmentally distinct category of inhibitory interneurons that express the 5-HT3a receptor, which is predominantly found in the supragranular layers. This population is heterogeneous and includes all VIP expressing neurons as well as numerous subgroups of neurons that do not express VIP, including neurogliaform cells [99]. The preferential innervation of layer 1 seems to be a key feature of NM projections to the somatosensory cortex; given the relative scarcity of cell bodies in layer 1, it is reasonable to hypothesize that they would be contacted by a large number of fibers, in order to maximize the probability of receptor activation. To gain a better insight of the organization of NM systems, we also developed a structural model of cholinergic, dopaminergic and serotonergic innervation of the sensory cortex. Our goal here was to generate predictions for quantities that are challenging to measure directly, such as the number of neurons contacted by each NM axon, the number of synapses established by each fiber and the proportions of innervated cell types. Our combined experimental and modeling approach demonstrates that the cholinergic projection system prevails over other NM systems in the cerebral cortex and contacts by far the largest number of postsynaptic targets across all neocortical layers.

### Physiology of NM systems

To investigate the chemical modulation of sensory cortices we implemented a model of NM release that accounts for volume and synaptic transmission in a detailed, biologically accurate model of the somatosensory cortical column [30,33]. While there exists a lot of data for the neuromodulator ACh, which allows us to build a model with fairly precise information about the cholinergic system, the roles of DA and 5-HT in sensory areas of the neocortex are less understood, as most studies have focused on their effects in prefrontal regions, leaving gaps in our understanding of their functions in regions like the somatosensory cortex. Therefore we used much sparser datasets to constrain our models of dopaminergic and serotonergic release. To this date we are not familiar with any literature reporting the network effects of DA and 5-HT in somatosensory circuitry which admittedly makes it harder to validate the results of our model. In this study, we aimed to comprehensively investigate the three fiber systems, focusing on both anatomical and functional aspects. While we had previously conducted an in-depth review of the ACh system [1], our current work provides a quantitative assessment of cholinergic, dopaminergic and serotonergic inputs to the sensory cortex. The complexity and intricacy of these systems pose significant challenges for direct measurement. Therefore, we adopted a modeling approach that leverages available data to construct a comprehensive framework. The simulations generated testable predictions that offer preliminary insights into possible mechanisms of neuromodulator action in the cortex, contributing to the broader effort to understand their roles in cortical dynamics.

The neuromodulatory effects modeled in this study are implemented at a phenomenological, conductance-based level and are not intended to represent explicit molecular or intracellular signaling pathways. In particular, we do not model receptor subtype–specific cascades or direct modulation of intrinsic ionic channels, e.g., muscarinic suppression of the M-current [100] or presynaptic inhibition of glutamatergic or GABAergic transmission [101,102,103]. Although these mechanisms are well established experimentally, quantitative, cell-type- and layer-specific constraints required to parameterize such effects in a large-scale biophysical network model are currently lacking, particularly in developing sensory cortex. Instead, neuromodulator release activates added conductances that reproduce the experimentally observed net depolarizing or hyperpolarizing influence on neuronal excitability with defined kinetics and spatial organization. This abstraction allows us to study how anatomically grounded neuromodulatory input shapes network-level dynamics while maintaining compatibility with the underlying Hodgkin–Huxley neuron models. Incorporating receptor-specific intracellular mechanisms and presynaptic modulation represents an important direction for future work as more quantitative data become available.

Furthermore, in this work, we use the term physiologically relevant to denote cholinergic manipulations that approximate endogenous cortical acetylcholine signaling, either through optogenetic activation of basal forebrain inputs or through relatively low concentrations of cholinergic agonists, typically not exceeding ~100 µM. Our focus on this literature was not intended to imply that such in vivo studies provide direct measurements of membrane conductances, as most do not include intracellular voltage- or conductance-clamp recordings. Rather, these experiments define the temporal profile, magnitude, and circuit context of cholinergic modulation under conditions closer to those occurring in vivo. In addition, as summarized in Tables 5–7, they provide a consistent indication of the net direction of modulation (depolarizing vs. hyperpolarizing) across cell types, which we use to constrain the sign of cholinergic effects in our modeling. The explicit membrane conductances required for biologically detailed computational models are primarily derived from intracellular and voltage-clamp studies in slices using bath or focal application of cholinergic agonists. In this sense, in vivo optogenetic studies serve as an intermediate link, constraining the physiological regime within which conductance-based mechanisms identified in vitro are likely to operate.

It is important to highlight that our simulation of the modulation induced by ACh involves fewer release sites than those quantified experimentally and incorporated into our structural model. This discrepancy may be attributed to certain cell types being unresponsive to ACh connections within a physiologically relevant range, as documented in existing literature [6,55]. Consequently, while these release sites are present at the structural level in our model, they remain functionally silent. In our simulations sparsely distributed cholinergic projections significantly modulate the activity of the microcircuit by decreasing the power of slow oscillations (in the delta range) and by desynchronizing network activity. Notably, our findings indicate that elevated levels of volume transmission lead to a complete and enduring desynchronization of the microcircuit, a result inconsistent with previously reported literature. Conversely, the observed modulation aligns more closely with the effects induced by cholinergic synaptic transmission or low levels of volume transmission, as illustrated in **Fig 6, panel B** [5,67,68,104]. Hence, we believe that the traditional perspective of NM transmission having profound and widespread effects on cortical regions through simultaneous activation of all fibers originating from one nucleus, should be reconsidered. While in the hippocampal CA1 region cholinergic VT is supported by experimental evidence [12], it is reasonable to hypothesize instead that cortical NM activity reflects a complex interplay of spatiotemporal dynamics [105] influenced by a multitude of factors. These factors may include the number of fibers involved, axonal firing frequency, density of receptors at the receiving end, as well as catalytic enzymatic activity, among others. No amount of validation can conclusively prove a model right, but within its domain of validity, our model facilitated the rigorous testing of the underlying hypothesis, which is the data used to constrain the model. More specifically, we demonstrate that conflicting results regarding cell-type specific effects of cholinergic release can be reconciled through *in silico* experiments conducted within our framework. The literature about the cell type specific cholinergic modulation of membrane properties is quite inconsistent. For example, there is no clear agreement on the effects exerted by ACh on pyramidal neurons, nor on basket cells (PV-FS interneurons). Some papers reported inhibitory effects, others excitatory or biphasic, or (sometimes paradoxically) a lack of effect [6,69,70,106,107,108]. The same is true for measurements of transmission features such as the DTC, or the firing frequency of cholinergic cells of subcortical provenance. Because our model replicates well-established emergent phenomena such as the desynchronizing effect of cholinergic release reported in the literature [65,109], we can be confident that it captures at least some essential properties of the system being modeled. We simulated the effects of endogenous DA release, revealing that ST alone leads to a significant decrease (30%) in the delta power of microcircuit oscillatory activity. However, VT exerts an even more pronounced impact on slow oscillations. Notably, it is intriguing to observe that the desynchronization induced by DA is not as enduring as observed in the ACh case, despite equivalent train stimulation, and the effect is not as robust. This discrepancy may be attributed to the smaller radius, lower density of release sites, and potential variations in wiring properties of the connections within the DA system. Through our simulations of 5-HT release, we consistently observed a pronounced influence on delta oscillations both via ST and VT, with the magnitude of this effect directly tied to the number of stimulated fibers. Notably, our findings revealed a unique feature associated with 5-HT release: the generation of oscillations within a specific frequency range, particularly in the lower theta range. This distinctive pattern was not observed in simulations involving other neuromodulators and might be attributed to the modulation of specific targeted cell types. The emergence of oscillatory activity in the theta frequency range following serotonergic modulation can be interpreted as a consequence of a shift in the excitation–inhibition balance toward inhibition. In our model, 5-HT primarily hyperpolarizes deep-layer pyramidal neurons (L5–L6) while depolarizing interneurons in superficial layers (L1–L2/3), based on experimentally reported cell-type-specific effects. This differential modulation suppresses slow delta oscillations while promoting rhythmic, inhibition-paced activity, in which increased excitability of superficial interneurons can entrain network dynamics at higher frequencies. Unlike ACh and DA, which predominantly reduce inhibitory tone and drive strong desynchronization, serotonergic modulation preserves structured oscillatory activity by maintaining a balance between reduced excitation and enhanced inhibition. We emphasize that the resulting theta oscillations should be interpreted as model-derived predictions emerging from the integration of available anatomical and physiological constraints, highlighting a potential mechanism by which serotonergic inputs may differentially regulate cortical network states in sensory cortex. In essence, our findings demonstrate that all three NM systems exert a desynchronizing effect on the circuit, with ACh exhibiting a significantly greater impact compared to DA and 5-HT. Notably, the presence of faster oscillations induced by 5-HT suggests distinct roles for each neuromodulator. Furthermore, our simulations reveal marked differences between synaptic transmission (ST) and volumetric transmission (VT) in their ability to desynchronise network activity. We find that VT exerts a much stronger effect, largely attributable to its broader spatial footprint. As shown in **Fig 5A**, VT engages many more dendritic targets than ST, resulting in a substantially larger overall conductance. Accordingly, **Fig 6B** demonstrates that recruiting only a small fraction of VT fibers produces a network effect comparable to full recruitment of ST fibers, suggesting that the enhanced desynchronisation does not arise from preferential targeting of distinct neurons but from diffuse, cumulative innervation.

In addition to these spatial differences, VT is characterised by slower transmission kinetics, which likely promote temporal summation of conductance changes and further amplify its desynchronising effect. This mechanism can readily drive the network into a regime of excessive desynchronisation: increasing VT recruitment produces a reduction in delta power that exceeds experimental observations. Consistent with this, the optogenetically observed reduction in delta power is more closely matched by the ST condition, at least for cholinergic modulation. (**Fig 6D**). Together, these findings suggest that the balance between spatial extent and temporal dynamics critically determines the strength of desynchronisation.

### Modeling assumptions

In the process of modeling, simplifications are inherently made from the ground truth. These simplifications rely on assumptions, and as long as these assumptions remain valid, the corresponding simplification does not limit the validity of the model. Consequently, in order to assess the validity of a model it is paramount to be aware of the assumptions it is based on. In the next paragraph we will describe three main types of assumptions that our model is based upon. The first type is the “inherited assumption”, that occurs whenever a model is built on top of another existing model. As explained in **Table 8**, the new model inherits all relevant assumptions of the base model. For example, our algorithm to reconstruct NM innervation inherits the assumptions relating to neuronal composition, placements and morphology from the underlying neocortical column model. The second type is the data/structuring assumption: that is, an assumption that acts on how the model is parameterized, i.e., how data is turned into parameters. In this modeling study, after placing additional synapses in our NCX model to match the distribution profile observed experimentally, we assigned synaptic features based on literature reports gathered in Tables 5–7. We developed an algorithm that assigns excitatory or inhibitory synaptic parameters based on the postsynaptic morphological type (m-type) contacted by our virtual projection systems. We assumed that synapses of subcortical provenance will display forms of short-term dynamics similar to the canonical short-term synaptic dynamics of neocortical synapses, as synthesized in Markram et al. [30] and based on extensive primary electrophysiological studies, and that they would be governed by similar principles. Moreover, we assume that organizational principles extracted for specific cell types hold true in more general cases and we apply them to broader target classes. For example, if we find that ACh depolarizes L5 pyramidal cells we extend this observation to a broader category, i.e., L5 excitatory cells. Additionally, we compute the number of fibers for each NM projection system based on rough estimations of the number of presynaptic cells in the underlying subcortical NM region. This assumption has limited validity in many ways. 1) We are assuming that NM projections originate from a specific nucleus in the subcortical modulatory region (in particular: the NBM for ACh, the dorsal raphe or DR for 5-HT and the VTA for DA) and we are disregarding the possibility that some proportion of the connections can arise from other less relevant or less studied nuclei. While it is true that these nuclei are the main sources of NM inputs, the possibility that other nuclei are also involved cannot be excluded. 2) We assume that the innervation of the S1 region is homogeneous in all its subregions, while there may be differences in the innervation of specific areas. The third type is the modeling assumptions, that is all the assumptions at the very core of the model itself. For instance, we assume that the slow oscillatory activity of our simulated microcircuit (yielded by a specific set of parameters) can mimic the inactivated/synchronized brain state typical of SWS states (see **Methods** section - **Microcircuit**). The other modeling assumptions revolve around the VT implementation. Information such as the concentration of the neurotransmitter in the extracellular matrix, and the dynamics of the release in terms of diffusion kinetics is central to understanding the spatiotemporal constraints of VT. However, neither the effective concentration of neuromodulators in the extracellular space, nor the rates of their diffusion/degradation are known and an accurate knowledge of the spatiotemporal limits of diffuse neuromodulator release is still lacking [2], mostly because of technological limitations. In our model, we assume that the extracellular matrix has no influence on volume transmission, and we do not specifically model the action of catalytic enzymes like cholinesterases. Additionally, we sample a spherical volume around the VT release site and assume transmission isotropy [112]. The theoretical goal of having a complete list of assumptions is unachievable, for the simple reason that the space of all conceivable models is most likely impossible to describe, but it provides a useful thought framework.

**Table 8:** List of assumptions. Classification of inherited, data/structuring, and modeling assumptions underlying the construction of the neuromodulatory circuit model. “Inherited assumptions” refer to constraints inherited from the base neocortical microcircuit model [30]. “Data/structuring assumptions” arise from how experimental measurements are translated into model parameters. “Modeling assumptions” refer to simplifications intrinsic to the implementation of synaptic and volume transmission.

Assumption	Type
Neuronal composition, placements and morphologies	**inherited assumption** from [30]
Synapses of subcortical provenance will display forms of short-term dynamics similar to the well-known dynamics of neocortical synapses	**data/structuring assumption** [110]
Organizational principles extracted for specific cell-types hold true in more general cases and we apply them to broader target classes	**data/structuring assumption** [30]
NM projections originate from a specific nucleus in the subcortical modulatory region	**data/structuring assumption** [44,45,47]
Innervation of the S1 region is homogeneous in all its subregions	**data/structuring assumption** [111]
Slow oscillatory activity of our simulated microcircuit can mimic the inactivated/synchronized brain state typical of slow wave sleep states (SWS)	**modeling assumption**
Extracellular matrix has no influence on volume transmission	**modeling assumption**
Sample a spherical volume around the VT release site and assume transmission isotropy.	**modeling assumption** [112]

### Model limitations

While our model provides valuable insights, it has limitations, some of which are detailed in the assumptions section.

Moreover, the model incorporates key biophysical properties and anatomically grounded neuromodulatory innervation patterns, but we acknowledge that the simulated network activity does not fully recapitulate the complexity of in vivo cortical dynamics. In particular, oscillatory patterns observed in the model differ in structure and spectral content from those typically recorded in behaving animals. This discrepancy limits the direct comparability of the simulated activity with in vivo data. However, the intent of the model is not to reproduce precise neural dynamics under specific behavioral conditions, but rather to provide a controlled framework for exploring how different neuromodulatory systems, individually and in combination, might influence network-level behavior. As such, the model serves primarily as a tool for hypothesis generation and mechanistic exploration.

The model focuses on postsynaptic neuromodulatory effects on cellular excitability and does not currently incorporate presynaptic modulation of glutamatergic or GABAergic synaptic transmission. The extensive literature documents muscarinic presynaptic inhibition of transmitter release [113,114], as well as presynaptic effects of dopamine and serotonin on synaptic efficacy [115,116]. These mechanisms likely contribute significantly to network-level effects of neuromodulation. However, implementing presynaptic modulation would require cell-type-specific, synapse-type-specific quantitative data on the magnitude and kinetics of modulation that are not currently available for the developing somatosensory cortex. Future iterations of this modeling framework could incorporate presynaptic effects as more comprehensive datasets become available from targeted experimental investigations.

Furthermore, our model does not account for the action of other neuromodulators in the cortex [117], which likely influence the context in which ACh, DA and 5-HT operate. Additionally, we did not address the complex interactions between these three neuromodulators or their regulatory mechanisms. For example, ACh plays a role in the feedback regulation of cholinergic release via presynaptic axonal autoreceptors, and neuromodulators can be co-released with other peptides or transmitters [118,119]. Future work could integrate these additional factors to provide a more comprehensive representation of NM dynamics in the somatosensory cortex.

Our phenomenological implementation of DA modulation can be contrasted with the mechanistic approach pioneered by [120,121] for prefrontal cortex neurons. Their model directly modified intrinsic Hodgkin-Huxley conductances based on extensive experimental characterization of D1 and D2 receptor effects on specific ionic channels (Na ⁺ , K ⁺ , Ca² ⁺ conductances). This mechanistic approach enabled investigation of how receptor-specific ionic channel modulation contributes to computational functions such as working memory maintenance and cognitive flexibility. We did not adopt this mechanistic approach for somatosensory cortex because comparable cell-type-specific characterization of dopamine receptor effects on ionic conductances is not available for somatosensory cortex, and the functional role of DA in sensory cortex may differ from its role in prefrontal cortex. A similar limitation applies to our implementation of cholinergic modulation, which is likewise modeled phenomenologically. In particular, our model does not explicitly simulate the modulation of specific ionic currents such as the M-current or afterhyperpolarization (AHP) currents, which have been characterized in detail in previous conductance-based models [122,123]. Instead, both dopaminergic and cholinergic effects are represented as net changes in excitability or synaptic efficacy, constrained by experimental observations. Nevertheless, our phenomenological conductances capture the net functional outcomes documented in the experimental literature. Future work could bridge these approaches by: (1) conducting systematic optogenetic activation studies of VTA, BF, and raphe inputs to somatosensory cortex combined with cell-type-specific recordings; (2) characterizing NM receptor subtype expression across cortical layers and cell types in developing somatosensory cortex; (3) measuring receptor-specific effects on individual ionic conductances (g_Na_, g_K_, g_Ca_, etc.) for different m-types; and (4) implementing mechanistic neuromodulation following the Durstewitz and Seamans framework once these data become available.

Despite these limitations, our framework serves as a foundation that can be refined and expanded as new data emerge and our understanding of NM systems deepens.

## Conclusions and future directions

In this study, we aimed to integrate sparse experimental datasets to explore the anatomy and physiology of three distinct neuromodulatory systems in the somatosensory cortex. Through our modeling efforts, we developed a framework for investigating the long-standing issue of volume versus point-to-point synaptic transmission. Future refinements, including more realistic input patterns, behavioral state modulation, and improved constraints from in vivo data, will be essential to increase the model’s fidelity and predictive power.

### Resource availability

#### Lead contact.

Further information and requests for data, methods and code should be directed to and will be fulfilled by the lead contacts: Cristina Colangelo (cristina.colangelo11@gmail.com) or Srikanth Ramaswamy (srikanth.ramaswamy@newcastle.ac.uk).

#### Materials availability.

No materials were used in this computational work.

#### Data and code availability.

Data on varicosity densities of neuromodulatory inputs are reported in figures and text in this publication and deposited at Zenodo (https://doi.org/10.5281/zenodo.14587678).

Volumetric atlases, neuron reconstructions, the parameterization of connectivity in JSON format, and the description of the model in SONATA format have been deposited at Zenodo and are publicly available as of the date of the publication.

Original code has been deposited at Zenodo and is publicly available at https://doi.org/10.5281/zenodo.14587678.

## Supporting information

S1 Fig ChAT and TH intracortical cell bodies. **A)** Confocal stack projection image, corresponding to a cortical thickness of 14 µm, showing DAPI staining in the different layers of the P14 rat hindlimb somatosensory cortex. **B)** Same as in A but showing the distribution of TH-immunoreactive fibers (green). Squared zones are shown at higher magnification in panel **C)** and panel **D)** where arrowheads point to cell bodies. **E)** Same as in B) but for ChAT immunoreactive fibers. Squared zones are shown at higher magnification in panel **F)** and panel **G)** where arrowheads point to cell bodies. Scale bar spans 15 µm.
(TIF)

S2 Fig Effects of ACh volumetric transmission. Simulated network effects during the progressive activation of the VT cholinergic projection system. Timing of simulated optogenetic ACh release is shown as colored vertical bars on top of the raster plots. Simulation time is 4000 ms, and projection activation occurs at t = 2000 ms and stops at t = 4000 ms.
(TIF)

## Abbreviations

PV: parvalbumin
SOM: somatostatin
FS: fast spiking
HLS1: hind limb region of the primary somatosensory cortex
P14: postnatal day 14
HPC: horizontal pyramidal cell
IPC: inverted pyramidal cell
SSC: spiny stellate cell
PC: pyramidal cell
TPC:A: tufted pyramidal cell type A
TPC:B: tufted pyramidal cell type B
TPC:C: tufted pyramidal cell type C
UPC: untufted pyramidal cell
LBC: large basket cell
NBC: nested basket cell
SBC: small basket cell
NGC: neurogliaform cell;
NGC-DA: neurogliaform cell descending axon
NGC-SA: neurogliaform cell small axon
MC: martinotti cell
BPC: bipolar cell
BTC: bitufted cell
CHC: chandelier cell
DAC: descending axon cell
LAC: large axon cell
HAC: horizontal axon cell
SAC: small axon cell
EPSP: excitatory postsynaptic potential
IPSP: inhibitory postsynaptic potential
EPSC: excitatory postsynaptic current
IPSC: inhibitory postsynaptic current
D1r: dopamine receptor 1
D2r: dopamine receptor 2
5-HT1Ar: serotonin receptor 1A
