# Supplementary material for: Quantitative anatomy and biophysical modeling of ascending neuromodulatory systems in the developing rat neocortex
Source: PLoS Comput Biol. 2026 Jun 26;22(6):e1014460. doi: 10.1371/journal.pcbi.1014460 (PMC13345462; doi:10.1371/journal.pcbi.1014460)
Supplement: Attachment — Submitted filename: Rebuttal PCOMPBIOL ACh.pdf [file pcbi.1014460.s005.pdf]

PCOMPBIOL-D-25-01586

Answers to Editor’s and Reviewers’ comments

Dear Dr. Antonietti,

Thank you for submitting your manuscript to PLOS Computational Biology. After careful consideration, we feel that it has merit but does not fully meet PLOS Computational Biology's publication criteria as it currently stands. Therefore, we invite you to submit a revised version of the manuscript that addresses the points raised during the review process.

Please submit your revised manuscript by Jan 12 2026 11:59PM. If you will need more time than this to complete your revisions, please reply to this message or contact the journal office at ploscompbiol@plos.org. When you're ready to submit your revision, log on to https://www.editorialmanager.com/pcompbiol/ and select the 'Submissions Needing Revision' folder to locate your manuscript file.

Please include the following items when submitting your revised manuscript:

* A rebuttal letter that responds to each point raised by the editor and reviewer(s). You should upload this letter as a separate file labeled 'Response to Reviewers'. This file does not need to include responses to formatting updates and technical items listed in the 'Journal Requirements' section below.
* A marked-up copy of your manuscript that highlights changes made to the original version. You should upload this as a separate file labeled 'Revised Manuscript with Track Changes'.
* An unmarked version of your revised paper without tracked changes. You should upload this as a separate file labeled 'Manuscript'.

If you would like to make changes to your financial disclosure, competing interests statement, or data availability statement, please make these updates within the submission form at the time of resubmission. Guidelines for resubmitting your figure files are available below the reviewer comments at the end of this letter

We look forward to receiving your revised manuscript.

Kind regards,

Roman Bauer
Academic Editor
PLOS Computational Biology

Andrea E. Martin
Section Editor
PLOS Computational Biology

Additional Editor Comments:

Dear Authors,
Thank you for submitting your manuscript entitled “Quantitative anatomy and biophysical modeling of ascending neuromodulatory systems in developing rat cortex” to PloS Computational Biology. Your paper has now been evaluated by three expert reviewers.
Two reviewers recommended minor revision, noting that the study is generally well executed and of potential interest. However, the third reviewer raised substantive conceptual concerns regarding the modelling and interpretation of your results.

Specifically, they commented that the manuscript would require a major restructuring to focus more clearly on the effects of neuromodulators on membrane conductances, rather than on the rather simplified concepts indicated in Tables 5–7.

We thank the editor and Reviewer 3 for raising this important point, which has allowed us to clarify a central aspect of our modeling approach.

Our model does not implement neuromodulatory effects by modifying intrinsic Hodgkin–Huxley membrane conductances (e.g., M-current, K(AHP), leak currents) of the underlying neuronal models described in *Markram et al. (2015)*. Instead, neuromodulation is implemented at a ***phenomenological level by adding intrinsic conductances*** that are regulated by synaptic or volume release of acetylcholine, dopamine, or serotonin. These added conductances represent the net effect on neuronal excitability and synaptic transmission, without explicitly modeling receptor subtypes or downstream ionic channel cascades.

Tables 5–7 (now Tables 6-8) were therefore intended to summarize experimentally observed functional effects on neuronal excitability (e.g., depolarization, hyperpolarization, changes in firing probability) that constrain the sign, magnitude, and kinetics of these added conductances—not to imply that neuromodulation is modeled as simple EPSCs or IPSCs in the classical synaptic sense. We agree that this distinction was not sufficiently explicit in the original manuscript.

To address this, we have revised the manuscript to explicitly state our modeling philosophy and level of abstraction in the Introduction and Methods. In addition, we have expanded the Discussion to acknowledge that well-established molecular mechanisms (e.g., muscarinic modulation of M-current, presynaptic inhibition) are not explicitly modeled here due to the lack of quantitative, cell-type-specific constraints, and are therefore treated as limitations and future directions.

In addition, there was some agreement with regards to unclarity regarding the use and interpretation of fiber length (density) in your analysis. Since this measure appears central to several of your conclusions, it will be important to clarify how it was defined, computed, and biologically interpreted.

We agree that the original terminology was insufficiently precise and may have caused confusion regarding the definition and interpretation of this measure. We have revised the manuscript to clearly express that what we measured and reported were between fiber length density, defined as the total length of immunoreactive fibers per unit volume of tissue (μm of fiber per μm^3), estimated using the space ball stereological probe. This measure is a three-dimensional density estimate that is independent of fiber orientation and is widely used to quantify axonal innervation in cortical tissue. The Methods section heading has been changed to “Estimation of fiber length density”, and the computation of this quantity is now described explicitly.

In terms of biological interpretation, fiber length density is used in this study as an anatomical descriptor of the abundance and laminar distribution of neuromodulatory axons, rather than as a direct proxy for synaptic connectivity or release probability. Importantly, while fiber length density provides complementary information about the overall organization of neuromodulatory projections, the placement of release sites in the computational model is constrained by varicosity density, not by fiber length density. We have clarified this distinction in both the Methods and Results sections to avoid ambiguity.

We believe that these clarifications resolve the previous ambiguity and make the role of fiber length density in the study explicit and biologically interpretable.

Given these and other points, the issues extend beyond minor editorial adjustments and call for a substantial revision to improve conceptual focus and methodological clarity.

Journal Requirements:

1) We ask that a **manuscript source file** is provided at Revision. Please upload your manuscript file as a .doc, .docx, .rtf or .tex. If you are providing a .tex file, please upload it under the item type u2018LaTeX Source Fileu2019 and leave your .pdf version as the item type u2018Manuscriptu2019.

We have provided the source file (docx).

2) Please provide an **Author Summary**. This should appear in your manuscript between the Abstract (if applicable) and the Introduction, and should be 150-200 words long. The aim should be to make your findings accessible to a wide audience that includes both scientists and non-scientists. Sample summaries can be found on our website under Submission Guidelines:
https://journals.plos.org/ploscompbiol/s/submission-guidelines#loc-parts-of-a-submission

We have added the Author Summary

3) Please **upload all main figures as separate Figure files in .tif or .eps format.** For more information about how to convert and format your figure files please see our guidelines:
https://journals.plos.org/ploscompbiol/s/figures

We have included all the Figures as .tif files.

4) We notice that your **supplementary Figures** are included in the manuscript file. Please remove them and upload them with the file type 'Supporting Information'. Please ensure that each Supporting Information file has a legend listed in the manuscript after the references list.

We have removed supplementary Figures and included legends after the bibliography.

5) Please amend your detailed **Financial Disclosure statement.** This is published with the article. It must therefore be completed in full sentences and contain the exact wording you wish to be published.
- State the initials, alongside each funding source, of each author to receive each grant. For example: "This work was supported by the National Institutes of Health (####### to AM; ###### to CJ) and the National Science Foundation (###### to AM)."
- **State what role the funders took in the study.** If the funders had no role in your study, please state: "The funders had no role in study design, data collection and analysis, decision to publish, or preparation of the manuscript.".
If you did not receive any funding for this study, please simply state: The authors received no specific funding for this work.

We have amended the Financial Disclosure Statement as requested.

Reviewers' comments:

Reviewer's Responses to Questions

Comments to the Authors:
Please note here if the review is uploaded as an attachment.

Reviewer #1

The authors have collected a dataset quantifying the length of fibres and the number of varicosities per unit volume for cholinergic, catecholaminergic and serotonergic neuromodulatory systems in cortex. They have also done an extensive literature search to gather data on the neuromodulatory effects on cortical neurons. Armed with this knowledge they build a detailed computational model to estimate the connectivity, and investigate the effect of synaptic and volumetric transmission on the sleep-state activity of the system.

They estimate the projections from the different fibres onto the neurons, calculating the number of fibres that connect to each neuron, and how many varicosities are formed between the fibre and neuron.

The authors do not directly model the volume transmission, instead choosing to use a phenomenological model where dendrites within a radius r_max are activated. The diffusion process is not modelled, instead the receptors are activated instantly, but the channels have a slower time course, which presumably could account for any transmission delays.

Finally their simulations confirm that ACh desynchronises the sensory cortex, and show that DA 5-HT also desynchronises. In addition 5-HT induces faster oscillatory activity.

Q: On page 6 the Projectionizer tool samples all morphological segments to place synaptic release sites. Do you have fully reconstructed fibres, or is this the dendritic segments of the neurons in all the different layers that are sampled? Based on the rest of the algorithm I assume it is the dendritic segments, but it is not completely clear from the text.

Thanks for the comment. Yes, the Projectionizer tool samples dendritic segments from neurons across all cortical layers, not fully reconstructed axonal fibers. Specifically, all morphological dendritic segments contained in each layer are pooled, and random segments are repeatedly sampled (with replacement) to place new synaptic release sites (sRS) at their centers. The probability of sampling a given segment is proportional to its length, meaning longer dendritic segments are drawn more frequently.

The algorithm does create straight virtual ascending fibers (representing neuromodulatory projections) that begin in layer 6 and extend upward through all cortical layers, but these are the *presynaptic* source fibers, not the targets. The synaptic release sites are then assigned to these fibers based on Gaussian distance distributions. The targets of neuromodulation where the new conductances and synaptic connections are actually placed) are the dendritic segments of the postsynaptic neurons already present in the reconstructed microcircuit.

We have revised the description of the Projectionizer workflow in the Methods section (Pages 10-11 in the Track Changes version).

(Q): From my understanding you only have density estimates, not the full fibres. If you do have the full fibres, why not use a touch detection algorithm to place the synapses?

We thank the reviewer for raising this aspect. To clarify, we only have density estimates from stereological measurements, not fully reconstructed neuromodulatory fiber morphologies. The Projectionizer creates simplified virtual fibers (straight vectors extending through cortical layers) rather than anatomically detailed arbors. Given these geometric simplifications, a touch detection algorithm would require realistic branching morphologies that we do not possess. Instead, our approach samples postsynaptic dendritic segments to match experimentally measured varicosity densities and assigns release sites to fibers based on spatial proximity using Gaussian distributions. This method allows us to satisfy anatomical constraints (layer-wise varicosity densities) while acknowledging the lack of detailed presynaptic fiber reconstructions. We have included this information in the revised text. (Lines 362-363 in the Track Changes version)

Q (page 7): Does it matter that the fibres modelled are straight lines, and not bending and turning? I assume that because of the way you assign synapses to fibres moderate jittering of the fibres might not matter?

Modeling fibers as straight vertical trajectories is a simplification. However, introducing realistic bending and turning would not substantially alter the results due to our assignment algorithm. Synapses are assigned to fibers based on proximity using a Gaussian distance distribution, meaning that release sites within a defined sampling radius have similar probabilities of being assigned to nearby fibers regardless of minor geometric variations.

The critical parameter is this effective sampling radius, which determines the spatial scale over which fiber geometry matters. This radius is biologically constrained by the sphere of influence (Rmax) used in our volume transmission model: 5 μm for acetylcholine, 2 μm for dopamine, and 3 μm for serotonin. These values are derived from experimental measurements of neuromodulator spatial spread in cortical tissue by Borden et al. (2020), Beyene et al. (2019), and Bunin & Wightman (1998).

Since typical fiber curvature over these short spatial scales (2-5 μm) is minimal compared to the sampling radius, moderate jittering or realistic meandering of fiber trajectories would primarily redistribute synapses among nearby fibers without significantly changing the aggregate connectivity statistics, such as the number of neurons contacted per fiber or the layer-wise distribution of innervation. The straight-fiber assumption therefore represents a computationally efficient simplification that preserves the biologically relevant spatial statistics of neuromodulatory innervation constrained by our experimental density measurements. We have included this information in the revised text (Lines 360-374 in the Track Changes version)

Q (page 7): The synaptic release sites are assigned to the different fibres. Does the number of fibres matter for the desynchronisation of the sensory cortex? (In the extreme case with just one fibre there should be a difference since all releases would then be synchronised, but within the range you considered does it differ based on the number of fibres?)

The number of fibers matters for network desynchronization. We systematically tested this by varying the proportion of recruited afferent fibers from 10% to 100% during stimulation. Our simulations show that increasing the percentage of activated fibers enhances the desynchronization effect, as more fibers distribute neuromodulatory release more broadly across the network.

As you correctly note, in the extreme case with a single fiber, all release sites assigned to that fiber would be activated synchronously, creating highly correlated modulatory input that would fail to produce the spatially distributed, asynchronous modulation characteristic of physiological neuromodulatory systems. At the other extreme with all fibers active, release events are distributed across many independent spike trains (approximately 20 Hz stimulation per fiber), creating spatially and temporally dispersed modulation throughout the circuit.

Within the biologically plausible range we modeled (283 cholinergic fibers, 102 dopaminergic fibers, and 53 serotonergic fibers) the number of fibers determines the spatial granularity and temporal independence of neuromodulatory release. A higher fiber count produces finer-grained spatial coverage and more temporally decorrelated release events across the network, which more effectively disrupts synchronized oscillatory activity. Our results demonstrate that recruiting a larger proportion of the available fibers progressively strengthens desynchronization, confirming that fiber number is a critical parameter governing the circuit-level effects of neuromodulation.

This dependency on fiber recruitment also has physiological relevance, as it suggests that graded activation of subcortical neuromodulatory nuclei (e.g., nucleus basalis for acetylcholine, VTA for dopamine, dorsal raphe for serotonin) can produce dose-dependent control over cortical state transitions.

We tested recruiting an increasingly higher percentage of fibers as can be seen in the figure below, which we have added as a supplementary figure (Supplementary Figure S2). We have included the above discussion in the manuscript text, as well (Lines 706-712 in the Track Changes version).

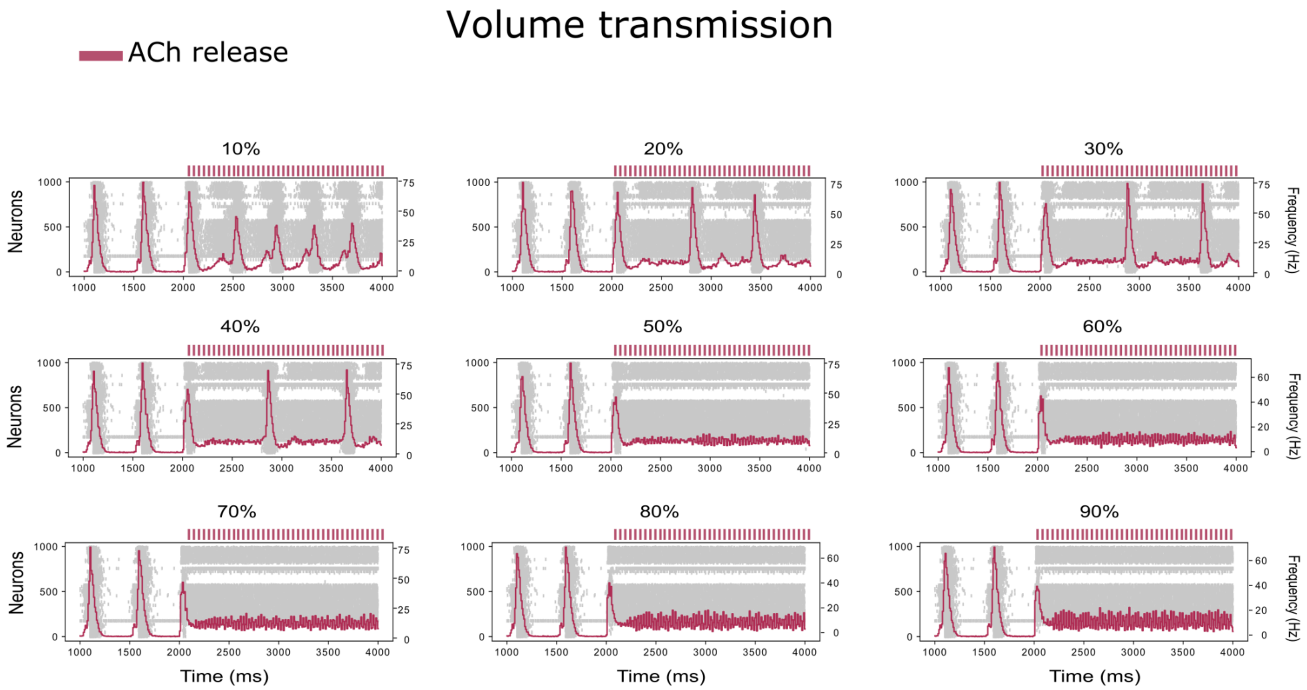

Supplementary Figure S2 - Effects of ACh volumetric transmission
Simulated network effects during the progressive activation of the VT cholinergic projection system. Timing of simulated optogenetic ACh release is shown as colored vertical bars on top of the raster plots. Simulation time is 4000 ms, and projection activation occurs at t = 2000 ms and stops at t = 4000 ms.

Q (page 7 and 8): Given that you simulate the network and study the synchronisation it would be instructive to see the time course of the different synapse types plotted. Right now the parameters are listed in table 2, but the effect of facilitation and depression and the decay time constants might not be immediately obvious.

We agree that the temporal effects of short-term facilitation, depression, and decay kinetics are not immediately obvious from parameter tables alone. In our model, however, neuromodulatory synapses do not introduce novel synaptic dynamics but instead reuse the same well-characterized synaptic types (s-types: E1, E2, I2, I3) defined in the Neocortical Microcircuit (NMC) model (Markram et al., 2015). The corresponding postsynaptic potential (PSP) time courses, including the effects of facilitation, depression, and decay time constants, are therefore identical to those already documented and validated in the NMC framework. We have explicitly added in Table 2 legend direct links to representative PSP examples for each s-type (E1, E2, I2, I3) hosted on the NMC portal. This allows interested readers to inspect the full time course of these synaptic dynamics without duplicating material already described.

Q (page 8): Why did you not include different delays dependent on the distance to the release site? (Delay difference would be small?) Do you allow two release sites to affect a target site? (Ie, how close is your phenomenological model to the real volume transmission case).

We did not include distance-dependent delays in our volume transmission model. This is because at the spatial scales relevant to neuromodulatory diffusion (2-5 μm), temporal delays are negligible compared to the timescales of neuromodulatory action.

To quantify this, assuming a diffusion coefficient for acetylcholine in brain tissue of approximately 3.3 × 10^{-6} cm^2/s (Dani & Bertrand, 2007; Sarter et al., 2009), the time for ACh to diffuse 5 μm is on the order of ~10 milliseconds (using $t \approx r^2/6D$). For dopamine and serotonin with similar diffusion coefficients over their respective Rmax values (2-3 μm), delays would be even shorter. These delays are orders of magnitude smaller than the decay time constants we use for volume transmission (hundreds of milliseconds, Table 2) and the timescale of neuromodulatory effects on network dynamics. Moreover, experimental data characterizing distance-dependent propagation delays for neuromodulatory signals at micrometer resolution in cortical tissue are not available in the

literature. The studies we used to constrain Rmax (Borden et al., 2020; Beyene et al., 2019; Bunin & Wightman, 1998) measured spatial extent of transients but not fine-grained temporal propagation dynamics. In the absence of such data, we assumed simultaneous activation of all targets within the sphere of influence, which is a reasonable approximation given the negligible delays relative to the biological timescales being modeled.

Our model allows multiple volume release sites (vRS) to affect the same target. When a dendritic segment falls within the spheres of influence of multiple vRS, it receives conductances from each, with amplitudes scaled by distance from each respective vRS. However, the likelihood of significant overlap is relatively low given the sparse density of neuromodulatory varicosities compared to intracortical glutamatergic synapses. For context, our experimentally measured varicosity densities are on the order of 10^5-10^7 per mm^3 (Table 3), whereas glutamatergic synapse density in rodent cortex is approximately 6-8 × 10^8 per mm^3. Combined with the small Rmax values, most dendritic segments receive input from zero to a few vRS. Where overlap does occur, the conductances summate linearly, providing a phenomenological approximation of additive neuromodulatory signals converging on the same postsynaptic site, consistent with the biology of extrasynaptic receptor activation.

Our volume transmission model makes several simplifying assumptions: (1) isotropic diffusion (spherical spread), (2) linear decay of peak concentration with distance, (3) neglect of temporal wave propagation, and (4) no explicit modeling of uptake/degradation spatial heterogeneity. These are phenomenological approximations necessitated by limited experimental constraints. The model captures the key distinction from synaptic transmission (one-source-to-many-targets spatial divergence and the slower kinetics of extrasynaptic signaling) while remaining tractable and constrained by available data. We specify the absence of delays at Lines 464-466 in the Track Changes version)

Q (page 15): From what I understand the number of synapses a neuron receives will be directly proportional to the total dendritic length, and for volume transmission it depends on dendritic length and layer varicosity density? You mention the two most contacted neuron types, but do not explain why they are most connected.

For synaptic transmission (ST), the number of synapses a neuron receives is proportional to its total dendritic length weighted by the layer-wise varicosity density at each depth. Specifically, dendritic segments are sampled with probability proportional to their length, and synaptic release sites (sRS) are placed to match the experimentally measured varicosity density in each layer. Therefore, neurons with more extensive dendritic arbors spanning layers with higher varicosity density receive proportionally more synaptic contacts.

For volume transmission (VT), the placement of release sites follows the same initial procedure as ST (proportional to dendritic length and layer-wise varicosity density). However, each release site becomes a volume release site (vRS) that acts as the center of a sphere of influence with radius Rmax (5 μm for ACh, 2 μm for DA, 3 μm for 5-HT). We then resample all dendritic segments within this sphere and place conductances randomly along each segment to model extrasynaptic receptor activation. This creates a one-source-to-many-targets connectivity pattern where a single vRS affects multiple nearby neurons. Consequently, VT innervation depends on both the neuron's dendritic length and the density of volume release sites in the layers it occupies.

Why TPC and LBC are most contacted: Thick-tufted pyramidal cells (TPC) among excitatory morphological types and large basket cells (LBC) among inhibitory morphological types exhibit the greatest total dendritic lengths in our reconstructed microcircuit. Because synaptic site placement is weighted by dendritic length, and volume transmission samples all segments within spheres of influence, these morphological types with the most extensive dendritic arbors are necessarily the most heavily contacted by all three neuromodulatory systems. This is a direct geometric consequence of our sampling algorithm combined with the morphological diversity present in the circuit: cells with larger dendritic fields simply present more surface area for potential neuromodulatory contact, both synaptically and via volume transmission.

We have added this comment in the Results section (Lines 614-623 in the Track Changes version)

Q (Figure 4): Is it correct that there are so few target neurons in layer 1, the varicosity density is highest there? (Figure 4B)

Yes, this is correct. Layer 1 contains very few neuronal somata as it is the least populated layer and consists exclusively of inhibitory interneurons. Therefore, the counts of neurons with somata in layer 1 shown in Figure 4B are necessarily low.

However, this does not reflect the full picture of neuromodulatory innervation in layer 1. The high varicosity densities observed in layer 1 (Figure 3D-F) result in a considerable number of synaptic contacts on dendritic processes from neurons whose somata are located in deeper layers. Specifically, the apical tuft dendrites of most pyramidal cell types (particularly TTPC in layers 2/3 and 5) terminate and arborize extensively in layer 1. This creates a high density of dendritic membrane surface area available for neuromodulatory contact despite the paucity of local cell bodies.

The combination of dense dendritic terminations in layer 1 and high neuromodulatory varicosity density in that layer means that layer 1 is a critical site for neuromodulatory influence on cortical circuits, primarily through modulation of the distal apical dendrites of pyramidal neurons. This spatial arrangement allows neuromodulators to preferentially affect the apical integration compartments of pyramidal cells, which play important roles in top-down processing, attentional modulation, and dendritic computations. Our sampling algorithm correctly captures this: while few neuronal somata reside in layer 1, the dendritic segments pooled from that layer include extensive apical arbors from deeper-layer pyramidal cells, resulting in substantial neuromodulatory connectivity despite low somatic density.

Q: **(Figure 5E)**: The second and third plot show experimental data that justify your choice of r_max. Please change the wording, this is not direct validation. (Or is there a model data line missing in the plot?)

We thank the reviewer for highlighting this. Indeed, this is not a direct validation, and we have revised the wording accordingly from "partial validation" to "supporting evidence."

To clarify: We did not use the experimental data shown in Figure 5E to choose Rmax. The Rmax values were constrained by independent studies that directly measured the spatial extent of neuromodulatory transients in brain tissue:

ACh: Rmax = 5 μm from Borden et al. (2020)
DA: Rmax = 2 μm from Beyene et al. (2019)
5-HT: Rmax = 3 μm from Bunin & Wightman (1998)

These studies used genetically encoded fluorescent sensors or electrochemical detection to measure how far neuromodulatory signals propagate from release sites.

Figure 5E presents different experimental evidence that was not used during model construction but provides supporting evidence for our implementation choice. Specifically, Figure 5E shows experimental data demonstrating that synaptic conductances or neuromodulatory effects decrease as a function of distance from the release site. This distance-dependent attenuation is consistent with our modeling approach of scaling conductance values inversely with distance (ranging from 1.0 at the release site to 0.1 at Rmax).

While Figure 5E does not directly validate our specific Rmax values or scaling function, it supports the general principle that neuromodulatory influence diminishes with distance, which is a key assumption underlying our volume transmission model. This independent experimental evidence for distance-dependent modulation strengthens confidence in our phenomenological approach.

(Page 18): Your simulations nicely show desynchronisation, but there is no discussion about why there is a difference between the synaptic and volumetric transmissions. What are the mechanisms? Do the two preferentially target slightly different neurons, or is it the temporal pattern of innervation, or the time course of the synapses, that matters for the strength of the desynchronisation? It would be great to understand what it is that makes them different (given your assumptions in the model).

Thank you for this insightful comment. We have added a more thorough discussion about the mechanisms underlying the differential desynchronization power of synaptic transmission (ST) versus volume transmission (VT).

Key mechanistic differences:

1. Spatial divergence and total conductance: As illustrated in Figure 5A, VT contacts substantially more target neurons than ST due to the sphere-of-influence architecture. Each volume release site (vRS) affects all dendritic segments within its Rmax radius (2-5 μm depending on neuromodulator), whereas ST creates one-to-one connections between release sites and specific dendritic segments. This results in a much larger overall conductance being delivered to the network under VT conditions.

Figure 6B demonstrates the functional consequence: recruiting only 10% of VT fibers produces a network effect comparable to recruiting 100% of ST fibers, due to the sheer number of dendritic segments being simultaneously targeted. However, this same figure reveals that increasing the fraction of recruited VT fibers beyond ~30-40% induces desynchronization so strong that it cannot be considered physiologically realistic as the network activity becomes excessively suppressed. This suggests that pure volume transmission, if operating at full capacity across all neuromodulatory fibers, would produce implausibly strong effects.

2. Temporal dynamics: VT employs slower decay kinetics (greater tau decay, on the order of hundreds of milliseconds, Table 2) compared to ST (millisecond-range kinetics). This prolonged time course allows for temporal summation of neuromodulatory responses across multiple release events. The sustained conductance changes under VT create longer-lasting shifts in neuronal excitability and synaptic efficacy, which more effectively disrupts the recurrent excitatory-inhibitory dynamics that sustain synchronized oscillations. In contrast, ST's faster kinetics produce more transient, precisely-timed perturbations that require higher fiber recruitment to achieve comparable desynchronization.

3. Comparison with experimental data: Critically, Figure 6D shows that the mean reduction in delta power observed in optogenetic experiments more closely matches our ST condition than VT, particularly at physiological recruitment levels (50-100% of fibers). This suggests that synaptic transmission is the dominant mode for neuromodulatory control of cortical synchronization under naturalistic conditions, while volume transmission may serve as a supplementary mechanism during intense or sustained neuromodulatory activation.

4. Target selectivity: While both ST and VT sample dendritic segments proportionally to their length and layer-wise varicosity density, VT's spatial divergence means it affects neurons more uniformly across cell types within its spatial reach, whereas ST's one-to-one connectivity introduces more specificity (though still probabilistic in our model). This difference in target selectivity also contributes to the distinct network effects.

The stronger desynchronization under VT results from the combination of greater spatial divergence (more targets per fiber), slower temporal kinetics (enabling summation), and larger total conductance—but our comparison with experimental data suggests that this regime operates below its theoretical maximum capacity under physiological conditions, with ST playing the dominant role in cortical state control.

These mechanisms were already implicit in the Discussion; in the revised manuscript we have now made them explicit by synthesizing spatial divergence, temporal kinetics, and target selectivity into a single mechanistic explanation (Lines 977-993 in the Track Changes version).

(Page 26): Would it be possible to find out why 5-HT promotes oscillation within a specific frequency range? Is there a subpopulation of neurons that are more active? Would ablating connection to a specific population of neurons remove this oscillation?

We thank the reviewer for this insightful question about the mechanistic basis of 5-HT-induced theta oscillations.

The increase in oscillatory activity within the theta frequency range following 5-HT application can be attributed to a perturbation of the network's excitation–inhibition (E-I) balance in favor of inhibition. Specifically, based on the receptor distribution and physiological effects we implemented from literature (Table 7), 5-HT primarily hyperpolarizes pyramidal neurons in layers 5 and 6 while depolarizing interneurons in layers 1 and 2/3. This differential modulation creates conditions favorable for rhythmic inhibitory-paced activity: enhanced inhibition suppresses slower delta oscillations (as shown in our results), while the increased interneuron excitability in superficial layers may entrain network activity into faster theta-range frequencies through paced inhibitory volleys.

Your suggestion to test this hypothesis through selective ablation of connections to specific neuronal populations (e.g., removing 5-HT inputs to superficial interneurons or deep pyramidal cells) would indeed be highly informative and could definitively identify which cell types are necessary and sufficient for theta generation. However, since the

Blue Brain Project ended in December 2024, the simulation framework and computational infrastructure used for this study are neither available nor accessible, making such additional *in silico* experiments currently infeasible.

More fundamentally, our findings regarding the effects of 5-HT (and DA) on network oscillations in the sensory cortex should be regarded as predictions emerging from the integration of available anatomical and physiological data within our modeling framework. Unlike our ACh results, which we could validate against extensive existing literature on cholinergic desynchronization, the oscillatory effects of 5-HT and DA in somatosensory cortex represent novel predictions. These predictions cannot currently be validated experimentally because appropriate data—particularly layer- and cell-type-specific recordings during 5-HT/DA manipulation in the rodent sensory cortex—are lacking in the literature. Therefore, while our model generates testable hypotheses about the circuit mechanisms underlying 5-HT-induced theta oscillations, experimental validation and further mechanistic dissection (including ablation studies) are beyond the scope of this computational study and represent important directions for future experimental work.

We have clarified this distinction in the discussion to emphasize that the 5-HT and DA findings are predictions intended to guide future experiments rather than validated mechanisms (Lines 965-968 in the Track Changes version).

Minor:

Q (page 14): Would the data be more easily digested in a table? (Number of neuron targets per fibre, etc)

Thanks for the suggestion, to facilitate comparison across neuromodulatory systems and transmission modes, key *in silico* predictions of fiber targeting and connectivity are now summarized in Table 4.

(Figure 5B) : Tiny figure, could be made a bit larger so it is easier to see.

Good point, we have revised Figure 5B to improve the readability of the labels.

(Figure 5C): Here you could include some example synaptic and volumetric transmission time courses from Table 2.

We have revised Figure 5C to make the example current traces more quantitative by adding scale bars to indicate the relevant timescales. Because Figure 5C is intended to illustrate general model behavior rather than specific synaptic instances, we did not include additional example traces. Instead, we now provide direct links in the legend of Table 2 to representative postsynaptic potential time courses for each synaptic type (E1, E2, I2, I3) hosted on the NMC portal, allowing interested readers to inspect detailed synaptic dynamics without duplicating previously published material.

Reviewer #2

This interesting paper shows that in a computational model of rat neocortex, cholinergic, domaminergic, and serotonergic activation disrupt oscillatory dynamics, and that volume transmission has a bigger impact than synaptic transmission. Immunostaining has been used to estimate the distribution of some of the synapses modeled, with most of the model parameters coming from existing literature.
The paper is generally well written and presented.
The model is large, with many parameters, although modeling appears robust and assumptions well justified.
Some potential improvements:

Many thanks for your positive evaluation of our work.

Q. Most parameters are specified as estimates within a range. Although it may not be feasible to perform a full sensitivity analysis of all parameters, it would be feasible to do so for some of the key assumptions e.g. number of serotonergic/dopaminergic fibers.

We completely agree that sensitivity analyses can be valuable for assessing the robustness of model predictions. However, given the scale and complexity of the model, even a limited parameter scan (e.g., varying the number of serotonergic or dopaminergic fibers) would substantially expand the scope of this study. In addition, the large-scale simulation framework and computational infrastructure used for this work were developed within the Blue Brain Project, which concluded in December 2024 and is no longer accessible for additional simulations. The primary aim of this paper is therefore to present a coherent methodological framework that integrates available (and sometimes conflicting) data and reproduces key network-level behaviors. The model is built on explicitly stated assumptions, which we view as testable hypotheses that can be explored in future work building on the framework and tools presented here.

We have clarified these aspects in the Discussion (Lines 965-968 and 1061-1071 in the Track Changes version).

Q. Figures 6,7,8 panel D is not sufficiently well explained, text could also be bigger in these panels.

Thanks for the suggestion. We have revised and increased the font size in Figures 6, 7, and 8. For each panel D, we have improved the legend to be more informative.

Reviewer #3:

This paper presents an interesting and useful combination of anatomical data and computational simulations of network dynamics. The paper presents the anatomical quantification of neuromodulatory fibers in the somatosensory cortex arising from cholinergic, dopaminergic and serotonergic neurons. The paper also presents computational simulations of the influence of these modulators on the oscillatory network dynamics of this cortical circuit. This type of research is valuable overall for linking detailed anatomical and cellular level data to network level dynamics. The most important long-term benefit is to link the molecular details of the expression of specific proteins mediating membrane conductances to complex network dynamics. Given the importance of this linkage, the paper needs to be revised to make that linkage to membrane conductances explicit. This means modifying tables that describe the effects of neuromodulators that are summarized in an oversimplified manner in tables 5-7 without any mention of the modulation of the membrane conductances that underlie these effects. Currently, the tables have an overly simplistic summary of ACh effects as EPSCs and IPSCs that does not accurately reflect the Hodgkin-Huxley equations used to simulate membrane conductances in the basic model they are using from the earlier paper (Markram et al., 2015). This important revision is further itemized in the major comments below along with some additional important comments in the major and specific comments.

Thank you for this important comment, which allows us to clarify a central aspect of our modeling approach. We fully agree that linking neuromodulatory effects to membrane conductances is essential for understanding network dynamics. However, we wish to emphasize a crucial distinction between two fundamentally different modeling strategies for implementing neuromodulatory effects:

Approach 1: Direct modulation of intrinsic ionic channels
This approach involves modifying the intrinsic Hodgkin-Huxley conductances (g_Na, g_K, g_Ca, g_leak) that govern baseline neuronal excitability. Examples include muscarinic suppression of the M-current, modulation of voltage-gated calcium channels, or alterations in leak conductances. This requires detailed, receptor subtype-specific knowledge of intracellular signaling cascades (e.g., G-protein coupled receptor pathways, second messenger systems, phosphorylation states).

Approach 2: Addition of neuromodulator-gated conductances (our approach)
Our model implements neuromodulatory effects by adding *new* neuromodulator-sensitive conductances to postsynaptic neurons. These added conductances are parameterized to reproduce experimentally observed net depolarizing or hyperpolarizing effects on membrane potential and firing probability. Critically, these are distinct from and supplementary to the intrinsic Hodgkin-Huxley ionic channels of the underlying cortical neuron models.

We adopted Approach 2 for several compelling reasons:

1. Insufficient quantitative constraints: Cell-type- and layer-specific quantitative data on receptor subtype expression (nicotinic vs. muscarinic; D1 vs. D2; 5-HT1A vs. 5-HT2A), their effects on specific ionic channels, and the associated intracellular signaling dynamics remain insufficiently constrained, particularly in developing somatosensory cortex at P14.
2. Complexity of molecular mechanisms: Neuromodulators act through complex, incompletely understood G-protein coupled receptor cascades involving second messengers, protein kinase activation, and downstream modulation of multiple ionic conductances. These mechanisms vary substantially across cell types and developmental stages.
3. Compatibility with existing models: Our approach captures the net functional impact on excitability while remaining fully compatible with the biophysically detailed Hodgkin-Huxley neuron models from the underlying Markram et al., 2015 microcircuit.
4. Focus on network-level dynamics: As stated in our manuscript (page 3): *"This phenomenological approach captures the net functional impact on membrane potential and firing probability, while remaining fully compatible with the biophysically detailed neuron models of the underlying neocortical microcircuit. Importantly, this framework does not attempt to explicitly model receptor subtype-specific intracellular signaling cascades, which remain insufficiently constrained across cell types and developmental stages".*

Our model captures:

- Net depolarizing/hyperpolarizing effects on membrane potential
- Changes in firing probability and excitability
- Temporal dynamics of neuromodulatory effects (via decay time constants)

- Spatial organization of neuromodulatory inputs (synaptic vs. volume transmission)

Our model does not explicitly capture:

- Receptor subtype-specific effects (e.g., nicotinic α7 vs. α4β2; muscarinic M1 vs. M2)
- Direct modulation of intrinsic voltage-gated ion channels
- Presynaptic modulation of glutamate or GABA release
- Intracellular signaling cascades and second messenger dynamics

We revised the Introduction (Lines 207-219 in the Track Changes version) to clarify the scope of the model from the beginning.

We fully acknowledge that incorporating receptor-specific intracellular mechanisms and direct modulation of intrinsic membrane channels represents an important direction for future work as more quantitative, cell-type-specific data become available. Such advances would enable a transition from phenomenological to mechanistic modeling, potentially revealing how molecular-level changes in specific ionic conductances give rise to the network-level dynamics we observe. We have added this perspective in the Discussion section (Page 40 in the Track Changes version).

Major comments:

1. Table 5 – The tables and text need to be revised to remove the oversimplification that summarizes the effects of ACh as "EPSC" or "IPSC." The use of the terms EPSC might be appropriate for rapid effects of nicotinic receptor activation, but it is not appropriate for the majority of cholinergic effects given that most intracellular recording studies in slices have shown very slow depolarization of neurons due to blockade of the leak K+ conductance, which would not qualify as an "EPSC".

Note: for the rest of the review, to avoid confusion, we keep the numbering of Tables before the revision (i.e., relevant Tables here are 5, 6, and 7). In the revised paper, old Table 5 is now Table 6, old Table 6 is now Table 7, etc.

We thank the reviewer for this important clarification, which highlights a critical distinction in neuromodulatory mechanisms. We fully acknowledge that our use of "EPSC" and "IPSC" terminology in Table 5 (and Tables 6-7) may be misleading and requires substantial revision.

The reviewer correctly points out that many cholinergic effects, particularly those mediated by muscarinic receptors, involve slow depolarization through blockade of leak K^+ conductances rather than rapid synaptic-like currents. We agree that the term "EPSC" (excitatory postsynaptic current) inappropriately implies fast, ligand-gated receptor-mediated currents typical of classical synaptic transmission, whereas most neuromodulatory effects operate through G-protein coupled receptor (GPCR) cascades that modulate intrinsic membrane conductances over slower timescales.

To address this concern, we have revised Table 5 and associated text and replaced "EPSC/IPSC" terminology with more accurate descriptors such as "Net depolarizing current" or "Net hyperpolarizing current" and "depolarization/hyperpolarization" instead of EPSP/IPSP.

We have revised the Discussion as well (Lines 878-893 in the Track Changes version), to highlight that our phenomenological conductance-based approach captures the net functional effects of neuromodulation on cellular excitability, but does not explicitly model the underlying molecular mechanisms. Future iterations of this model could incorporate these receptor subtype-specific mechanisms as more quantitative, cell-type-resolved data become available from the developing somatosensory cortex.

In addition, the terms EPSC and IPSC completely ignores the important effect of muscarinic receptors on other membrane conductances such as blocking the K(AHP) channel or the M-current, both effects which cannot be described as an EPSC or an IPSC.

We thank the reviewer for emphasizing this critical point. We completely agree that muscarinic receptor-mediated effects, including suppression of the afterhyperpolarization K^+ current (K_AHP), M-current (K_M), and leak K^+ conductances, involve modulation of intrinsic membrane conductances that are fundamentally distinct from classical fast synaptic currents. The use of "EPSC" and "IPSC" terminology was indeed inappropriate and we have amended the manuscript accordingly.

The reviewer correctly identifies that a mechanistically accurate model would directly modify specific Hodgkin-Huxley conductances (g_K(AHP), g_K(M), g_leak, etc.) in the underlying cortical neuron models. However, we adopted a phenomenological approach for the following reasons:

1. Insufficient cell-type-specific quantitative data. To directly model muscarinic suppression of K(AHP) or M-current, we would need quantitative data for each of the 55+ cortical cell types (m-types) in our circuit regarding:

- Baseline expression levels of specific K^+ channel subtypes (Kv7/KCNQ for M-current, SK/BK channels for K_AHP)
- Muscarinic receptor subtype expression (M1-M5) and their coupling to specific intracellular signaling pathways
- The magnitude of conductance suppression as a function of receptor activation across different neuromodulator concentrations

Such comprehensive, cell-type-resolved datasets do not currently exist for the developing somatosensory cortex. We attempted to re-optimize the intrinsic parameters of our neuron models based on experimental data examining carbachol effects on different cell types, but this effort was unsuccessful due to insufficient data; specifically, the absence of concentration-response curves across multiple cell types that would allow us to constrain the dose-dependent modulation of specific conductances.

2. Complexity of muscarinic signaling cascades. As noted by the reviewer, muscarinic receptor activation involves complex G-protein coupled receptor signaling cascades that modulate multiple ionic conductances simultaneously through distinct intracellular pathways:

M1/M3/M5 receptors couple to Gq proteins → phospholipase C activation → PIP2 depletion → M-current suppression

M2/M4 receptors couple to Gi/o proteins → adenylyl cyclase inhibition → reduced cAMP → effects on multiple downstream targets

Additional modulatory effects on voltage-gated Ca^{2+} channels, hyperpolarization-activated currents (I_h), and presynaptic transmitter release

The net cellular effect depends on the complement of receptor subtypes expressed, the basal activity of intracellular signaling pathways, and the phosphorylation state of ion channels—all of which vary across cell types and are incompletely characterized.

Given these constraints, we model neuromodulatory effects by adding receptor-mediated conductances that reproduce the experimentally observed net changes in membrane potential, excitability, and firing patterns. This approach:

- Captures the functional outcome (e.g., depolarization, increased input resistance, enhanced firing) without requiring detailed knowledge of which specific ionic conductances are modulated
- Remains compatible with the biophysically detailed Hodgkin-Huxley neuron models from Markram et al., 2015
- Allows for a systematic exploration of how anatomically grounded neuromodulatory inputs shape network dynamics

For example, muscarinic suppression of M-current produces depolarization and increased excitability. Our model achieves the same functional effect through an added excitatory conductance with appropriate reversal potential and kinetics, constrained by experimental recordings of membrane potential changes and firing rate modulation.

Finally, this summary also completely ignores the large literature showing muscarinic presynaptic inhibition of glutamatergic and GABAergic synaptic transmission.

We thank the reviewer for raising this important point. We are indeed aware of the extensive literature documenting muscarinic presynaptic inhibition of both glutamatergic and GABAergic synaptic transmission, as well as other complex phenomena such as co-release of neuromodulators with classical neurotransmitters. These mechanisms represent crucial aspects of neuromodulatory function that can significantly influence network dynamics.

We made a deliberate decision to exclude presynaptic modulation from the current implementation for the following reasons:

1. Models are inherently scoped representations of biological systems, they cannot capture every known biological mechanism simultaneously without becoming intractable. Our study focuses specifically on postsynaptic neuromodulatory effects on cellular excitability as mediated through receptor activation on target neurons. This scope was chosen because:

- We have anatomical data constraining the spatial distribution of neuromodulatory varicosities across cortical layers
- We have physiological data (primarily from optogenetic studies) characterizing postsynaptic responses to neuromodulator release
- The existing Markram et al., 2015 circuit model provides detailed baseline synaptic connectivity and dynamics that can be modulated

2. To implement presynaptic modulation reliably, we would require cell-type-specific, synapse-type-specific quantitative data on the magnitude of presynaptic inhibition for each of the hundreds of connection types (m-type to m-type pairs) in the circuit, the spatial organization of presynaptic muscarinic, nicotinic, dopaminergic, and serotonergic receptors on axon terminals of different neuron types, the kinetics of presynaptic modulation (onset, duration, recovery), and potential synapse-specific heterogeneity in presynaptic receptor expression

Such comprehensive datasets do not currently exist. Without specific quantitative data, incorporating presynaptic effects would require numerous unconstrained assumptions that could introduce systematic errors into our network simulations.

3. Presynaptic neuromodulation involves multiple mechanisms (e.g., direct modulation of voltage-gated Ca^{2+} channels at the active zone, modulation of vesicle priming and release probability, etc.). Each mechanism may operate on different timescales and exhibit different degrees of synapse specificity. Implementing these effects accurately would require extensive parameterization that is not currently feasible given available data.

4. Our current framework already incorporates substantial complexity:

- Three distinct neuromodulatory projection systems (ACh, DA, 5-HT and synaptic vs. volume transmission)
- 55+ cortical cell types with distinct anatomical and physiological properties
- Anatomically constrained spatial distribution of neuromodulatory inputs across layers
- Distance-dependent scaling of volume transmission effects

Adding presynaptic modulation across hundreds of synapse types would substantially increase model complexity and the number of unconstrained parameters, potentially compromising interpretability and making it difficult to isolate the specific mechanisms driving observed network dynamics.

We fully acknowledge that presynaptic modulation represents a significant gap in the current model. We added this discussion in the Limitations (Lines 1061-1071 in the Track Changes version).

All of these factors should be revised to make a clearer link between the network simulations, the experimental in vivo physiology data and the means by which the computational model implements cholinergic modulation based on an extensive literature of intracellular recording of membrane conductances as well as molecular work on the modulation of membrane conductances by cholinergic receptors.

We appreciate the reviewer's emphasis on establishing clear connections between our computational implementation, the extensive intracellular recording literature on membrane conductance modulation, and in vivo physiological data. We comprehensively revised the manuscript to strengthen these linkages. In the Introduction (Lines 215-219 in the Track Changes version) and in the Discussion sections (Lines 895-907 in the Track Changes version) we have highlighted how our phenomenological modeling approach does not explicitly simulate the known molecular mechanisms and how, instead, we capture their net functional outcome.

2. Table 6 – The descriptions in table 6 are slightly more sophisticated in terms of referring “increase excitability” which could correspond to an effect on a membrane conductance such as those modeled by Durtstewitz and Seamans, but the presentation needs to be revised to describe how their compartmental biophysical simulation simulates these effects in the Hodgkin Huxley framework which is the basis for modeling done using Neuron and presented in the earlier paper by Markram et al., 2015. I find it surprising that there currently is no explicit mention of these conductances that are so essential to this type of modeling, so the paper needs to be revised to make this

connection explicit. They can reference the earlier paper, but need to summarize the overall effects in terms of membrane conductances.

We thank the reviewer for this valuable feedback and for highlighting the important work by Durstewitz and Seamans on dopaminergic modulation of prefrontal cortex neurons. The reviewer correctly notes that the terminology "increase excitability" in Table 6 could correspond to effects on specific membrane conductances as modeled by Durstewitz and Seamans. However, there is a critical distinction between their approach and ours:

Durstewitz, Seamans and Sejnowski (Durstewitz et al., 2000; Durstewitz & Seamans, 2002) approach:

- Directly modulated intrinsic Hodgkin-Huxley ionic conductances in their compartmental neuron models
- Explicitly simulated D1 receptor effects: reduction of Na^+ conductance (g_Na), enhancement of persistent Na^+ current, modulation of voltage-gated K^+ conductances
- Explicitly simulated D2 receptor effects: enhancement of K^+ conductances, reduction of Ca^{2+} conductances
- Used these receptor-specific conductance modifications to explore computational consequences for prefrontal cortex function

Our approach:

- We do not modify the intrinsic Hodgkin-Huxley ionic conductances (g_Na, g_K, g_Ca, g_leak) of the cortical neurons in the Markram et al., 2015 circuit model
- Instead, we add new dopamine-sensitive conductances that represent activation of dopamine receptor clusters on postsynaptic neurons
- These added conductances are parameterized to reproduce the net functional effects (depolarization, hyperpolarization, changes in excitability) observed experimentally
- This phenomenological approach captures the overall outcome of dopamine receptor activation without explicitly modeling receptor subtype-specific modulation of intrinsic ionic channels

We could not find sufficient data from optogenetic stimulation of dopaminergic fibers projecting specifically to somatosensory cortex. The vast majority of dopamine neuromodulation studies have focused on:

- Prefrontal cortex (PFC), where Durstewitz, Seamans, and others have extensively characterized D1 and D2 receptor effects
- Striatum, where dopaminergic modulation is well-characterized
- Motor cortex in the context of motor learning

Dopaminergic innervation and function in primary somatosensory cortex remain understudied relative to these other regions.

However, our approach remains firmly grounded in the conductance-based HH framework that underlies NEURON simulations and the baseline Markram et al., 2015 model. Specifically:

The Markram et al., 2015 neuron models contain:

- Voltage-gated Na^+ conductances (g_Na) for action potential generation
- Voltage-gated K^+ conductances (g_K) for repolarization
- Voltage-gated Ca^{2+} conductances (g_Ca) in dendrites
- Leak conductances (g_leak)
- Ca^{2+}-activated K^+ conductances in some cell types
- Hyperpolarization-activated conductances (I_h) in some cell types

Our dopamine modulation adds:

- New dopamine-gated conductances (g_DA) placed on dendritic segments
- Each g_DA has a reversal potential (E_rev) that determines whether the conductance is excitatory or inhibitory
- Time-dependent activation and decay kinetics (τ_rise, τ_decay)
- Spatial distribution constrained by anatomical varicosity density data

We added a Discussion subsection comparing our approach for DA modeling to more mechanistic implementations, such as the one proposed by Durstewitz, Seamans and Sejnowski (Lines 1082-1110 in the Track Changes version).

3. **Table 7** – the summary of effects of 5-HT is similarly oversimplified and makes no mention of the underlying membrane conductances. Making the explicit connection to membrane conductances will also allow the descriptions in the tables to be consistent across tables, as I do not understand how they can currently justify describing the effects of these modulators using completely different terminology for each modulator when the effects of the modulators are all mediated through a similar set of membrane conductances (though obviously via different receptors). These differences are due to the difference in effect on a large range of different membrane conductances and this must be acknowledged and explicitly described in the paper.

We appreciate the reviewer's concern regarding the relationship between neuromodulatory effects and underlying membrane conductances. As clarified in our response to previous comments, our modeling approach does not attempt to explicitly represent receptor subtype–specific modulation of individual ionic channels (e.g., M-current, K(AHP), presynaptic mechanisms). Instead, neuromodulatory effects are implemented as added conductances that capture the net, experimentally observed impact of 5-HT on neuronal excitability in a cell-type-specific manner.

Table 7, like Tables 5 and 6, summarizes these experimentally reported functional effects (e.g., depolarization, hyperpolarization, changes in excitability), which are then translated into corresponding conductance-level implementations in the model. The intention is not to imply distinct mechanistic frameworks across neuromodulators, but rather to reflect differences in experimentally observed cellular responses while maintaining a consistent modeling abstraction across ACh, DA, and 5-HT.

To address the reviewer's concern, we have clarified in the manuscript that all neuromodulators are implemented within the same conductance-based framework, and that the tables describe functional constraints used to parameterize these added conductances rather than detailed channel-level mechanisms.

4. To continue on this same point, they need to be clearer about how the electrophysiological properties of the neurons were modeled based on the evidence from intracellular recording of neurons in slices and studies of neuromodulator effects on different membrane conductances. The introduction should note that they draw on previous intracellular recording studies showing that ACh causes nicotinic and muscarinic modulation of membrane conductances (i.e. Schwindt and Crill, Madison and Nicoll) as well as causing presynaptic inhibition of both excitatory and inhibitory synapses (Valentino and Dingledine, 1981; Behrends and ten Bruggencate, 1993; Hasselmo, 1995; Hasselmo and Schnell, 1996). These types of physiological studies were presumably used to guide the simulations of cholinergic modulation, but they do not seem to be mentioned much in the introduction.

We thank the reviewer for this important suggestion. We have revised the Introduction (Lines 195-205 in the Track Changes version) to more explicitly acknowledge the extensive intracellular and synaptic physiology literature underlying cholinergic modulation. In particular, we now cite whole-cell and voltage-clamp studies demonstrating nicotinic depolarization and muscarinic modulation of intrinsic membrane conductances (e.g., Madison & Nicoll, 1984), as well as presynaptic inhibition of excitatory and inhibitory transmission (Valentino & Dingledine, 1981; Behrends & ten Bruggencate, 1993; Hasselmo, 1995; Hasselmo & Schnell, 1994). These studies provide the physiological foundation for our conductance-level implementation of cholinergic modulation. The revised text clarifies that while our model does not explicitly simulate individual receptor subtype cascades, it is grounded in this well-established electrophysiological literature.

5. In addition to the methods and results needing to be revised to make the explicit connection to membrane conductances, the paper was somewhat confusing because there was not a sufficient overview of the methods for using anatomical data to estimate connectivity. In particular, they need to more clearly state if their estimates of connectivity were based purely on density of fibers and location of cell types. For example, on **Page 14** – it is not clear if they were estimating these contact probabilities purely based on density and not based on EM studies of contact probabilities by identified neurons? If you aren't using any prior studies, you should mention that you are doing it based only on densities of fibers and neuron location.

We thank the reviewer for highlighting this point. Neuromodulatory connectivity in our model is inferred solely from experimentally measured varicosity densities and the spatial distribution of postsynaptic dendritic segments in the reconstructed microcircuit. We do not use EM-derived contact probabilities between identified pre- and postsynaptic neuron types, as such data are not currently available for ascending neuromodulatory systems in sensory cortex. Specifically, synaptic release sites are placed to match the measured layer-wise varicosity density, and postsynaptic targets are selected based on dendritic segment length and spatial location. Thus, estimates of

connectivity arise from the combination of (i) experimentally constrained varicosity density and (ii) the geometry and laminar organization of neuronal morphologies in the circuit. No additional assumptions about cell-type-specific contact probabilities were introduced.

We have revised the Methods section to explicitly state this modeling assumption to avoid ambiguity (Lines 374-377 in the Track Changes version).

6. Page 6 – “estimation of fiber length” – The heading and text in the methods section refers to estimation of fiber length (which the name suggests would presumably be a one-dimensional measurement) and never mentions “fiber length density” and yet the Results section focuses on fiber length density (which is presumably the number of fiber segments per unit area). This discrepancy in terminology needs to be addressed and the methods should describe the computation of fiber length density.

We thank the reviewer for pointing out this inconsistency. We have revised the Methods section (Page 10 in the Track Changes version) to clarify that the quantity estimated is fiber length density, not total fiber length. The heading has been modified to “Estimation of fiber length density,” and the text now explicitly defines fiber length density as the total length of immunoreactive fibers per unit volume of tissue (μm/μm^3), estimated stereologically using the space ball probe. We also clarify how this measure was computed and corrected for tissue shrinkage.

In addition, we now explicitly distinguish between fiber length density and varicosity density, emphasizing that fiber length density serves as an anatomical descriptor of axonal abundance and laminar organization, whereas varicosity density is the parameter used to constrain release site placement in the computational model. These revisions remove the previous ambiguity in terminology and ensure consistency between Methods and Results.

Specific comments:

Page 2 – “low spatial precision” - Low spatial precision - volume transmission could cite Descarries, whereas synaptic could cite the German anatomist Frotscher

We thank the reviewer for this helpful suggestion. We have revised the sentence to more clearly distinguish between the traditional view of neuromodulation as spatially diffuse (Descarries, 2004) and anatomical evidence for classical synaptic contacts (Frotscher & Léránth, 1985).

Page 2 – “ranging from 14 to 16%” - Cite the original research not just the review?

We agree and have added the primary experimental study (Turrini et al., 2001) to directly support the reported percentage values.

Page 2 – “point-to-point modulation” - Give more detail - what do they mean by point to point modulation of synaptic transmission? Do they mean ACh mediated presynaptic inhibition??? Give details of Kalmbach

We thank the reviewer for requesting a clarification. We have revised the Introduction to specify that this refers to synapse-specific cholinergic modulation, as demonstrated by Kalmbach et al. (2012). In particular, we now explicitly state that acetylcholine can enhance glutamatergic transmission via presynaptic nicotinic receptors in a synapse-specific manner, selectively facilitating transmitter release at defined cortical synapses rather than producing a global neuromodulatory effect.

Page 3 – “long standing debate” - This could be laid out more clearly as the overview of synaptic versus volume transmission is spread over several paragraphs. Also, they should note that despite the presence of cholinesterases, experimental work in the past has demonstrated that ACh could be collected at the cortical surface which is quite a distance from release sites. ??

We agree with the reviewer that the distinction between synaptic and volume transmission is a central theme that benefits from a clearer overview. We have reorganized the Introduction to present this topic more cohesively.

Specifically, we have added a new paragraph (Lines 152-162 in the Track Changes version) explicitly stating that the ambiguity in this "long-standing debate" stems largely from the limitations of traditional 2D ultrastructural studies. We emphasize that conclusive resolution on the proportion of synaptic vs. volume transmission awaits future large-scale 3D EM data.

Page 3 – “both DA and 5HT receptors” - It has also been shown in slice preparations that cortical neurons respond to serotonin and to dopamine, so this provides physiological data for the influence of these modulators.

We thank the reviewer for this suggestion. We have revised the manuscript to include references to slice electrophysiology studies demonstrating that cortical neurons respond to dopamine and serotonin. Specifically, we now cite Araneda & Andrade (1991) and Onn et al. (2006), which provide physiological evidence of DA- and 5-HT-mediated effects in cortical slice preparations. This addition clarifies that the influence of these neuromodulators is supported not only by receptor localization studies but also by functional electrophysiological data.

Page 3 – “estimated the densities and laminar distribution” - Hasn't the Allen brain institute published anatomical slices of neuromodulatory input to somatosensory cortex? They ought to cite previous studies on this topic.

The reviewer is correct that the Allen Brain Institute has published extensive anatomical data on neuromodulatory inputs to the somatosensory cortex, specifically within the Allen Mouse Brain Connectivity Atlas.

However, we have chosen not to include a detailed comparison with these datasets in the manuscript for two main reasons rooted in biological specificity:

1. The Allen Institute's standard datasets are derived from adult mice (P56+), whereas our study explicitly investigates juvenile rats (2 weeks old). The density and laminar distribution of neuromodulatory fibers undergo significant refinement during this developmental window (from P14 to adulthood). Consequently, comparing our juvenile data against an adult reference atlas would equate to comparing two structurally distinct developmental stages, which could lead to misleading conclusions regarding circuit organization.
2. Our work focuses on the Wistar rat model. While mouse data serves as a valuable general reference, direct quantitative extrapolation of fiber densities between species—particularly at different ages—is not methodologically sound for the specific claims we are making.

Therefore, our specific density and laminar distribution estimates in the juvenile rat were necessary and non-redundant. We hope this clarification explains our rationale for focusing the modeling on our own data.

Page 3 - "suggesting that ACh signaling is consistent with synaptic transmission" – This is a completely confusing statement. What does this mean? Are they saying that ACh signaling is using synaptic transmission instead of volume transmission? If so, they should say “consistent with synaptic transmission versus volume transmission.”

We thank the reviewer for pointing out that the original wording was ambiguous. We have revised the sentence to explicitly state “consistent with synaptic transmission versus volume transmission” to avoid confusion. Our intention was to indicate that, within the assumptions of our model, the network effects of acetylcholine more closely resemble those produced by synaptic transmission than by purely diffuse volume transmission. The revised wording now makes this comparison explicit.

page 5 - “number of sampling sites” - they should give an overview of this technique and note what this quantitative restriction means. Does it mean a random sampling of a very small number of varicosities or is it exhaustively testing almost all fibers in the cortical tissue?

Following previous studies (Mouton et al., 2002), we used stereological techniques to estimate in 3D the length of fibres and the number of varicosities per cortex unit volume, for cholinergic, catecholaminergic, and serotonergic neuromodulatory systems. Regarding the sampling to estimate the density of varicosities, we included the description in the Methods: “For each cortical layer, type of immunostaining and animal, the number of sampling sites performed and the number of sections used was determined by the constraints of maintaining the coefficient of error below 0.09 (Gundersen, 1988).”

Page 9 – “stacked horizontally” – what do they mean – that they are all laid out in a one-dimensional row?

We thank the reviewer for pointing out this ambiguity. By “stacked horizontally,” we did not mean that the microcircuits are arranged in a one-dimensional row of neurons. Instead, the term refers to the spatial arrangement of multiple cortical microcircuits tiled side by side in the horizontal plane to form a larger cortical sheet. We have revised the text to explicitly describe this geometry and avoid confusion.

Page 9 - “near the transition from the synchronous to the asynchronous state” – They could provide a bit more detail about this. What were the significant parameters that caused the state change? They next say the circuit is oscillating at 2 Hz, but they should state explicitly if this is what they termed the synchronous state?

We thank the reviewer for requesting clarification. In our simulations, the “synchronous state” refers to a regime characterized by slow, coherent network oscillations (~2 Hz), which we use to approximate an inactivated or sleep-like cortical state. This regime is controlled by the phenomenological parameters governing extracellular calcium and potassium concentrations, which modulate synaptic release probability and neuronal excitability, respectively. We have revised the Methods section to explicitly define the synchronous state and to clarify that the ~2 Hz oscillatory regime corresponds to this condition.

Page 9 – “to compute the delta power, we selected” – How was this done? Was it with band pass filtering?

We thank the reviewer for requesting clarification. Delta power was computed from the power spectral density (PSD) obtained using the Welch method, rather than via time-domain band-pass filtering. Specifically, after computing the PSD of the population firing rate, we quantified delta power by integrating the spectral power within the 1–3 Hz frequency band. We have revised the Methods section to explicitly describe this procedure (Lines 527-530 in the Track Changes version).

Figure 3 – The TH fiber length is shorter than the ChAT fiber length – it appears that this could partly be due to an effect of the orientation of fibers relative to the slicing plane, rather than an anatomical difference in fibers. It should be noted in the text whether the orientation of the fibers would impact the estimates of density or if the sampling prevents this.

We thank the reviewer for raising this important methodological point. Fiber length density was estimated using the space ball stereological probe, which is specifically designed to provide orientation-independent estimates of fiber length. Because the method samples isotropically within the tissue volume, differences in fiber orientation relative to the slicing plane do not bias the length density measurements. We have clarified this point in the Methods section to emphasize that the observed differences in fiber length density between TH- and ChAT-positive fibers are unlikely to arise from orientation artifacts (Lines 322-324 in the Track Changes version).

Page 14 – “innervated by each projection system” – aren’t there some estimates from the EM studies by Descarries about the actual proportion of contacts made by varicosities onto different cell types?

The ultrastructural studies by Descarries and colleagues (e.g., Umbriaco et al., 1994) quantified the proportion of cholinergic varicosities forming classical synaptic contacts and described their laminar distribution. However, these studies did not provide systematic cell-type-specific quantification of postsynaptic targets (e.g., excitatory versus inhibitory neurons or specific m-types). As a result, the available EM data do not allow direct inference of the proportion of contacts onto defined neuronal cell types. In our model, estimates of cell-type innervation are therefore derived from experimentally measured varicosity densities combined with the spatial distribution and dendritic geometry of reconstructed neuronal morphologies, rather than from EM-derived cell-type-specific targeting probabilities. We have clarified this distinction in the manuscript to avoid potential confusion (Lines 617-620 in the Track Changes version).

Figure 5E – what is the “cumulative frequency” plotted in the middle for release of ACh? This is not clear and should be discussed explicitly in the figure legend and text. The plot for dopamine makes more sense in terms of showing a decrease in concentration with greater distance from release.

We thank the reviewer for pointing out this lack of clarity. The middle panel in Figure 5E shows data replotted from Yamasaki et al. (2010) and does not represent a probability density or local concentration profile of M1 receptors. Instead, it represents the cumulative distribution (integral) of M1 receptor labeling as a function of distance from the acetylcholine release site.

More specifically, the plotted “cumulative frequency” corresponds to the integral sum of the receptor-associated signal within a growing radius from the release site. As such, the slope (derivative) of this curve, rather than the curve itself, reflects the spatial distribution of receptor labeling density. Consequently, the plateau region of the curve indicates that additional distance contributes negligible additional receptor signal, implying that the local receptor density approaches zero beyond that range.

We agree that this distinction was not sufficiently clear in the original manuscript. We have therefore revised the figure legend and the accompanying text to explicitly state that this plot represents a cumulative distribution and to clarify how it should be interpreted in the context of spatial extent of cholinergic volume transmission.

Page 18 “cholinergic stimuli in a physiologically relevant range” – They should have more discussion of how they chose what study to focus on. Did these optogenetic studies of ACh release measure membrane conductances intracellularly? The computational models need explicit information on membrane conductances, so these in vivo studies might just be an intermediate stage for modeling, but they probably do not provide voltage clamp data of the sort needed for computational models. They should describe the in vivo studies as an intermediate link to the important studies of membrane conductances using bath application effects in slices?

We thank the reviewer for this important clarification. We agree that optogenetic studies of cholinergic release in vivo generally do not provide direct voltage-clamp measurements of membrane conductances and therefore cannot, on their own, parameterize a conductance-based computational model. Our intent in emphasizing these studies was not to derive conductance values from them, but to define what we consider a physiologically relevant regime of cholinergic activation—namely, levels achieved by endogenous release through optogenetic stimulation or relatively low concentrations of agonists, rather than supraphysiological bath application.

Within our framework, in vivo optogenetic studies serve as a constraint on the operational range and functional impact of cholinergic modulation at the circuit level (e.g., desynchronization strength, cell-type specificity). The quantitative conductance parameters implemented in the model, however, are informed by intracellular and voltage-clamp studies in slice preparations using bath or focal application of cholinergic agents. Thus, in vivo studies provide a systems-level reference for physiological relevance, while slice electrophysiology provides the conductance-level constraints required for modeling. We have clarified this hierarchy of evidence in the manuscript to avoid ambiguity regarding the sources of conductance parameterization (Lines 895-907 in the Track Changes version).

Page 27 – The table at the top of this page doesn’t seem to have a number or a legend (maybe because it is cut off on the left). In addition, they should provide citations for the labels “data/structuring assumption”

We thank the reviewer for pointing this out. The table has now been correctly numbered (Table 9) and a legend has been added to clarify its content. We have also revised the table to avoid ambiguity in the classification of assumption types and included references where applicable (e.g., Markram et al., 2015). In particular, assumptions labeled as “data/structuring assumptions”, are now clearly defined as modeling choices that arise from how experimental data are translated into circuit-level parameters. These revisions improve clarity and ensure that the rationale behind each assumption is transparent.

Page 27 - “well known dynamics of neocortical synapses” – this is a huge literature. They should mention that Markram et al., 2015 is a review, not the source of the primary data.

We thank the reviewer for this important clarification. We agree that the dynamics of neocortical synapses are supported by a large body of primary experimental literature, and that Markram et al. (2015) synthesizes rather than originates these data. In the revised manuscript, we now clarify that the synaptic dynamics implemented in our model follow the classification framework described in Markram et al. (2015), which integrates and parameterizes short-term plasticity data from multiple primary experimental studies. We have added references to foundational work on short-term synaptic dynamics (e.g., Tsodyks & Markram, 1997; Abbott & Regehr, 2004) to more accurately reflect the underlying primary literature.

Page 32 – Don’t the parameters in this table 2 come from a source other than Markram et al., 2015? This should be clarified as there are many experimental studies of synaptic dynamics in the cortex.

We thank the reviewer for this important clarification. The synaptic *classification framework* (E1, E2, I2, I3) used in our model follows the Neocortical Microcircuit (NMC) implementation described in Markram et al. (2015), which organizes synaptic dynamics into canonical classes based on extensive primary electrophysiological data. However, the specific parameter values used for neuromodulatory synapses (e.g., decay time constants, utilization, depression and facilitation time constants) were not derived solely from Markram et al. (2015). Where available, these parameters were constrained using experimental studies reporting neuromodulator-specific synaptic kinetics (as cited in Table 2). We have revised the manuscript to clarify this distinction, making explicit that Markram et al.

(2015) provides the structural synaptic framework, while parameter values are informed by primary experimental literature where available (Lines 1185-1188 in the Track Changes version).

Table 5 – “studies using electrical stimulation of subcortical nuclei or bath-application of neuromodulators as methods to evoke neuromodulator release.” I think the phrase “or bath-application of neuromodulators” should be moved AFTER evoke neuromodulator release, as bath application of neuromodulators is not a method to evoke neuromodulator release.

We thank the reviewer for pointing out this imprecision. We have revised the sentence to clarify that electrical stimulation was used to evoke endogenous neuromodulator release, whereas bath application refers to exogenous application of neuromodulators. The wording has been corrected accordingly.
