# Supplementary material for: Quantitative anatomy and biophysical modeling of ascending neuromodulatory systems in the developing rat neocortex
Source: PLoS Comput Biol. 2026 Jun 26;22(6):e1014460. doi: 10.1371/journal.pcbi.1014460 (PMC13345462; doi:10.1371/journal.pcbi.1014460)
Supplement: Attachment — Submitted filename: Response_to_reviewers_v2.pdf [file pcbi.1014460.s006.pdf]

PCOMPBIOL-D-25-01586R1

Answers to Editor’s and Reviewers’ comments

Dear Dr. Antonietti,

Thank you for submitting your manuscript to PLOS Computational Biology. After careful consideration, we feel that it has merit but does not fully meet PLOS Computational Biology's publication criteria as it currently stands. Therefore, we invite you to submit a revised version of the manuscript that addresses the points raised during the review process.

Please submit your revised manuscript by Jun 19 2026 11:59PM. If you will need more time than this to complete your revisions, please reply to this message or contact the journal office at ploscompbiol@plos.org. When you're ready to submit your revision, log on to https://www.editorialmanager.com/pcompbiol/ and select the 'Submissions Needing Revision' folder to locate your manuscript file.

Please include the following items when submitting your revised manuscript:

* A letter that responds to each point raised by the editor and reviewer(s). You should upload this letter as a separate file labeled 'Response to Reviewers'. This file does not need to include responses to formatting updates and technical items listed in the 'Journal Requirements' section below.
* A marked-up copy of your manuscript that highlights changes made to the original version. You should upload this as a separate file labeled 'Revised Manuscript with Track Changes'.
* An unmarked version of your revised paper without tracked changes. You should upload this as a separate file labeled 'Manuscript'.

If you would like to make changes to your financial disclosure, competing interests statement, or data availability statement, please make these updates within the submission form at the time of resubmission. Guidelines for resubmitting your figure files are available below the reviewer comments at the end of this letter.

We look forward to receiving your revised manuscript.

Kind regards,

Roman Bauer
Academic Editor
PLOS Computational Biology

Andrea E. Martin
Section Editor
PLOS Computational Biology

Additional Editor Comments:

Please address the comments of the reviewers. Also, please justify better and explain why "the simulation framework and computational infrastructure used for this study are neither available nor accessible", as this sounds indeed like this study can never be replicated.

We thank the reviewers for their comments on the revised manuscript. We apologise for the ambiguous wording in the response letter, which could be misinterpreted that the study cannot be replicated. This is clearly not the case.

To clarify, the data, model files, parameterizations, simulation configurations, and analysis code required to reproduce the simulations reported in this manuscript are publicly available on Zenodo. Therefore, the results presented in the paper are fully reproducible. However, what is no longer available is the dedicated high-performance computing infrastructure originally used to run additional large-scale simulation campaigns, such as new ablation experiments or extensive parameter scans requested during the previous round of reviews.

1) Please ensure that the funders and grant numbers match between the Financial Disclosure field and the Funding Information tab in your submission form. Note that the funders must be provided in the same order in both places as well.

Thank you for pointing that out. We have fixed the order of funding sources in the Funding Information Tab to match the Final Disclosure statement.

Reviewer #3

Overall, I appreciate the revised version of this manuscript. As noted previously, the paper presents a useful combination of anatomical data and computational simulations of network dynamics. The strength of the paper is the anatomical quantification of neuromodulatory fibers in the somatosensory cortex arising from cholinergic, dopaminergic and serotonergic neurons. As my previous review indicated, I was concerned by the simplified nature of the computational simulations of the influence of these modulators on the oscillatory network dynamics of this cortical circuit. However, I will accept the authors rationale for these simplifications because I still think this work is important and these types of models should continue to be developed to focus on the important interaction of neuromodulatory input and network dynamics.

We thank the reviewer for their positive assessment of the revised manuscript and for acknowledging the value of the anatomical quantification and modeling framework. We appreciate their constructive perspective on the modeling simplifications and agree that future work should refine these simulations as more detailed cell-type- and conductance-specific data become available.

There are a few minor comments that should be addressed but could be evaluated by the editors:
1. Since the time of this review, there has been a new paper that supports volume transmission in the hippocampus. They should cite this paper which shows similarity of ACh dynamics recorded hundreds of microns apart using two-photon imaging of ACh fluorescence by Xuan, Li, Li and Dombeck, 2025.

We thank the reviewer for pointing us to this recent study. We have included a citation to Xuan et al. (2025), which provides new evidence consistent with volume transmission of acetylcholine in hippocampal CA1 using genetically encoded ACh fluorescence imaging. We now mention this work as additional support for the view that cholinergic signaling can exhibit spatially broad dynamics over hundreds of microns in the hippocampus as well.

2. There are a few places that refer to the Markram et al., 2015 paper in a manner that could be interpreted to indicate that the same techniques are used here. These should be modified. On line 204, they say: “provide the physiological foundation for the conductance-based implementation of cholinergic modulation in the present model.” This is misleading. They should instead say: “Provide the foundation for the more simplified conductance-based implementation of cholinergic modulation in the present model.”

We thank the reviewer for this clarification. We agree that some wording could be read as implying that the neuromodulatory implementation used the same detailed conductance-modeling procedures as Markram et al. (2015). We have revised these passages to make clear that Markram et al. (2015) provides the underlying neocortical microcircuit and synaptic-dynamics framework, whereas the neuromodulatory projections introduced here are implemented using a simplified, phenomenological conductance-based approach constrained by available literature data.

Page 4. *This model builds on the previously reconstructed neocortical microcircuit framework by adding a simplified representation of neuromodulatory fibers in the sensory cortex, allowing for the activation of these fibers and assessment of their impact on neurons within the somatosensory network (Markram et al., 2015; Ramaswamy et al., 2015, 2018; Reimann et al., 2026).*

Page 5. *These experimentally established effects on intrinsic excitability and synaptic efficacy provide the physiological foundation for the more simplified conductance-based implementation of cholinergic modulation in the present model.*

Page 5. *This phenomenological approach captures the net functional impact on membrane potential and firing probability, while being implemented on top of the biophysically detailed neuron models of the underlying neocortical microcircuit (Markram et al., 2015), without re-optimizing or directly modifying their intrinsic membrane conductances.*

Page 9. *The underlying neocortical microcircuit uses conductance-based synaptic models described in Markram et al. (2015) and in the Neocortical Microcircuit Collaboration Portal (Ramaswamy et al., 2015). In the present work, we used this framework as a base and added simplified neuromodulatory conductances whose parameters were constrained from literature-reported data.*

Page 10. *For the new NM connections, we used the synaptic type framework (s-types) defined in Markram et al., (2015) as a structuring approximation for short-term dynamics, while neuromodulator-specific decay time constants (DTCs) were constrained from the literature (see Table 2).*

3. On line 215 they say: does not attempt to explicitly model receptor subtype–specific intracellular.” After the words “does not attempt to explicitly model” they should add: “the voltage or calcium-sensitive properties of membrane conductances or receptor subtype-specific…”

Thank you for pointing this out. We have amended the text as suggested.

Page 5. *Importantly, this framework does not attempt to explicitly model the voltage or calcium-sensitive properties of membrane conductances or receptor subtype-specific intracellular signaling cascades, which remain insufficiently constrained across cell types and developmental stages.*

4. On line 235: desynchronizing effect of ACh, suggesting that ACh signaling” – This is confusing. “suggesting that Ach signaling” should be replaced with “and comparison with experimental data generates the additional suggestion that the dynamics are better matched by ACh synaptic transmission or weak volume transmission.”

We thank the reviewer for pointing out that the previous wording was unclear. We have revised the sentence as suggested

5. Methods: Animals. This should start with an introductory sentence indicating that the experimental methods focus on describing the anatomical properties of neuromodulatory fibers.

We have added an introductory sentence at the beginning of the Animals section to clarify.

6. Line 901 - “these experiments define the temporal profile, magnitude,” – This should include some mention of whether they also make their simulations based on the depolarization or hyperpolarization of cell types in response to modulation (as summarized in the tables).

We thank the reviewer for this clarification. We have revised the sentence to state explicitly that, in addition to defining the temporal profile, magnitude, and circuit context of cholinergic modulation, the electrophysiological studies summarized in Tables 5–7 also constrain the net direction of modulation across cell types. Specifically, these data were used to determine whether the implemented neuromodulatory effects should be depolarizing or hyperpolarizing for each target population.

7. Line 843 – “boutons” it is confusing that they refer here to boutons whereas they earlier say: “Data about the density of synaptic boutons, or, more specifically in our case, NM varicosities,” I think they should either use the term varicosities throughout, or distinguish between boutons and varicosities for the ST vs. VT models, or perhaps always say “boutons or varicosities”

We thank the reviewer for noting this inconsistency. We agree that alternating between “boutons” and “varicosities” could be confusing in this context. We have revised the manuscript to use “varicosities” consistently when referring to the experimentally quantified neuromodulatory structures, and “release sites” when referring to their implementation in the model. The term “boutons” has been removed to avoid ambiguity.

8. Line 856 – “profound and widespread” – Somewhere in the discussion, they should mention that volume transmission is supported by similar ACh dynamics recorded hundreds of microns apart using two-photon imaging of ACh fluorescence by Xuan, Li, Li and Dombeck, 2025.

We thank the reviewer for this suggestion. We have added Xuan et al. (2025) to the Discussion as recent experimental evidence supporting spatially broad cholinergic dynamics consistent with volume transmission. We note that this evidence comes from hippocampal CA1, while emphasizing that cortical neuromodulatory effects may still depend on a more complex interaction of spatial spread, fiber recruitment, and receptor localization.

9. Tables 5-6-7. I see these being referred to throughout the revised manuscript, but I thought the numbering scheme was changed.

Thanks for catching that error. We have removed what was Table 5 in this second round of revisions. We have revised the new Table numbers to ensure that they are correctly referenced in the whole manuscript.

10. “Moreover, the model incorporates detailed biophysical properties” – I would argue that it does NOT incorporate detailed biophysical properties. I think the word detailed should be replaced by simplified.

We thank the reviewer for this clarification, we have revised the text accordingly here (but also elsewhere as explained in the answer to reviewer’s point 2.

Page 30: *Moreover, the model incorporates key biophysical properties and anatomically grounded neuromodulatory innervation patterns, but we acknowledge that the simulated network activity does not fully recapitulate the complexity of in vivo cortical dynamics.*

11. Line 1082 – There is a whole paragraph contrasting the techniques in this paper with the Hodgkin-Huxley simulations of dopaminergic modulation by Durstewitz, Seamans and Sejnowski. However, this same limitation would apply for the way they model the cholinergic modulation. Thus, this paragraph should also include discussion of their simplified representation of effects of acetylcholine, and note that they do not directly simulate the effects of acetylcholine on the M current or AHP current as was done in detail in previous simulations (e.g. Traub, Miles, Buzsaki, 1992; Barkai, Bergman, Horwitz, Hasselmo, 1994).

We agree that the limitation discussed for dopaminergic modulation also applies to our implementation of cholinergic modulation. We have revised the paragraph to explicitly state that ACh effects are also represented phenomenologically and that the model does not directly simulate specific ionic mechanisms such as muscarinic modulation of the M-current or AHP currents.

Pages 30-31: *A similar limitation applies to our implementation of cholinergic modulation, which is likewise modeled phenomenologically. In particular, our model does not explicitly simulate the modulation of specific ionic currents such as the M-current or afterhyperpolarization (AHP) currents, which have been characterized in detail in previous conductance-based models (Traub et al., 1992; Barkai et al., 1994). Instead, both dopaminergic and cholinergic effects are represented as net changes in excitability or synaptic efficacy, constrained by experimental observations. Nevertheless, our phenomenological conductances capture the net functional outcomes documented in the experimental literature. Future work could bridge these approaches by: (1) conducting systematic optogenetic activation studies of VTA, BF, and raphe inputs to somatosensory cortex combined with cell-type-specific recordings; (2) characterizing NM receptor subtype expression across cortical layers and cell types in developing somatosensory cortex; (3) measuring receptor-specific effects on individual ionic conductances (gNa, gK, gCa, etc.) for different m-types; and (4) implementing mechanistic neuromodulation following the Durstewitz and Seamans framework once these data become available.*

Reviewer #4:

This is a revision of a manuscript that was already reviewed by three other reviewers and was extensively revised. As I have not reviewed the original submission of this manuscript, it would be unfair to the authors if I pepper them with new comments, so I will restrict my comments to those I consider most important.

We thank the reviewer for their careful and considerate assessment of the manuscript, especially given that this is a later-stage revision. We appreciate their decision to focus on the most important remaining issues and have addressed each of the comments below.

Synchronized state: Since this state is used as the baseline for all the simulations, please explain in more detail under what conditions the in silico model enters into this state. If I understand correctly the explanation on P. 12, this was achieved by depolarizing all neurons to just below their firing threshold – is this correct? If so, please say so explicitly, instead of referring to the potassium concentration that is not directly modeled anyway. Was there a precise membrane potential when the system enters this oscillatory state, or is this a range? Does the frequency of oscillations vary with the membrane potential?

We thank the reviewer for requesting this clarification. The reviewer's interpretation is correct: in our simulations, the synchronized baseline state is obtained by increasing network excitability so that neurons are depolarized close to, but below, firing threshold (i.e., at 95% of the threshold). As previously done in Markram et al., (2015), this was implemented phenomenologically by injecting a constant somatic current into each neuron, rather than by explicitly modeling extracellular potassium dynamics.

We have revised the Methods to state this explicitly. We also clarify that the synchronized state is not defined by a single fixed membrane potential. Rather, it corresponds to a range of excitability values in which recurrent interactions in the microcircuit generate coherent slow oscillations at approximately 2 Hz. Departures from this operating range altered the stability and frequency of the oscillatory regime and could move the network away from the synchronized state. All neuromodulatory simulations were performed from the same baseline synchronous condition.

Page 13: *Simulations were conducted near the transition between a synchronous and an asynchronous network regime. In this study, the synchronous state refers to a condition characterized by coherent slow oscillations (~2 Hz), approximating an inactivated or sleep-like cortical state. As previously done in Markram et al., (2015), this state was obtained by increasing network excitability through a constant somatic depolarizing current applied to all neurons, bringing cells close to, but below, their firing threshold. This manipulation is a phenomenological approximation of the effect of elevated extracellular potassium used in the underlying microcircuit framework; extracellular potassium dynamics themselves were not explicitly modeled. The synchronized state was not defined by a single membrane potential value, but by a range of depolarizing drive values over which the network produced stable slow oscillations. Changes in this depolarizing drive can alter the stability and frequency of the oscillatory regime and eventually shift the network toward asynchronous activity. All neuromodulatory stimulations were applied starting from this baseline synchronous condition.*

Nomenclature of cell types: The authors use 3 different nomenclatures for their cortical cell types, especially interneurons, in different places. In Fig. 5 they use the common neurochemical nomenclature (PV, VIP, SSt), in Fig. 6 they use the morphological nomenclature established by the Markram lab, and in Table 2 they use yet another nomenclature ("proximal targeting " and "distal targeting"). Readers should not be expected to know the correspondence between these nomenclatures – the onus is on the authors to either use the same nomenclature throughout, or to provide this correspondence in a table or figure.

We thank the reviewer for pointing out this inconsistency in cell-type nomenclature. We agree that using neurochemical classes, morphological types, and targeting-based interneuron labels in different parts of the manuscript could make the correspondence between categories unclear. We have therefore revised the manuscript to use the morphological nomenclature (m-types) of the underlying neocortical microcircuit as the primary reference, because it provides the finest granularity for the model. This terminology is inherited from the Markram et al. microcircuit framework, which defines layer-specific morphological neuron subtypes.

To improve clarity, we have expanded the legends of Figures 4 and 5 to define the morphological-type abbreviations and to indicate how broader neurochemical classes map onto these m-types. In Figure 5, we now explicitly state that PV, VIP, and SST correspond to broader interneuron classes grouping multiple m-types. In addition, we have removed the “proximal-targeting” and “distal-targeting” terminology from Table 2 and replaced it with m-type-based nomenclature. These changes provide a consistent reference framework across the figures and tables and make the correspondence between cell-type categories explicit for readers.

Table 2: Please provide the reversal potential used for each conductance – this is a critical parameter in any simulation.

We agree that the reversal potential is a critical parameter and we have added the reversal potentials to Table 2.

Table 5: The point of this table escapes me. How did the authors calculate these correlations? Correlation is defined between vectors – which vectors are being correlated here? What do these R2 values tell us? Please provide more context and explanation in the text and/or the legend.

Thank you very much for this thoughtful comment. We sincerely apologize for the confusion caused by Table 5. This table corresponded to an exploratory analysis that was included in an earlier version of the manuscript; however, the associated data and correlations are not utilized, interpreted, or discussed in the current version of the study. As a result, the necessary methodological details to fully clarify which vectors were correlated and how the reported R^2 values should be interpreted are no longer present in the text. We have now removed Table 5 from the revised version.

Have the authors made all data and (if applicable) computational code underlying the findings in their manuscript fully available?

Reviewer #4: No: According to the authors' response letter, "the simulation framework and computational infrastructure used for this study are neither available nor accessible". This sounds to me like this study can never be replicated.

We thank the reviewer for pointing out this ambiguity. Our previous wording in the response letter was imprecise and may have given the incorrect impression that the study is not reproducible. This was not our intention.

To clarify, the simulation framework, model files, parameterization, and code required to reproduce the simulations reported in this manuscript are publicly available through Zenodo. Thus, the analyses and simulations presented in the paper are reproducible. What is no longer available to us is the dedicated high-performance computing infrastructure that would be required to perform additional large-scale simulation campaigns beyond those reported here, such as the new ablation experiments or extensive parameter scans suggested during the previous round of reviews.
