# Supplementary material for: Quantitative anatomy and biophysical modeling of ascending neuromodulatory systems in the developing rat neocortex
Source: PLoS Comput Biol. 2026 Jun 26;22(6):e1014460. doi: 10.1371/journal.pcbi.1014460 (PMC13345462; doi:10.1371/journal.pcbi.1014460)
Supplement: Attachment — Submitted filename: Review of Colangelo et al.docx [file pcbi.1014460.s004.docx]

Review of Colangelo et al

This is a revision of a manuscript that was already reviewed by three other reviewers and was extensively revised. As I have not reviewed the original submission of this manuscript, it would be unfair to the authors if I pepper them with new comments, so I will restrict my comments to those I consider most important.

Synchronized state: Since this state is used as the baseline for all the simulations, please explain in more detail under what conditions the in silico model enters into this state. If I understand correctly the explanation on P. 12, this was achieved by depolarizing all neurons to just below their firing threshold – is this correct? If so, please say so explicitly, instead of referring to the potassium concentration that is not directly modeled anyway. Was there a precise membrane potential when the system enters this oscillatory state, or is this a range? Does the frequency of oscillations vary with the membrane potential?

Nomenclature of cell types: The authors use 3 different nomenclatures for their cortical cell types, especially interneurons, in different places. In Fig. 5 they use the common neurochemical nomenclature (PV, VIP, SSt), in Fig. 6 they use the morphological nomenclature established by the Markram lab, and in Table 2 they use yet another nomenclature (“proximal targeting ” and “distal targeting”). Readers should not be expected to know the correspondence between these nomenclatures – the onus is on the authors to either use the same nomenclature throughout, or to provide this correspondence in a table or figure.

Table 2: Please provide the reversal potential used for each conductance – this is a critical parameter in any simulation.

Table 5: The point of this table escapes me. How did the authors calculate these correlations? Correlation is defined between vectors – which vectors are being correlated here? What do these R2 values tell us? Please provide more context and explanation in the text and/or the legend.
