# Supplementary material for: Quantitative anatomy and biophysical modeling of ascending neuromodulatory systems in the developing rat neocortex
Source: PLoS Comput Biol. 2026 Jun 26;22(6):e1014460. doi: 10.1371/journal.pcbi.1014460 (PMC13345462; doi:10.1371/journal.pcbi.1014460)
Supplement: Peer review history

# Peer review: Decision Letter 0

**DOI:** 10.1371/journal.pcbi.1014460.r001

**Authors:** Roman Bauer, Andrea E. Martin

12 Nov 2025

-->PCOMPBIOL-D-25-01586

Quantitative anatomy and biophysical modeling of ascending neuromodulatory systems in developing rat cortex

PLOS Computational Biology

Dear Dr. Antonietti,

Thank you for submitting your manuscript to PLOS Computational Biology. After careful consideration, we feel that it has merit but does not fully meet PLOS Computational Biology's publication criteria as it currently stands. Therefore, we invite you to submit a revised version of the manuscript that addresses the points raised during the review process.

Please submit your revised manuscript by Jan 12 2026 11:59PM. If you will need more time than this to complete your revisions, please reply to this message or contact the journal office at ploscompbiol@plos.org. When you're ready to submit your revision, log on to https://www.editorialmanager.com/pcompbiol/ and select the 'Submissions Needing Revision' folder to locate your manuscript file.

Please include the following items when submitting your revised manuscript:

* A rebuttal letter that responds to each point raised by the editor and reviewer(s). You should upload this letter as a separate file labeled 'Response to Reviewers'. This file does not need to include responses to formatting updates and technical items listed in the 'Journal Requirements' section below.

* A marked-up copy of your manuscript that highlights changes made to the original version. You should upload this as a separate file labeled 'Revised Manuscript with Track Changes'.

* An unmarked version of your revised paper without tracked changes. You should upload this as a separate file labeled 'Manuscript'.

If you would like to make changes to your financial disclosure, competing interests statement, or data availability statement, please make these updates within the submission form at the time of resubmission. Guidelines for resubmitting your figure files are available below the reviewer comments at the end of this letter

We look forward to receiving your revised manuscript.

Kind regards,

Roman Bauer

Academic Editor

PLOS Computational Biology

Andrea E. Martin

Section Editor

PLOS Computational Biology

**Additional Editor Comments:**

Dear Authors,

Thank you for submitting your manuscript entitled “Quantitative anatomy and biophysical modeling of ascending neuromodulatory systems in developing rat cortex” to PloS Computational Biology. Your paper has now been evaluated by three expert reviewers.

Two reviewers recommended minor revision, noting that the study is generally well executed and of potential interest. However, the third reviewer raised substantive conceptual concerns regarding the modelling and interpretation of your results. Specifically, they commented that the manuscript would require a major restructuring to focus more clearly on the effects of neuromodulators on membrane conductances, rather than on the rather simplified concepts indicated in Tables 5–7.

In addition, there was some agreement with regards to unclarity regarding the use and interpretation of fiber length (density) in your analysis. Since this measure appears central to several of your conclusions, it will be important to clarify how it was defined, computed, and biologically interpreted.

Given these and other points, the issues extend beyond minor editorial adjustments and call for a substantial revision to improve conceptual focus and methodological clarity.

**Journal Requirements:**

1) We ask that a manuscript source file is provided at Revision. Please upload your manuscript file as a .doc, .docx, .rtf or .tex. If you are providing a .tex file, please upload it under the item type u2018LaTeX Source Fileu2019 and leave your .pdf version as the item type u2018Manuscriptu2019.

2) Please provide an Author Summary. This should appear in your manuscript between the Abstract (if applicable) and the Introduction, and should be 150-200 words long. The aim should be to make your findings accessible to a wide audience that includes both scientists and non-scientists. Sample summaries can be found on our website under Submission Guidelines:

https://journals.plos.org/ploscompbiol/s/submission-guidelines#loc-parts-of-a-submission

3) Please upload all main figures as separate Figure files in .tif or .eps format. For more information about how to convert and format your figure files please see our guidelines:

https://journals.plos.org/ploscompbiol/s/figures

4) We notice that your supplementary Figures are included in the manuscript file. Please remove them and upload them with the file type 'Supporting Information'. Please ensure that each Supporting Information file has a legend listed in the manuscript after the references list.

5) Please amend your detailed Financial Disclosure statement. This is published with the article. It must therefore be completed in full sentences and contain the exact wording you wish to be published.

- State the initials, alongside each funding source, of each author to receive each grant. For example: "This work was supported by the National Institutes of Health (####### to AM; ###### to CJ) and the National Science Foundation (###### to AM)."

- State what role the funders took in the study. If the funders had no role in your study, please state: "The funders had no role in study design, data collection and analysis, decision to publish, or preparation of the manuscript.".

If you did not receive any funding for this study, please simply state: u201cThe authors received no specific funding for this work.u201d

**Reviewers' comments:**

Reviewer's Responses to Questions

**Comments to the Authors:**

**Please note here if the review is uploaded as an attachment.**

Reviewer #1: The authors have collected a dataset quantifying the length of fibres and the number of varicosities per unit volume for cholinergic, catecholaminergic and serotonergic neurmodulatory systems in cortex. They have also done an extensive literature search to gather data on the neuromodulatory effects on cortical neurons. Armed with this knowledge they build a detailed computational model to estimate the connectivity, and investigate the effect of synaptic and volumetric transmission on the sleep-state activity of the system.

They estimate the projections from the different fibres onto the neurons, calculating the number of fibres that connect to each neuron, and how many varicosities are formed between the fibre and neuron.

The authors do not directly model the volume transmission, instead choosing to use a phenomenological model where dendrites within a radius r_max are activated. The diffusion process is not modelled, instead the receptors are activated instantly, but the channels have a slower time course, which presumably could account for any transmission delays.

Finally their simulations confirm that ACh desynchronises the sensory cortex, and show that DA 5-HT also desynchronises. In addition 5-HT induces faster oscillatory activity.

Q: On page 6 the Projectionizer tool sample all morphological segments to place synaptic release sites. Do you have fully reconstructed fibres, or is this the dendritic segments of the neurons in all the different layers that are sampled? Based on the rest of the algorithm I assume it is the dendritic segments, but it is not completely clear from the text.

(Q): From my understanding you only have density estimates, not the full fibres. If you do have the full fibres, why not use a touch detection algorithm to place the synapses?

Q (page 7): Does it matter that the fibres modelled are straight lines, and not bending and turning? I assume that because of the way you assign synapses to fibres moderate jittering of the fibres might not matter?

Q (page 7): The synaptic release sites are assigned to the different fibres. Does the number of fibres matter for the desynchronisation of the sensory cortex? (In the extreme case with just one fibre there should be a difference since all releases would then be synchronised, but within the range you considered does it differ based on the number of fibres?)

Q (page 7 and 8): Given that you simulate the network and study the synchronisation it would be instructive to see the time course of the different synapse types plotted. Right now the parameters are listed in table 2, but the effect of facilitation and depression and the decay time constants might not be immediately obvious.

Q (page 8): Why did you not include different delay dependent on the distance to the release site? (Delay difference would be small?) Do you allow two release sites to affect a target site? (Ie, how close is your phenomenological model to the real volume transmission case).

Q (page 15): From what I understand the number of synapses a neuron receives will be directly proportional to the total dendritic length, and for volume transmission it depends on dendritic length and layer variscocity density? You mention the two most contacted neuron types, but do not explain why they are most connected.

Q (Figure 4): Is it correct that there are so few target neurons in layer 1, the varicosities density is highest there? (Figure 4B)

(Figure 5E): The second and third plot show experimental data that justify your choice of r_max. Please change the wording, this is not direct validation. (Or is there a model data line missing in the plot?)

(Page 18): Your simulations nicely show desynchronisation, but there is no discussion about why there is a difference between the synaptic and volumetric transmissions. What are the mechanisms. Do the two preferentially target slightly different neurons, or is it the temporal pattern of innervation, or the time course of the synapses, that matters for the strength of the desynchronisation? Would be great to understand what it is that makes them different (given your assumptions in the model).

(Page 26): Would it be possible to find out why 5-HT promotes oscillation within a specific frequency range? Is there a subpopulation of neurons that are more active? Would ablating connection to a specific population of neurons remove this oscillation?

Minor:

Q (page 14): Would the data be more easily digested in a table? (Number of neuron targets per fibre, etc)

(Figure 5B) : Tiny figure, could be made a bit larger so it is easier to see.

(Figure 5C): Here you could include some example synaptic and volumetric transmission time courses from Table 2.

Reviewer #2: This interesting paper shows that in a computational model of rat neocortex, cholinergic, domaminergic, and serotonergic activation disrupt oscillatory dynamics, and that volume transmision has a bigger impact than synaptic transmission. Immunostaining has been used to estimate the distribution of some of the synapses modeled, with most of the model parameters coming from existing literature.

The paper is generally well written and presented.

The model is large, with many parameters, although modeling appears robust and assumptions well justified.

Some potential improvements:

Most parameters are specified as estimates within a range. Although it may not be feasible to perform a full sensitivity analysis of all parameters, it would be feasible to do so for some of the key assumptions e.g. number of serotonergic/dopaminergic fibers.

Figures 6,7,8 panel D is not sufficiently well explained, text could also be bigger in these panels.

Reviewer #3: This paper presents anatomical quantification of neuromodulatory fibers from cholinergic, dopaminergic and serotonergic neurons in the somatosensory cortex. The paper also present computational simulations of the influence of these modulators on the oscillatory dynamics of this cortical circuit. The most important problem with this paper is that the effects of neuromodulators are summarized in an oversimplified manner without any mention of the modulation of the membrane conductances that underlie these effects. This results in a highly simplistic summary of ACh effects as EPSCs and IPSCs that is completely out of keeping with the Hodkgin-Huxley equations used to simulate membrane conductances in the basic model used from the earlier paper (Markram et al., 2015). This is further itemized in the major comments below along with some additional important comments in the major and specific comments. Another important issue concerns the lack of detail about how the connectivity was estimated.

Major comments:

1. Table 5 – it is a massive oversimplification to summarize the effects of ACh as “EPSC” or “IPSC.” I find this bizarre given that most intracellular recording studies in slices have shown very slow depolarization of neurons due to blockade of the leak K+ conductance, which would not qualify as an “EPSC”. In addition, this completely ignores the effect of muscarinic receptors on blocking the K(AHP) channel or the M-current, both effects which cannot be described as an EPSC or an IPSC. Finally, it also completely ignores the large literature showing muscarinic presynaptic inhibition of glutamatergic and GABAergic synaptic transmission.

2. Table 6 – the descriptions in table 6 are slightly more sophisticated in terms of referring “increase excitability” which could correspond to an effect on a membrane conductance such as those modeled by Durtstewitz and Seamans, but I would expect a compartmental biophysical simulation to describe and simulate these effects in the Hodgkin Huxley framework which is the basis for modeling done using Neuron and presented in the earlier paper by Markram et al., 2015. I find it bizarre that there is no explicit mention of these conductances that are so essential to this type of modeling.

3. Table 7 – the summary of effects of 5-HT is similarly massively oversimplified and makes no mention of the underlying membrane conductances. In addition, there is no valid rationale for describing the effects of these modulators using completely different terminology. These differences are due to the difference in effect on a large range of different membrane conductances and this must be acknowledged and explicitly described in the paper.

4. Overall the paper was somewhat confusing because there was not a sufficient overview of the methods. They need to more clearly state if their estimates of connectivity were based purely on density of fibers and location of cell types. For example, on Page 14 – it is not clear if they were you estimating these contact probabilities purely based on density and not based on EM studies of contact probabilities by identified neurons? If you aren’t using any prior studies, you should mention that you are doing it based only on densities of fibers and neurons.

5. They need to be clearer about how the electrophysiological properties of the neurons were modeled based on the evidence from intracellular recording of neurons in slices and studies of neuromodulator effects on different membrane conductances. The introduction should note that they draw on previous intracellular recording studies showing that ACh causes nicotinic and muscarinic modulation of membrane conductances (i.e. Schwindt and Crill, Madison and Nicoll) as well as causing presynaptic inhibition of both excitatory and inhibitory synpases (Valentino and Dingledine, 1981; Behrends and ten Bruggencate, 1993; Hasselmo, 1995; Hasselmo and Schnell, 1996). These types of physiological studies were presumably used to guide the simulations of cholinergic modulation, but they do not seem to be mentioned much in the introduction.

6. Page 6 – “estimation of fiber length” – The heading and text in the methods section refers to estimation of fiber length (which the name suggests would presumably be a one-dimensional measurement) and never mentions “fiber length density” and yet the Results section focuses on fiber length density (which is presumably the number of fiber segments per unit area). This discrepancy needs to be addressed and the methods should describe the computation of fiber length density.

Specific comments:

Page 2 – “low spatial precision” - Low spatial precision - volume transmission could cite Descarries, whereas synaptic could cite the German anatomist Frotscher

Page 2 – “ranging from 14 to 16%” - Cite the original research not just the review?

Page 2 – “point-to-point modulation” - Give more detail - what do they mean by point to point modulation of synaptic transmission? Do they mean ACh mediated presynaptic inhibition??? Give details of Kalmbach

Page 3 – “long standing debate” - This could be laid out more clearly as the overview of synaptic versus volume transmission is spread over several paragraphs. Also, they should note that despite the presentence of cholinesterases, experimental work in the past has demonstrated that ACh could be collected at the cortical surface which is quite a distance from release sites.

Page 3 – “both DA and 5HT receptors” - It has also been shown in slice preparations that cortical neurons respond to serotonin and to Dopamine.

Page 3 – “estimated the densities and laminar distribution” - Hasn't the Allen brain institute published anatomical slices of neuromodulatory input to somatosensory cortex? They ought to cite previous studies on this topic.

Page 3 - "suggesting that ACh signaling is consistent with synaptic transmission" – This is a completely confusing statement. What does this mean? Are they saying that ACh signaling is using synaptic transmission instead of volume transmission? If so, they should say “consistent with synaptic transmission versus volume transmission.”

page 5 - “number of sampling sites” - they should give an overview of this technique and note what this quantitative restriction means. Does it mean a random sampling of a very small number of varicosities or is it exhaustively testing almost all fibers in the cortical tissue?

Page 9 – “stacked horizontally” – what do they mean – that they are all laid out in a one-dimensional row?

Page 9 - “near the transition from the synchronous to the asynchronous state” – They could provide a bit more detail about this. What were the significant parameters that cause the state change? They next say the circuit is oscillating at 2 Hz, but they should state explicitly if this is what they termed the synchronous state?

Page 9 – “to compute the delta power, we selected” – How was this done? Was it with band pass filtering?

Figure 3 – The TH fiber length is shorter than the ChAT fiber length – this appears to be an effect of the orientation of fibers relative to the slicing plane, rather than an anatomical difference in fibers. It should be noted in the text whether this would impact the estimates of density.

Page 14 – “innervated by each projection system” – aren’t there some estimates from the EM studies by Descarries about the actual proportion of contacts made by varicocities onto different cell types?

Figure 5E – what is the “cumulative frequency” plotted in the middle for release of ACh. This is not clear. The plot for dopamine makes more sense in terms of showing a decrease in concentration with greater distance from release.

Page 18 “cholinergic stimuli in a physiologically relevant range” – They should have more discussion of how they chose what study to focus on. Did these optogenetic studies of ACh release measure membrane conductances intracellularly? Did they ignore earlier studies of bath application effects in slices?

Page 27 – The table at the top of this page doesn’t seem to have a number or a legend (maybe because it is cut off on the left). In addition, they should provide citations for the labels “data/structuring assumption”

Page 27 - “well known dynamics of neocortical synapses” – this is a huge literature. They should mention that Markram et al., 2015 is a review, not the source of the primary data.

Page 32 – Don’t the parameters in this table 2 come from a source other than Markram et al., 2015? This should be clarified as there are many experimental studies of synaptic dynamics in cortex.

Table 5 – “studies using electrical stimulation of subcortical nuclei or bath-application of neuromodulators as methods to evoke neuromodulator release.” I think the phrase “or bath-application of neuromodulators” should be moved AFTER evoke neuromodulator release, as bath application of neuromodulators is not a method to evoke neuromodulator release.

**********

**Have the authors made all data and (if applicable) computational code underlying the findings in their manuscript fully available?**

The PLOS Data policy requires authors to make all data and code underlying the findings described in their manuscript fully available without restriction, with rare exception (please refer to the Data Availability Statement in the manuscript PDF file). The data and code should be provided as part of the manuscript or its supporting information, or deposited to a public repository. For example, in addition to summary statistics, the data points behind means, medians and variance measures should be available. If there are restrictions on publicly sharing data or code —e.g. participant privacy or use of data from a third party—those must be specified.

Reviewer #1: Yes

Reviewer #2: Yes

Reviewer #3: **No:**

**********

PLOS authors have the option to publish the peer review history of their article (what does this mean?). If published, this will include your full peer review and any attached files.

If you choose “no”, your identity will remain anonymous but your review may still be made public.

**Do you want your identity to be public for this peer review?**  For information about this choice, including consent withdrawal, please see our Privacy Policy.

Reviewer #1: No

Reviewer #2: No

Reviewer #3: No

[NOTE: If reviewer comments were submitted as an attachment file, they will be attached to this email and accessible via the submission site. Please log into your account, locate the manuscript record, and check for the action link "View Attachments". If this link does not appear, there are no attachment files.]

**Figure resubmission:**

While revising your submission, we strongly recommend that you use PLOS’s NAAS tool (https://ngplosjournals.pagemajik.ai/artanalysis) to test your figure files. NAAS can convert your figure files to the TIFF file type and meet basic requirements (such as print size, resolution), or provide you with a report on issues that do not meet our requirements and that NAAS cannot fix.-->

After uploading your figures to PLOS’s NAAS tool - https://ngplosjournals.pagemajik.ai/artanalysis, NAAS will process the files provided and display the results in the "Uploaded Files" section of the page as the processing is complete. If the uploaded figures meet our requirements (or NAAS is able to fix the files to meet our requirements), the figure will be marked as "fixed" above. If NAAS is unable to fix the files, a red "failed" label will appear above. When NAAS has confirmed that the figure files meet our requirements, please download the file via the download option, and include these NAAS processed figure files when submitting your revised manuscript.

**Reproducibility:**

To enhance the reproducibility of your results, we recommend that authors of applicable studies deposit laboratory protocols in protocols.io, where a protocol can be assigned its own identifier (DOI) such that it can be cited independently in the future. Additionally, PLOS ONE offers an option to publish peer-reviewed clinical study protocols. Read more information on sharing protocols at https://plos.org/protocols?utm_medium=editorial-email&utm_source=authorletters&utm_campaign=protocols-->

# Author comment: Author response to Decision Letter 1

**DOI:** 10.1371/journal.pcbi.1014460.r002

13 Feb 2026

Attachment Submitted filename: Rebuttal PCOMPBIOL ACh.pdf

# Peer review: Decision Letter 1

**DOI:** 10.1371/journal.pcbi.1014460.r003

**Authors:** Roman Bauer, Andrea E. Martin, Roman Bauer, Andrea E. Martin

19 Apr 2026

PCOMPBIOL-D-25-01586R1

Quantitative anatomy and biophysical modeling of ascending neuromodulatory systems in the developing rat neocortex

PLOS Computational Biology

Dear Dr. Antonietti,

Thank you for submitting your manuscript to PLOS Computational Biology. After careful consideration, we feel that it has merit but does not fully meet PLOS Computational Biology's publication criteria as it currently stands. Therefore, we invite you to submit a revised version of the manuscript that addresses the points raised during the review process.

Please submit your revised manuscript by Jun 19 2026 11:59PM. If you will need more time than this to complete your revisions, please reply to this message or contact the journal office at ploscompbiol@plos.org. When you're ready to submit your revision, log on to https://www.editorialmanager.com/pcompbiol/ and select the 'Submissions Needing Revision' folder to locate your manuscript file.

Please include the following items when submitting your revised manuscript:

* A letter that responds to each point raised by the editor and reviewer(s). You should upload this letter as a separate file labeled 'Response to Reviewers'. This file does not need to include responses to formatting updates and technical items listed in the 'Journal Requirements' section below.

* A marked-up copy of your manuscript that highlights changes made to the original version. You should upload this as a separate file labeled 'Revised Manuscript with Track Changes'.

* An unmarked version of your revised paper without tracked changes. You should upload this as a separate file labeled 'Manuscript'.

If you would like to make changes to your financial disclosure, competing interests statement, or data availability statement, please make these updates within the submission form at the time of resubmission. Guidelines for resubmitting your figure files are available below the reviewer comments at the end of this letter.

We look forward to receiving your revised manuscript.

Kind regards,

Roman Bauer

Academic Editor

PLOS Computational Biology

Andrea E. Martin

Section Editor

PLOS Computational Biology

**Additional Editor Comments:**

Please address the comments of the reviewers. Also, please justify better and explain why "the simulation framework and computational infrastructure used for this study are neither available nor accessible", as this sounds indeed like this study can never be replicated.

**Journal Requirements:**

If the reviewer comments include a recommendation to cite specific previously published works, please review and evaluate these publications to determine whether they are relevant and should be cited. There is no requirement to cite these works unless the editor has indicated otherwise.

1) Please ensure that the funders and grant numbers match between the Financial Disclosure field and the Funding Information tab in your submission form. Note that the funders must be provided in the same order in both places as well.

**Reviewers' comments:**

Reviewer's Responses to Questions

**Comments to the Authors:**

**Please note here if the review is uploaded as an attachment.**

Reviewer #2: Thank you for addressing my comments.

Reviewer #3: Overall, I appreciate the revised version of this manuscript. As noted previously, the paper presents a useful combination of anatomical data and computational simulations of network dynamics. The strength of the paper is the anatomical quantification of neuromodulatory fibers in the somatosensory cortex arising from cholinergic, dopaminergic and serotonergic neurons. As my previous review indicated, I was concerned by the simplified nature of the computational simulations of the influence of these modulators on the oscillatory network dynamics of this cortical circuit. However, I will accept the authors rationale for these simplifications because I still think this work is important and these types of models should continue to be developed to focus on the important interaction of neuromodulatory input and network dynamics.

There are a few minor comments that should be addressed but could be evaluated by the editors:

1. Since the time of this review, there has been a new paper that supports volume transmission in the hippocampus. They should cite this paper which shows similarity of ACh dynamics recorded hundreds of microns apart using two-photon imaging of ACh fluorescence by Xuan, Li, Li and Dombeck, 2025.

2. There are a few places that refer to the Markram et al., 2015 paper in a manner that could be interpreted to indicate that the same techniques are used here. These should be modified. On line 204, they say: “provide the physiological foundation for the conductance-based implementation of cholinergic modulation in the present model.” This is misleading. They should instead say: “Provide the foundation for the more simplified conductance-based implementation of cholinergic modulation in the present model.”

3. On line 215 they say: does not attempt to explicitly model receptor subtype–specific intracellular.” After the words “does not attempt to explicitly model” they should add: “the voltage or calcium-sensitive properties of membrane conductances or receptor subtype-specific…”

4. On line 235: desynchronizing effect of ACh, suggesting that ACh signaling” – This is confusing. “suggesting that Ach signaling” should be replaced with “and comparison with experimental data generates the additional suggestion that the dynamics are better matched by ACh synaptic transmission or weak volume transmission.”

5. Methods: Animals. This should start with an introductory sentence indicating that the experimental methods focus on describing the anatomical properties of neuromodulatory fibers.

6. Line 901 - “these experiments define the temporal profile, magnitude,” – This should include some mention of whether they also make their simulations based on the depolarization or hyperpolarization of cell types in response to modulation (as summarized in the tables).

7. Line 843 – “boutons” it is confusing that they refer here to boutons whereas they earlier say: “Data about the density of synaptic boutons, or, more specifically in our case, NM varicosities,” I think they should either use the term varicosities throughout, or distinguish between boutons and varicosities for the ST vs. VT models, or perhaps always say “boutons or varicosities”

8. Line 856 – “profound and widespread” – Somewhere in the discussion, they should mention that volume transmission is supported by similar ACh dynamics recorded hundreds of microns apart using two-photon imaging of ACh fluorescence by Xuan, Li, Li and Dombeck, 2025.

9. Tables 5-6-7. I see these being referred to throughout the revised manuscript, but I thought the numbering scheme was changed.

10. “Moreover, the model incorporates detailed biophysical properties” – I would argue that it does NOT incorporate detailed biophysical properties. I think the word detailed should be replaced by simplified.

11. Line 1082 – There is a whole paragraph contrasting the techniques in this paper with the Hodgkin-Huxley simulations of dopaminergic modulation by Durstewitz, Seamans and Sejnowski. However, this same limitation would apply for the way the model the cholinergic modulation. Thus, this paragraph should also include discussion of their simplified representation of effects of acetylcholine, and note that they do not directly simulate the effects of acetylcholine on the M current or AHP current as was done in detail in previous simulations (e.g. Traub, Miles, Buzsaki, 1992; Barkai, Bergman, Horwitz, Hasselmo, 1994).

Reviewer #4: This is a revision of a manuscript that was already reviewed by three other reviewers and was extensively revised. As I have not reviewed the original submission of this manuscript, it would be unfair to the authors if I pepper them with new comments, so I will restrict my comments to those I consider most important.

Synchronized state: Since this state is used as the baseline for all the simulations, please explain in more detail under what conditions the in silico model enters into this state. If I understand correctly the explanation on P. 12, this was achieved by depolarizing all neurons to just below their firing threshold – is this correct? If so, please say so explicitly, instead of referring to the potassium concentration that is not directly modeled anyway. Was there a precise membrane potential when the system enters this oscillatory state, or is this a range? Does the frequency of oscillations vary with the membrane potential?

Nomenclature of cell types: The authors use 3 different nomenclatures for their cortical cell types, especially interneurons, in different places. In Fig. 5 they use the common neurochemical nomenclature (PV, VIP, SSt), in Fig. 6 they use the morphological nomenclature established by the Markram lab, and in Table 2 they use yet another nomenclature (“proximal targeting ” and “distal targeting”). Readers should not be expected to know the correspondence between these nomenclatures – the onus is on the authors to either use the same nomenclature throughout, or to provide this correspondence in a table or figure.

Table 2: Please provide the reversal potential used for each conductance – this is a critical parameter in any simulation.

Table 5: The point of this table escapes me. Correlation is defined between vectors – which vectors are being correlated here? What do these R2 values tell us? Please provide more context and explanation in the text and/or the legend.

**********

**Have the authors made all data and (if applicable) computational code underlying the findings in their manuscript fully available?**

The PLOS Data policy requires authors to make all data and code underlying the findings described in their manuscript fully available without restriction, with rare exception (please refer to the Data Availability Statement in the manuscript PDF file). The data and code should be provided as part of the manuscript or its supporting information, or deposited to a public repository. For example, in addition to summary statistics, the data points behind means, medians and variance measures should be available. If there are restrictions on publicly sharing data or code —e.g. participant privacy or use of data from a third party—those must be specified.

Reviewer #2: **No:**

Reviewer #3: Yes

Reviewer #4: **No:**  According to the authors' response letter, "the simulation framework and computational infrastructure used for

this study are neither available nor accessible". This sounds to me like this study can never be replicated.

**********

PLOS authors have the option to publish the peer review history of their article (what does this mean?). If published, this will include your full peer review and any attached files.

If you choose “no”, your identity will remain anonymous but your review may still be made public.

**Do you want your identity to be public for this peer review?**  For information about this choice, including consent withdrawal, please see our Privacy Policy.

Reviewer #2: No

Reviewer #3: No

Reviewer #4: **Yes:**  Ariel Agmon

[NOTE: If reviewer comments were submitted as an attachment file, they will be attached to this email and accessible via the submission site. Please log into your account, locate the manuscript record, and check for the action link "View Attachments". If this link does not appear, there are no attachment files.]

**Figure resubmission:**

-->While revising your submission, we strongly recommend that you use PLOS’s NAAS tool (https://ngplosjournals.pagemajik.ai/artanalysis) to test your figure files. NAAS can convert your figure files to the TIFF file type and meet basic requirements (such as print size, resolution), or provide you with a report on issues that do not meet our requirements and that NAAS cannot fix.-->

After uploading your figures to PLOS’s NAAS tool - https://ngplosjournals.pagemajik.ai/artanalysis, NAAS will process the files provided and display the results in the "Uploaded Files" section of the page as the processing is complete. If the uploaded figures meet our requirements (or NAAS is able to fix the files to meet our requirements), the figure will be marked as "fixed" above. If NAAS is unable to fix the files, a red "failed" label will appear above. When NAAS has confirmed that the figure files meet our requirements, please download the file via the download option, and include these NAAS processed figure files when submitting your revised manuscript.

**Reproducibility:**

To enhance the reproducibility of your results, we recommend that authors of applicable studies deposit laboratory protocols in protocols.io, where a protocol can be assigned its own identifier (DOI) such that it can be cited independently in the future. Additionally, PLOS ONE offers an option to publish peer-reviewed clinical study protocols. Read more information on sharing protocols at https://plos.org/protocols?utm_medium=editorial-email&utm_source=authorletters&utm_campaign=protocols

Attachment Submitted filename: Review of Colangelo et al.docx

# Author comment: Author response to Decision Letter 2

**DOI:** 10.1371/journal.pcbi.1014460.r004

4 Jun 2026

Attachment Submitted filename: Response_to_reviewers_v2.pdf

# Editor report: Decision Letter 2

**DOI:** 10.1371/journal.pcbi.1014460.r005

**Authors:** Roman Bauer, Andrea E. Martin, Roman Bauer, Andrea E. Martin, Roman Bauer, Andrea E. Martin

15 Jun 2026

Dear Dr. Antonietti,

We are pleased to inform you that your manuscript 'Quantitative anatomy and biophysical modeling of ascending neuromodulatory systems in the developing rat neocortex' has been provisionally accepted for publication in PLOS Computational Biology.

Before your manuscript can be formally accepted you will need to complete some formatting changes, which you will receive in a follow up email. A member of our team will be in touch with a set of requests.

Please note that your manuscript will not be scheduled for publication until you have made the required changes, so a swift response is appreciated.

IMPORTANT: The editorial review process is now complete. PLOS will only permit corrections to spelling, formatting or significant scientific errors from this point onwards. Requests for major changes, or any which affect the scientific understanding of your work, will cause delays to the publication date of your manuscript.

Should you, your institution's press office or the journal office choose to press release your paper, you will automatically be opted out of early publication. We ask that you notify us now if you or your institution is planning to press release the article. All press must be co-ordinated with PLOS.

Thank you again for supporting Open Access publishing; we are looking forward to publishing your work in PLOS Computational Biology.

Best regards,

Roman Bauer

Academic Editor

PLOS Computational Biology

Andrea E. Martin

Section Editor

PLOS Computational Biology

***********************************************************

# Editor report: Acceptance letter

**DOI:** 10.1371/journal.pcbi.1014460.r006

**Authors:** Roman Bauer, Andrea E. Martin, Roman Bauer, Andrea E. Martin, Roman Bauer, Andrea E. Martin

PCOMPBIOL-D-25-01586R2

Quantitative anatomy and biophysical modeling of ascending neuromodulatory systems in the developing rat neocortex

Dear Dr Antonietti,

I am pleased to inform you that your manuscript has been formally accepted for publication in PLOS Computational Biology. Your manuscript is now with our production department and you will be notified of the publication date in due course.

The corresponding author will soon be receiving a typeset proof for review, to ensure errors have not been introduced during production. Please review the PDF proof of your manuscript carefully, as this is the last chance to correct any errors. Please note that major changes, or those which affect the scientific understanding of the work, will likely cause delays to the publication date of your manuscript.

Soon after your final files are uploaded, unless you have opted out, the early version of your manuscript will be published online. The date of the early version will be your article's publication date. The final article will be published to the same URL, and all versions of the paper will be accessible to readers.

For Research, Software, and Methods articles, you will receive an invoice from PLOS for your publication fee after your manuscript has reached the completed accept phase. If you receive an email requesting payment before acceptance or for any other service, this may be a phishing scheme. Learn how to identify phishing emails and protect your accounts at https://explore.plos.org/phishing.

Thank you again for supporting PLOS Computational Biology and open-access publishing. We are looking forward to publishing your work!

With kind regards,

Sharmila Kamatchi

PLOS Computational Biology | Carlyle House, Carlyle Road, Cambridge CB4 3DN | United Kingdom ploscompbiol@plos.org | Phone +44 (0) 1223-442824 | ploscompbiol.org | @PLOSCompBiol
